# Synthesis and In Vitro Antitumor Activity of Novel Bivalent *β*-Carboline-3-carboxylic Acid Derivatives with DNA as a Potential Target

**DOI:** 10.3390/ijms19103179

**Published:** 2018-10-15

**Authors:** Hongling Gu, Na Li, Jiangkun Dai, Yaxi Xi, Shijun Wang, Junru Wang

**Affiliations:** 1 College of Chemistry and Pharmacy, Northwest A&F University, No. 22 Xinong Road, Yangling 712100, China; honglingguw@163.com (H.G.); lnuk@nwsuaf.edu.cn (N.L.); daijkun@hotmail.com (J.D.); mysteryxi@163.com (Y.X.); 444795415@nwafu.edu.cn (S.W.); 2 State key Laboratory of Drug Research, Shanghai Institute of Materia Medica, Chinese Academy of Sciences, 555 Zu Chong Zhi Road, Zhangjiang Hi-Tech Park, Shanghai 201203, China

**Keywords:** bivalent *β*-carbolines, antitumor, apoptosis, DNA-binding affinity, *bcl*-2

## Abstract

A series of novel bivalent *β*-carboline derivatives were designed and synthesized, and in vitro cytotoxicity, cell apoptosis, and DNA-binding affinity were evaluated. The cytotoxic results demonstrated that most bivalent *β*-carboline derivatives exhibited stronger cytotoxicity than the corresponding monomer against the five selected tumor cell lines (A549, SGC-7901, Hela, SMMC-7721, and MCF-7), indicating that the dimerization at the *C^3^* position could enhance the antitumor activity of *β*-carbolines. Among the derivatives tested, **4B**, **6i**, **4D**, and **6u** displayed considerable cytotoxicity against A549 cell line. Furthermore, **4B**, **6i**, **4D**, and **6u** induced cell apoptosis in a dose-dependent manner, and caused cell cycle arrest at the S and G2/M phases. Moreover, the levels of cytochrome C in mitochondria, and the expressions of *bcl*-2 protein, decreased after treatment with *β*-carbolines, which indicated that **6i** and **6u** could induce mitochondria-mediated apoptosis. In addition, the results of UV-visible spectral, thermal denaturation, and molecular docking studies revealed that **4B**, **6i**, **4D**, and **6u** could bind to DNA mainly by intercalation.

## 1. Introduction

*β*-carboline alkaloids, originally isolated from Peganum harmala, are a class of natural and synthetic indole alkaloids with a tricyclic pyrido[3,4-b]indole ring system [1,2]. *β*-carbolines have multiple biological and pharmacological properties [2,3,4], of which antitumor activity is the most widely studied [5,6,7,8]. Previous studies have shown that *β*-carbolines exerted their antitumor activity mainly through intercalating into DNA [7,9,10,11,12,13], inducing apoptosis, and inhibiting topoisomerase I and II (Top I and II) [13,14,15], cyclin-dependent kinases (CDKs) [16,17], mitogen-activated protein kinase (MAPK) [18], and I-Kappa-B kinase (IKK) [19]. Particularly, parent carbolines can be inserted into DNA, which can further cause cell apoptosis [20,21]. The interaction between *β*-carboline alkaloids and DNA could cause changes in DNA conformation, which further affects DNA replication, transcription, and repair [22,23]. Furthermore, the existing studies have demonstrated that *β*-carboline could induce HepG2 cells apoptosis by down-regulating *bcl*-*2* expression [24].

DNA-targeted antitumor drugs, such as anthracyclines, acridines, and quinones, which exert antitumor activity through DNA insertion, are considered to be one of the most effective drugs in clinical applications [25]. These drugs exert antitumor activity mainly through non-covalent and covalent interactions with the minor groove, major groove, or base pairs (intercalation) of the DNA double helix. The discovery of antitumor drugs has focused on the development of new DNA-intercalating scaffolds, such as *β*-carboline derivatives [7]. Therefore, we expect to discover and develop new antitumor agents by modifying these bioactive scaffolds with appropriate substituents.

Previous structure-activity relationships (SARs) have shown that the introduction of appropriate substituents at the *C^1^*, *C^3^*, and *N^9^* positions of the *β*-carboline scaffolds could enhance antitumor activity and DNA-binding affinity [5,7,10,26,27]. It has also been reported that phenyl and heterocyclic substituted at the *C^1^* and *C^3^* positions of *β*-carbolines, respectively, have exhibited potential antitumor activity [12,28,29,30]. The introduction of methyl and benzyl substituents at the *N^9^* position of *β*-carbolines could enhance the DNA affinity [10]. Further studies have demonstrated that dimerization of small molecules by appropriate linkers could significantly improve the DNA-binding affinity. It has been found that the dimerized compounds could bind to DNA by a bis-intercalation mode, causing pronounced changes in DNA structure [31,32]. Bivalent *β*-carbolines linked at the *C^6^* position or *N^9^* position have been synthesized, and were found to be potential anti-Alzheimer agents [33]. In addition, the synthesis and evaluation of bivalent *β*-carbolines linked by 3–10 methylene units at the *N^9^* position as antitumor agents have also been reported [34]. Our group has synthesized 25 bivalent *β*-carbolines modified at the *C^1^* (-CH_3_), *C*^7^ (-OCH_3_), and *N^9^* positions (-CH_3_ and -CH_2_Ph_2_), and dimerized at the *C^2^* position, and the results showed that the dimerization could significantly increase antitumor activity of *β*-carbolines. Moreover, compounds **4A**, **6b**, **6d**, and **6e**, which exhibit good antitumor activity, have been reported [5,35]. However, the SARs of the antitumor activity in vitro of bivalent *β*-carbolines linked at the *C^3^* position were rarely reported.

In this study, we designed and synthesized a series of novel *β*-carboline derivatives modified at the *N^9^* position and linked at the *C^3^* position, and further investigated the antitumor activity and DNA-binding affinity in vitro. We expected to discover novel *β*-carboline derivatives with promising antitumor activity and DNA-binding affinity.

## 2. Results

### 2.1. Chemistry

**Scheme 1:**
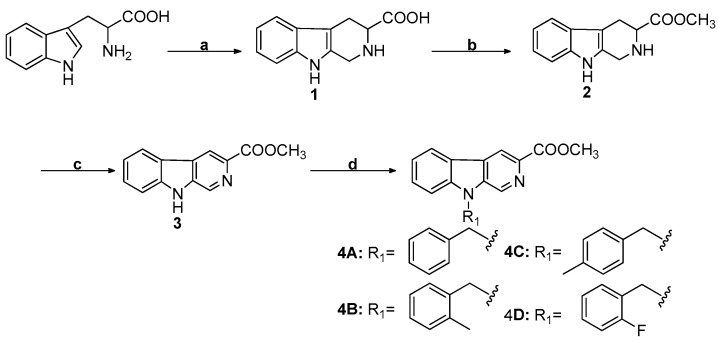
Synthesis of *N^9^*-substituted *β*-carboline-3-carboxylic acid methyl esters (**4A**, **4B**, **4C**, and **4D**). Reagents and conditions: (**a**) H^+^, HCHO, room temperature (r.t.); (**b**) SOCl_2_, CH_3_OH, reflux; (**c**) Pd/C, xylene, reflux; (**d**) DMF, NaH, r.t., R_1_-Br.

**Scheme 2:**
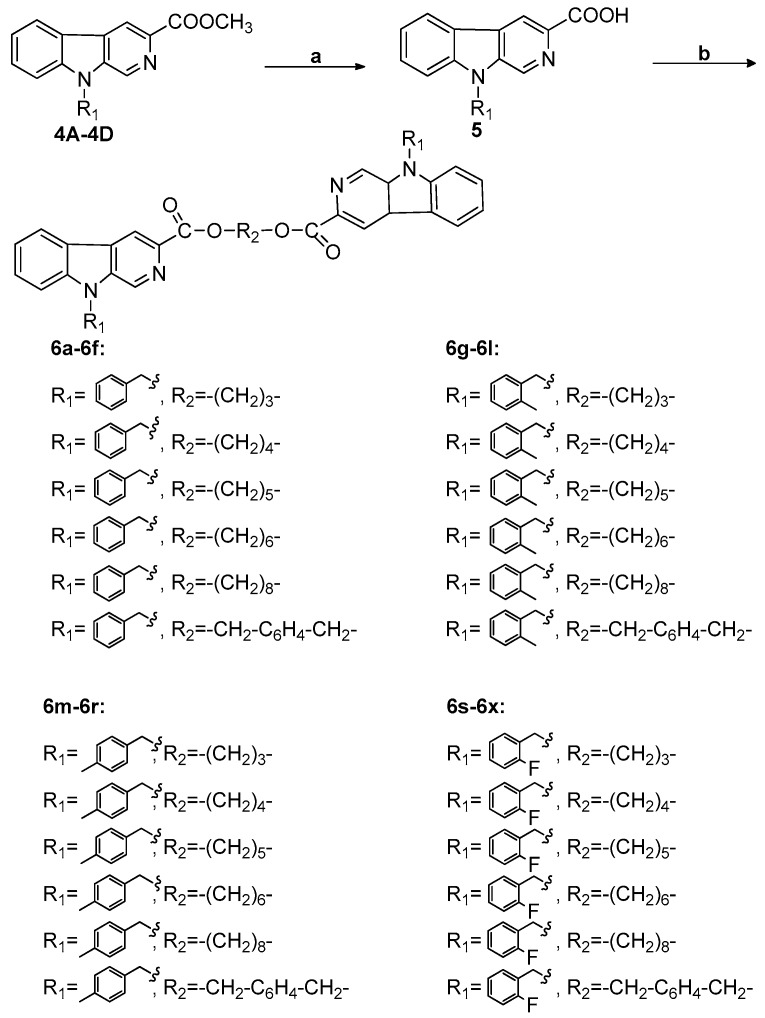
Synthesis of bivalent *β*-carboline-3-carboxylic acid derivatives (**6a-6f**, **6g-6l**, **6m-6r**, and **6s-6x**). Reagents and conditions: (**a**) THF/CH_3_OH, OH–, r.t.; (**b**) DMF, K_2_CO_3_, BrR_2_Br, heat.

Using L-tryptophan as a raw material, *N^9^* substituted *β*-carboline-3-carboxylic acid methyl esters (monomers, **4A**, **4B**, **4C**, and **4D**) were synthesized mainly through the Pictet–Spengler (P–S) reaction, esterification reaction, and 9-substitution reaction, as previously described [5,36]. The *N^9^* substituted *β*-carboline-3-carboxylic acid methyl esters prepared in the above steps were hydrolyzed and then reacted with dibromoalkane to obtain a series of bivalent *β*-carboline derivatives (dimers, **6a-6f**, **6g-6l**, **6m-6r**, and **6s-6x**) [37]. The synthetic routes and reaction conditions of *β*-carboline monomers (**4A**, **4B**, **4C**, and **4D**) and dimers (**6a-6f**, **6g-6l**, **6m-6r**, and **6s-6x**) are shown in Scheme 1 and Scheme 2, respectively. In this study, we synthesized 3 novel monomers and 21 novel dimers, and evaluated their antitumor activity.

### 2.2. *In Vitro* Cytotoxicity Assay

The antitumor activity of novel *β*-carboline derivatives modified at the *N^9^* position and linked at the *C^3^* position have been evaluated in vitro against A549, Hela, SGC-7901, SMMC-7721, MCF-7, and MRC5 cell lines using MTT (3-(4,5-dimethylthiazol-2-yl)-2,5-diphenyltetrazolium bromide) assay, with the results expressed as IC_50_ Values. Previous reports have evidenced that *β*-carbolines substituted with an alkoxycarbonyl or carboxyl at the *C^3^* position, and with a short alkyl or benzyl at the *N^9^* position, exhibited more significant antitumor activities against Hela cells [5]. In the present research (Table 1), for all the cell lines tested, only the *β*-carboline monomer substituted with *o*-fluorobenzyl (4D) at *N*^9^ position displayed hihest antitumor activity. For A549 cell line, among all the substituents at the *N^9^* position, it was found that the antitumor activity of *p*-methylbenzyl (**4C**, 28.06 μM) substituent was better than that of the *o*-methylbenzyl (**4B**, 39.20 μM) substituent. Next, the *o*-fluorobenzyl side chain with the lowest IC_50_ value (**4D**, 13.94 μM) was considered to be the most beneficial to increase antitumor activity at the *N^9^* position. But the antitumor activities of bivalent *β*-carboline derivatives (dimer, **6i**, 6.93 μM; **6u**, 5.61 μM) were apparently better than that of the corresponding monomer (**4B**, 39.20 μM; **4D**, 13.94 μM), indicating that the dimerization was an effective modification for improving the antitumor activity of *β*-carbolines. The IC_50_ values of bivalent *β*-carboline derivatives were relatively lower when the linkers were short in length and odd in carbon numbers (**6s**, 6.12 μM; **6u**, 5.61 μM). These results indicated that the introduction of *o*-fluorobenzyl at the *N^9^* position and the dimerization at the *C^3^* position could significantly increase the antitumor activity of *β*-carbolines, and they have better antitumor activity when the length of the linkers was four to six methylene units.

Moreover, most compounds exhibited low cytotoxicities against the MRC5 cell line. Because of the modest cytotoxicity of **6i** (6.93 μM) and **6u** (5.61 μM) against the A549 cell line, and in order to compare the antitumor mechanisms between monomers and dimers, **4B**, **6i**, **4D**, and **6u**, the A549 cells were selected to do the further investigation.

### 2.3. The Morphological Observation of Cell Apoptosis

**Figure 1:**
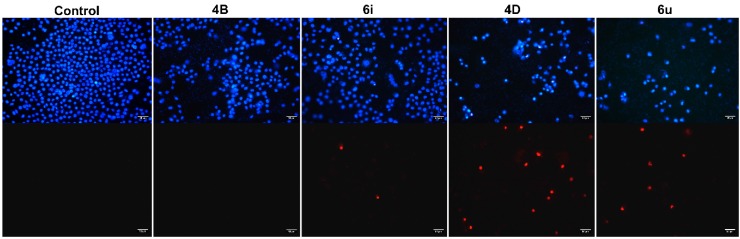
Morphological observation was performed by Hoechst 33342/propidium iodide (PI) dual staining. A549 cells were treated with **4B**, **6i**, **4D**, and **6u** at 8 μM for 48 h, and then stained with Hoechst 33342 and PI at 37 °C in dark conditions. The stained cells were observed under a fluorescence microscope (Leica DM6B, Leica, Nussloch, Germany). Hoechst 33342 was used to stain the living and apoptotic nuclei, and emitted blue fluorescence (**upper**). PI was used to stain the dead nuclei and emitted red fluorescence (**lower**).

Apoptotic morphology is one of the most intuitive and reliable ways to determine whether cells are apoptotic [38]. The typical features of apoptosis include membrane blebbing, cell contraction, chromatin condensation, and formation of apoptotic bodies [39]. To identify the typical features of apoptosis, we performed Hoechst 33342/propidium iodide (PI) dual staining assay. As shown in Figure 1, A549 cells in the control group were evenly dyed weak blue with clear edge. However, after incubation with **6i**, **4D**, and **6u** for 48 h, the cell density decreased significantly, and the cells were stained with strong blue fluorescence and strong red fluorescence, and shrinkage, condensation, or fragmentation was observed (Figure 1). These results indicated that the second series of dimer (**6i**) and the fourth series (containing fluorine atom) of monomer (**4D**) and dimer (**6u**) could induce cell apoptosis and further cell necrosis, while the second series of monomer (**4B**) could only induce cell apoptosis in a dose-dependent manner.

### 2.4. Cell Cycle Assay

**Figure 2:**
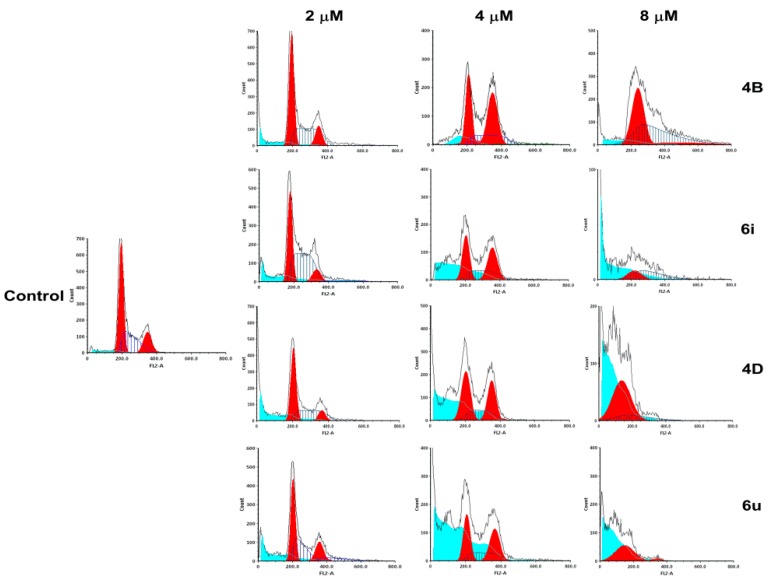
The cell cycle was detected by flow cytometry using PI single staining. A549 cells were treated with **4B**, **6i**, **4D**, and **6u** at 2, 4, and 8 μM for 48 h, and then stained with PI for 30 min at 37 °C. The proportions of cells containing different DNA content were analyzed by FCS Express 5.0 (De Novo Software, Thornhill, Canada).

In order to determine the cell cycle arrest induced by *β*-carbolines, we performed propidium iodide (PI) staining using flow cytometry. As shown in Figure 2 and Table 2, when the concentration of **4B**, **6i**, **4D**, and **6u** was 4 μM, the percentages of cells in the G2/M phase increased to 46.96%, 42.40%, 35.46%, and 40.34% (control group 12.25%), respectively, indicating that **4B**, **6i**, **4D**, and **6u** could induce cell cycle arrest at the G2/M phase in A549 cells. At 8 μM, the percentages of cells in the S phase of **4B** and **6i** increased to 45.81% and 53.30% (control group 27.6%), respectively, indicating that **4B** and **6i** could exert antitumor activity by causing S and G2/M phase arrest. At 8 μM of **4D** and **6u**, the percentages of cells in the G1 phase increased to 88.01% and 81.62% (control group 60.15%), respectively, indicating that **they** could exert antitumor activity by causing G1 and G2/M phase arrest. Moreover, at 8 μM, the percentages of cells in sub-G1 by **4B**, **6i**, **4D**, and **6u** increased to 4.15%, 21.32%, 46.29%, and 44.92% (control group 1.78%), respectively, indicating that they could also induce apoptosis.

### 2.5. Cell Apoptosis Assay

**Figure 3:**
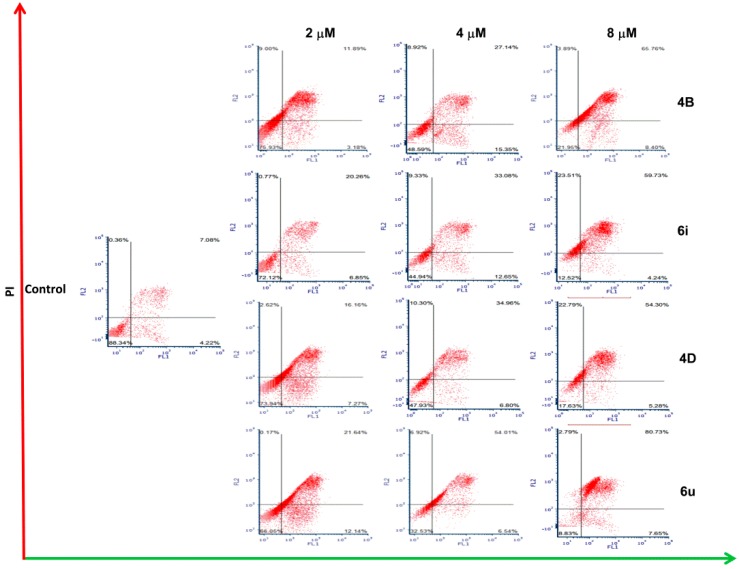
The cell apoptosis was detected by flow cytometry using Annexin V-FITC/PI dual staining. A549 cells were treated with **4B**, **6i**, **4D**, and **6u** at 2, 4, and 8 μM for 48 h, and then stained with Annexin V-FITC and PI for 15 min at 37 °C. The proportions of the living cells, the early apoptosis cells, and the late apoptotic cells were analyzed by FCS Express 5.0.

To better understand the apoptosis induced by *β*-carboline derivatives, we further performed Annexin V-FITC/PI dual staining assay using flow cytometry. The transfer of phosphatidylserine from the cell inner membrane to the cell outer membrane is considered as a potential marker of cell apoptosis. Moreover, Annexin V is a cellular protein which selectively binds phosphatidylserine [40,41]. As shown in Figure 3 and Table 3, after treatment with 8 μM of **4B**, **6i**, **4D**, and **6u** for 48 h, the percentages of early apoptosis were 8.40%, 4.24%, 5.28%, and 7.65%, respectively. And the percentages of late apoptosis or necrosis were 69.65%, 83.24%, 77.09%, and 83.52%, respectively, indicating that **4B**, **6i**, **4D**, and **6u** could induce significant apoptosis compared with the control group (early apoptosis: 4.22%; late apoptosis or necrosis: 7.44%). To sum up, the results of cell cycle and apoptosis experiments demonstrated that **4B**, **6i**, **4D**, and **6u** could exert antitumor activity by inducing cell cycle arrest at the S or G2/M phase and significant cell apoptosis.

### 2.6. The Detection of Apoptosis-Related Protein (Cytochrome C, bcl-2)

**Figure 4:**
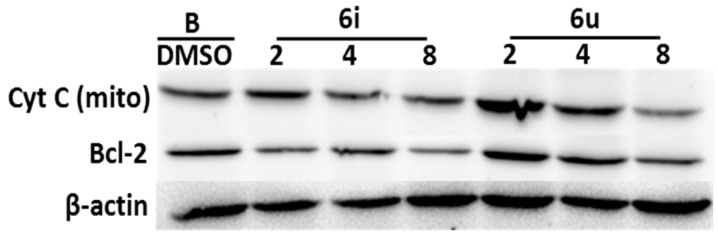
Western blot analysis of cytochrome C and *bcl-2* proteins. A549 cells were treated with with **6i** and **6u** at 2, 4, and 8 μM for 48 h. *β*-actin was used as an internal control.

A large number of studies have found that the release of cytochrome *C* (Cyt *C*) from mitochondria to cytoplasm, and the decrease in expression of *bcl-2* protein, are important features of apoptosis [42,43]. As shown in Figure 4, the levels of Cyt C (in mitochondria) and the expression of *bcl-2* protein decreased in a dose-dependent manner after treatment with **6i** and **6u**. The results demonstrated that **6i** and **6u** could induce mitochondria-mediated apoptosis.

### 2.7. DNA Binding Studies

The binding mode and binding strength of *β*-carboline derivatives (**4B**, **6i**, **4D**, and **6u**) to DNA were studied through UV-visible spectral, thermal denaturation, and molecular docking studies.

#### 2.7.1. UV-Visible Spectral Study

**Figure 5:**
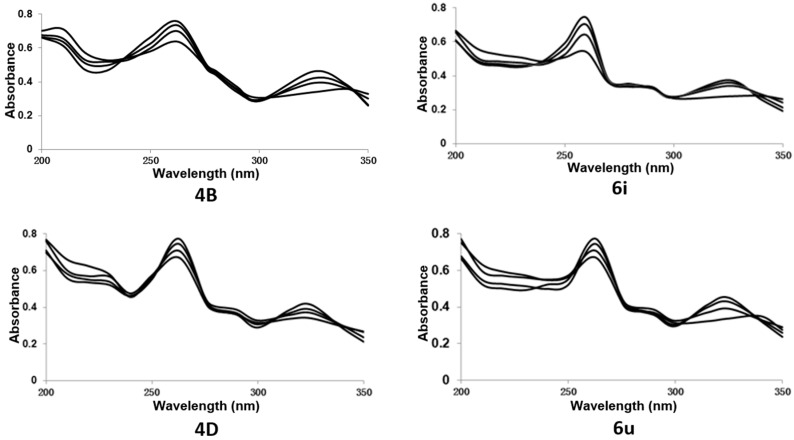
UV-visible spectroscopy study of *β*-carbolines with calf thymus (CT) DNA. UV-visible absorption titrations were done by adding 200 nM CT DNA solution each time to the quartz cuvette containing 10 μM **4B**, **6i**, **4D**, and **6u**. The absorption spectra were recorded from 200 nm to 350 nm.

In order to reveal the binding mode of *β*-carboline derivatives to DNA, the UV absorption spectra of the interactions of *β*-carbolines (**4B**, **6i**, **4D**, and **6u**) with calf thymus DNA (CT DNA) were investigated (Figure 5). The absorbance at 325 nm of **4B**, **6i**, **4D**, and **6u** was gradually decreased with red shift (**4B**-15 nm, **6i**-18 nm, and **6u**-20 nm), indicating that **4B**, **6i**, and **6u** could bind to DNA by intercalation. Among them, the intercalation ability of **4D** was very weak (may be a reasonable experimental error), and it may interact with DNA through other intermolecular forces.

#### 2.7.2. Thermal Denaturation Study

**Figure 6:**
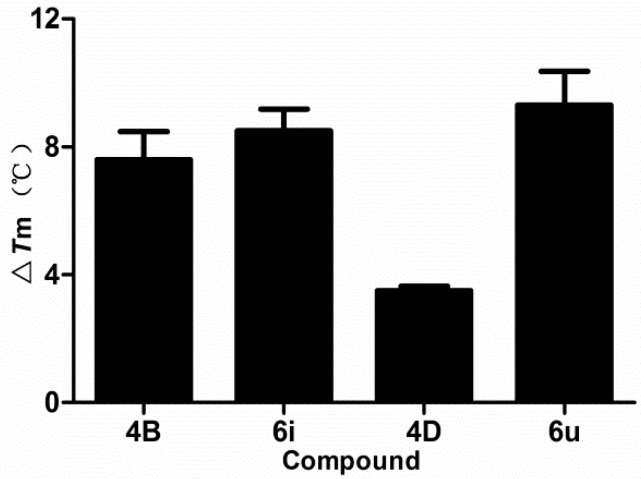
Thermal denaturation study of the complexes of *β*-carbolines (**4B**, **6i**, **4D**, and **6u**) and CT-DNA. Melting temperatures were measured in PBS-EDTA buffer (1 mM Na_2_HPO_4_, 0.1 mM EDTA, PH 7.4) with a *β*-carboline (**4B**, **6i**, **4D**, and **6u**)/DNA ratio of 0.5. Δ*T*_m_ = (Δ*T*_m_^**4B**,**6i**,**4D**, or **6u** + DNA^ − Δ*T*_m_^DNA^).

The binding affinity of *β*-carboline derivatives (**4B**, **6i**, **4D**, and **6u**) to DNA were investigated through thermal denaturation experiments. Small molecules interact with DNA through the intercalation mode, which makes the DNA double helix more stable, thereby increasing the melting temperature (*T*_m_) [44]. Therefore, the thermal denaturation experiment provides a simple and effective method for detecting DNA-binding affinity. As shown in Figure 6, the Δ*T*_m_ values of **4B**, **6i**, **4D**, and **6u** were 7.5, 8.5, 3.5, and 9.0 °C, respectively, indicating that they could bind to DNA by intercalation, and the binding strength of **4B**, **6i**, and **6u** was greater than that of **4D**. Thus, thermal denaturation results indicated that **4B**, **6i**, **4D**, and **6u** could exhibit significant DNA-binding affinity.

#### 2.7.3. Molecular Docking Study

**Figure 7:**
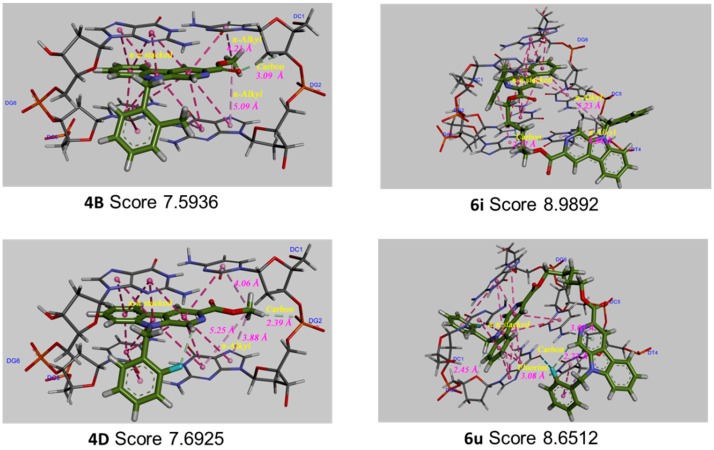
Three-dimensional conformations of the complexes of *β*-carbolines (**4B**, **6i**, **4D**, and **6u**) docked with DNA.

In order to further predict the binding mode of *β*-carboline to DNA, the Surflex-Dock program in the Sybyl-X 2.0 package (Tripos, Princeton, NJ, USA) was used to perform molecular docking studies between *β*-carboline derivatives and DNA, to explore possible mechanisms of antitumor activity. The structures and docking scores of the DNA-*β*-carboline complex are shown in Figure 7. The total scores of **4B**, **6i**, **4D**, and **6u** were 7.5936, 8.9892, 7.6925, and 8.6512, respectively, indicating that **4B**, **6i**, **4D**, and **6u** could well insert into the DNA. Moreover, the scores of dimers (**6i** and **6u**) were higher than that of monomers (**4B** and **4D**), indicating that the dimers have stronger DNA-binding ability than the corresponding monomers, thereby exerting better antitumor activity. In addition, the planar structure of *β*-carbolines (**4B**, **6i**, and **4D**) could interact with DNA through π-π stacking, the carbon-hydrogen bond (non-classical hydrogen bond), and the π-alkyl bond (hydrophobic force). The presence of the fluorine bond between DNA and **6u** might support its higher antitumor activity.

## 3. Discussion

*β*-carboline alkaloids are a class of natural and synthetic indole alkaloids with a wide range of pharmacological activities, including antitumor, antiviral, antiparasitic, and antibacterial activities [5,6]. Particularly, previous studies have shown that *β*-carboline alkaloids exerted antitumor activity through insertion into DNA [10,11,12], inducing apoptosis [13,45], inhibiting CDKs [16], and topoisomerase I and II activity [13,15]. The substitution at the *C^3^* and *N^9^* positions of *β*-carbolines could significantly increase their DNA insertion ability [7]. Moreover, the existing studies have found that dimerization of small molecules could significantly increase [26].

DNA binding affinity [31,32]. Therefore, designing and synthesizing DNA-targeted *β*-carboline derivatives is an important way to find potentially effective antitumor drugs. Here, we synthesized a series of bivalent *β*-carboline derivatives modified at the *N^9^* position and dimerized at the *C^3^* position, and evaluated their antitumor activity and DNA binding affinity in vitro. The IC_50_ values of **4B**, **6i**, **4D**, and **6u** against A549 were 39.20, 6.39, 13.94, and 5.61 μM, respectively.

Previous studies have shown that the antitumor activity of *β*-carboline could be significantly improved with benzyl substituted at the *N^9^* position and dimerized at the *C^3^* position [5,35]. In this study, the monomers exhibited good-to-strong antitumor activity with the tendency of *o*-fluorobenzyl > *p*-methylbenzyl > *o*-methylbenzyl > benzyl. Furthermore, for the dimers of *o*-methylbenzyl substituted at the *N^9^* position, most dimers were generally more active than the corresponding monomers, following the tendency of 5 > 4 > 6 > 3 > 8 methylene units. For the dimers of *o*-fluorobenzyl substituted at the *N^9^* position, most dimers were generally more active than the corresponding monomers, following the tendency of 5 > 3 > 6 > 4 > 8 methylene units. These results indicated that *N^9^*-benzyl substitution and *C^3^*-dimerizition with 5–6 methylene units were favorable for increasing antitumor activity, which was consistent with the results of previous studies.

Previous studies have shown that cell cycle arrest and cell apoptosis are the main mechanisms used by *β*-carboline derivatives as antitumor drugs [4,46]. Our previous studies have shown that the introduction of substituents (a methyl or benzyl group) at the *N^9^* position and the dimerization of *β*-carbolines could significantly increase the antitumor activity, and could cause cell cycle arrest at the S or G2/M phase and induce apoptosis in a dose-dependent manner [36,37]. In the present study, we found that the cell cycle arrested at the S and G2/M phases with sub G1 peaks, and induced apoptosis after treatment with **4B**, **6i**, **4D**, and **6u**. Furthermore, the reduction of cytochrome *C* (Cyt *C*) in mitochondria, and the decrease in expression of *bcl-2* protein, are the typical features of mitochondria-mediated apoptosis [47]. Additionally, the results of western blot showed that the levels of Cyt *C* (in mitochondria) and the expression of *bcl-2* protein decreased in a dose-dependent manner after incubation with **6i** and **6u**. Taken together, these results revealed that **6i** and **6u** could induce cell apoptosis through a mitochondria-mediated pathway.

To date, DNA intercalators have been effective antitumor drugs in clinical application. After the drugs are inserted into DNA, they can cause conformational changes of the double helix, which disrupts DNA replication, transcription, and repair [23]. The antitumor activity of a large number of natural and synthetic *β*-carboline derivatives that could interact with DNA by intercalation have been discovered and evaluated. Furthermore, the interactions between these derivatives (**4B**, **6i**, **4D**, and **6u**) and DNA were investigated by UV-visible spectroscopy, thermal denaturation, and molecular modeling studies. The absorbance of **4B**, **6i**, **4D**, and **6u** decreased with red shift, and the Δ*T*_m_ values of **4B**, **6i**, **4D**, and **6u** were 7.5, 8.5, 3.5, and 9.0 °C, respectively, indicating that **4B**, **6i**, and **6u** could stably bind to DNA by intercalation, which is in agreement with the results of molecular docking studies.

In conclusion, a series of novel bivalent *β*-carboline-3-carboxylic acid derivatives were synthesized, and antitumor activity and DNA-binding affinity were preliminarily evaluated in vitro. The SARs studies revealed that the introduction of appropriate substituents at the *N^9^* position and dimerization at the *C^3^* position of *β*-carbolines could significantly increase the antitumor activity against A549, Hela, SGC-7901, SMMC-7721, and MCF-7 cell lines. Furthermore, **4B**, **6i**, **4D**, and **6u** could further induce tumor cell cycle arrest and apoptosis, and could interact with DNA through intercalation. In summary, further studies of **4B**, **6i**, **4D**, and **6u** could be performed to develop potential DNA-targeted agents in clinical applications.

## 4. Experimental Section

### 4.1. Materials and Methods

All chemicals and solvents used in the synthesis were purchased from suppliers. They were dried and purified when needed. Melting points were determined on a capillary melting apparatus and were uncorrected (SGW X-4, Shanghai Shenguang Instrument Co., Ltd., Shanghai, China). The ^1^H-NMR and ^13^C-NMR spectra were recorded on a Bruker Avance III at 500 MHz using tetra methyl silane (TMS) as the internal standard. The column chromatography and analytical thin layer chromatography (TLC) were performed with silica gel (100–200 mesh) and silica gel GF_254_ (Qingdao Marine Chemical Company, Qingdao, China).

All cell lines (A549, SGC-7901, Hela, SMMC-7721, and MCF-7) were purchased from the American Type Culture Collection (Manassas, VA, USA), and were cultured in Dulbecco’s modified Eagle’s medium (DMEM, HycloneLaboratories Inc., Logan, Utah, USA) supplemented with 10% fetal bovine serum (FBS, Gibco&Invitrogen, Carlsbad, CA, USA) at 37 °C and 5% CO_2_.

### 4.2. Chemistry

#### 4.2.1. General Synthesis Procedure for *N*^9^ Substituted *β*-Carboline-3-carboxylic Acid Methyl Esters (**4A**, **4B**, **4C**, and **4D**)

Using L-tryptophan as the raw material, *β*-carboline monomers (**4A**, **4B**, **4C**, and **4D**) were synthesized through the Pictet–Spengler reaction (P–S reaction), esterification reaction, oxidation reaction, and *N*^9^ substitution reaction, as previously described [5,26,36]. Reaction routes and conditions are shown in Scheme 1.

#### 4.2.2. General Reaction of Synthesis of Bivalent *β*-Carboline Derivatives (**6a-6f**, **6g-6l**, **6m-6r**, and **6s-6x**)

The *N*^9^ substituted *β*-carboline-3-carboxylic acid methyl esters (**4A**, **4B**, **4C**, and **4D**) prepared in the above steps were hydrolyzed and reacted with dibromoalkane to obtain a series of bivalent *β*-carboline-3-carboxylic acid derivatives (**6a-6f**, **6g-6l**, **6m-6r**, and **6s-6x**), as previously described [34,35,37,48]. Reaction routes and conditions are shown in Scheme 2.

### 4.3. MTT Assay

The cytotoxic activities of *β*-carboline-3-carboxylic acid derivatives were investigated by the MTT method, as previously described [49]. Cells (1 × 10^4^ cells per well) seeded into 96-well plates (JET, Guangzhou, China) were incubated with DMEM containing various concentrations (2.5, 5, 10, 20, 40, and 80 μM) of *β*-carbolines (**4A**, **4B**, **4C**, **4D**, **6a-6f**, **6g-6l**, **6m-6r**, and **6s-6x**) for 48 h at 37 °C and 5% CO_2_. Then, 10 µL of 5 mg/mL MTT reagent dissolved in PBS (Phosphate buffered saline) was added per well, and plates were incubated at 37 °C for 4 h. The medium was carefully removed and 100 µL dimethyl sulfoxide (DMSO) was added to dissolve the formazan crystals. The absorbance was measured at 490 nm with a microplate reader (ThermoFisher Scientific Inc., Waltham, MA, USA). The *β*-carbolines were dissolved in DMSO as stock solutions (10 mM) at −20 °C, and diluted in the final experimental solution using DMEM before use. A549 (lung carcinoma), SGC-7901 (gastric carcinoma), Hela (cervical carcinoma), SMMC-7721 (liver carcinoma), and MCF-7 (breast carcinoma) cell lines were used for the antitumor activity screening. The IC_50_ values were the mean values of three independent experiments.

### 4.4. Hoechst 33342/PI Dual Staining Assay

The morphological observation of apoptosis was performed by Hoechst 33342 and propidium iodide (PI) dual staining according to the manufacturer’s instructions. A549 cells (2 × 10^4^ cells per well) seeded into 24-well plates (JET, Guangzhou, China) were incubated with DMEM containing 4 μM of *β*-carbolines (**4B**, **6i**, **4D**, and **6u**) for 48 h at 37 °C and 5% CO_2_. Then, the cells were stained by Hoechst 33342 and PI at 37 °C for 30 min in dark conditions. Finally, cells were observed and photographed with a fluorescence microscope (LeicaDM6B, Leica, Nussloch, Germany).

### 4.5. Cell Cycle Assay

The cell cycle was determined by PI single staining. A549 cells (1 × 10^6^ cells per well) seeded into 6-well plates (JET, Guangzhou, China) were incubated with DMEM containing various concentrations (2, 4, and 8 μM) of *β*-carbolines (**4B**, **6i**, **4D**, and **6u**) for 48 h at 37 °C and 5% CO_2_. The cells were harvested, washed with pre-cooled PBS (4 °C), and fixed with pre-cooled 90% ethanol (4 °C). Then, the cells were incubated with 20 μL RNAse (30 μg/mL) and 20 μL PI (50 μg/mL) (Sigma Aldrich, Saint Louis, MO, USA) for 30 min at 37 °C. The samples were analyzed using flow cytometry (BD FACSCalibur, San Jose, CA, USA).

### 4.6. Cell Apoptosis Assay

The cell apoptosis was evaluated using Annexin V-FITC/PI dual staining (Beyotime, China). A549 cells (1 × 10^6^ cells per well) seeded into 6-well plates (JET, Guangzhou, China) were incubated with DMEM containing various concentrations (2, 4, and 8 μM) of *β*-carbolines (**4B**, **6i**, **4D**, and **6u**) for 48 h at 37 °C and 5% CO_2_. The cells in the supernatant and adherent were collected and washed with PBS, and then the cells were stained with Annexin V-FITC and PI for 20 min at r.t. The samples were analyzed using flow cytometry (Becton Dickinson, San Jose, CA, USA).

### 4.7. Western Blot Assay

The level of cytochrome C (Cyt C) in mitochondria, and the expression of *bcl-2* protein, were evaluated by western blot. A549 cells (1 × 10^6^ cells per well) seeded into 6-well plates (JET, Guangzhou, China) were incubated with DMEM containing various concentrations (2, 4, and 8 μM) of *β*-carbolines (**6i** and **6u**) for 48 h at 37 °C and 5% CO_2_. Then, the cells were lysed with RIPA lysis buffer for 30 min on ice (containing 1% PMSF), centrifuged at 12,000× *g* for 5 min at 4 °C, and then the supernatants were transferred to 1.5 mL EP tubes. Proteins were separated by sodium dodecyl sulfate-polyacrylamide gel electrophoresis (SDS-PAGE) and blotted on polyvinylidene difluoride (PVDF) membranes (Millipore, Burlington, MA, USA), as previously described [50]. The membranes were blocked for 2 h at r.t. with 5% non-fat milk in TBST buffer (20 mM Tris-HCl,150 mM NaCl and 0.05% Tween-20, pH7.4), and then incubated with the mouse anti-*bcl-2* monoclonal antibody (Sigma, Saint Louis, MO, USA), anti-cytochrome C monoclonal antibody (Beyotime, Haimen, China), and anti-*β*-actin antibody (1:4000 dilution; Abcam, Cambridge, MA, USA) overnight at 4 °C, and finally incubated with the peroxidase conjugated goat anti-mouse IgG (Jackson ImmunoResearch Laboratories, West Grove, PA, USA) at 37 °C for 1 h. Immunostained proteins were visualized using ECL reagent according to the manufacturer’s instructions (Pierce, Rockford IL, CA, USA). *β*-actin was used as an internal control.

### 4.8. UV-Visible Spectral Study

UV-visible spectroscopy was carried out to determine the binding mode of *β*-carboline derivatives to DNA (CT DNA) using UV-2500 spectrophotometer (Shimadzu, Kyoto, Japan) at 25 °C. Stock solutions of 10 mM *β*-carboline derivatives were dissolved in DMSO, and 10 μM CT DNA was prepared in 100 mM KBPES buffer (30 mM Potassium Phosphate with 100 mM KCl, pH 7.0) [13]. UV-visible absorption titrations were performed by adding 200 nM CT DNA solution to the quartz cuvette containing approximately 10 μM derivative solutions. UV-Visible absorption spectra were recorded from 200 nm to 350 nm.

### 4.9. Thermal Denaturation Study

The DNA-binding modes of the *β*-carboline derivatives (**4B**, **6i**, **4D**, and **6u**) with CT DNA were examined by DNA thermal denaturation experiments, as previously described [10]. The solutions containing complexes of CT DNA and **4B**, **6i**, **4D**, or **6u** were heated using a thermostatic water bath, and the absorbance was measured in 1 °C steps from 30 °C to 95 °C at 260 nm (A_t_) by a UV-Vis spectrophotometer (A is the absorbance of the solution at 260 nm at different temperatures, t is the temperature). The experiments were performed in PE buffer (pH = 7.4) with a *β*-carboline/DNA ratio of 0.5. The Δ*T*_m_ value of DNA alone is 56.8 °C. Δ*T*_m_ = Δ*T*_m_^(DNA + **4B**, **6i**, **4D**, or **6u**)^ − Δ*T*_m_^(DNA alone)^.

### 4.10. Molecular Modeling Study

Molecular docking software (Sybyl-X 2.0 Tripos, Princeton, NJ, USA) and Discovery Studio 2017 client (BIOVIA, San Diego, CA, USA) was used to investigate the interaction of **4B**, **6i**, **4D**, and **6u** with DNA. The DNA was retrieved from the Protein Data Bank (PDB entry code: 1Z3F). The crystal structure of the DNA molecule was prepared by adding all the hydrogen atoms, and the charge was added using the AMBER7 FF99 method. The structures of **4B**, **6i**, **4D**, and **6u** were drawn in the Sybyl-X 2.0 package (Tripos, Princeton, NJ, USA). Hydrogen atoms were added, and then the energy was optimized using the Tripos force field and the Gasteiger–Hückel method. The Surflex-Dock scoring function is a weighted sum of non-linear functions based on the binding affinities of DNA-ligand complexes and their crystallographic structures.

## Figures and Tables

**Table 1:** The IC_50_ values (μM) of *β*-carboline monomers (**4A**, **4B**, **4C**, and **4D**) and bivalent *β*-carboline derivatives (**6a-6f**, **6g-6l**, **6m-6r**, and **6s-6x**) against A549, SGC-7901, Hela, SMMC-7721, MCF-7, and MRC5 cell lines.

Compounds	Substituents		IC_50_ (μM) Mean ± SD ^a^					
	R_1_	R_2_	A549 ^b^	SGC-7901 ^b^	Hela ^b^	SMMC-7721 ^b^	MCF-7 ^b^	MRC5
**4A**	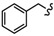	-	>80	>80	>80	>80	>80	35.60 ± 0.30
**4B**	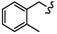	-	39.20 ± 1.18	42.31 ± 2.13	31.28 ± 2.33	>80	>80	51.78 ± 0.10
**4C**	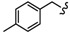	-	28.06 ± 0.13	47.98 ± 0.09	42.55 ± 0.98	>80	>80	35.35 ± 0.30
**4D**	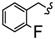	-	13.94 ± 0.07	17.84 ± 0.66	26.36 ± 1.33	35.61 ± 1.33	53.92 ± 2.02	35.38 ± 0.01
**6a**	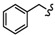	-(CH_2_)_3_-	24.27 ± 0.12	17.76 ± 0.17	17.89 ± 0.03	25.73 ± 0.32	73.45 ± 0.88	32.57 ± 0.10
**6b**	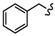	-(CH_2_)_4_-	12.26 ± 0.04	15.48 ± 0.32	18.22 ± 0.52	66.53 ± 0.31	70.88 ± 2.33	64.44 ± 0.09
**6c**	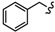	-(CH_2_)_5_-	17.65 ± 0.09	18.72 ± 0.07	18.15 ± 0.03	55.15 ± 0.03	76.85 ± 3.01	39.34 ± 0.11
**6d**	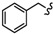	-(CH_2_)_6_-	40.03 ± 0.15	58.41 ± 0.72	32.36 ± 0.02	33.49 ± 0.01	75.47 ± 2.32	66.99 ± 0.10
**6e**	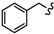	-(CH_2_)_8_-	50.32 ± 3.15	69.97 ± 0.13	53.97 ± 0.56	75.36 ± 0.89	>80	>100
**6f**	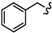	-CH_2_-C_6_H_4_-CH_2_-	29.04 ± 0.06	51.24 ± 3.22	66.3 ± 1.02	57.35 ± 1.13	>80	>100
**6g**	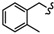	-(CH_2_)_3_-	18.33 ± 0.28	29.42 ± 1.03	17.99 ± 0.15	73.49 ± 8.06	53.14 ± 2.28	32.61 ± 0.10
**6h**	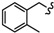	-(CH_2_)_4_-	8.09 ± 0.25	17.75 ± 0.93	45.75 ± 0.55	24.71 ± 0.5	76.73 ± 5.0	50.78 ± 0.09
**6i**	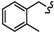	-(CH_2_)_5_-	6.93 ± 0.33	7.18 ± 0.01	9.88 ± 0.04	14.22 ± 0.28	59.29 ± 5.86	37.99 ± 0.10
**6j**	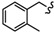	-(CH_2_)_6_-	12.18 ± 0.06	26.26 ± 1.03	18.05 ± 1.03	16.68 ± 0.03	64.49 ± 3.31	41.0 ± 0.07
**6k**	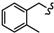	-(CH_2_)_8_-	37.36 ± 2.08	35.00 ± 2.09	36.69 ± 2.02	33.93 ± 0.52	44.93 ± 2.99	65.85 ± 0.07
**6l**	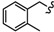	-CH_2_-C_6_H_4_-CH_2_-	30.13 ± 0.37	39.26 ± 1.22	59.68 ± 1.52	69.66 ± 0.30	70.76 ± 2.02	37.66 ± 0.13
**6m**	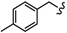	-(CH_2_)_3_-	19.84 ± 0.04	18.41 ± 0.31	17.95 ± 0.02	33.32 ± 0.03	28.43 ± 0.28	49.05 ± 0.12
**6n**	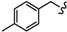	-(CH_2_)_4_-	13.36 ± 0.35	37.79 ± 1.02	27.96 ± 0.99	42.72 ± 2.18	49.06 ± 2.23	26.39 ± 0.01
**6o**	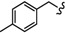	-(CH_2_)_5_-	11.23 ± 0.61	17.18 ± 0.03	18.76 ± 0.46	36.62 ± 0.12	49.37 ± 1.65	31.28±0.07
**6p**	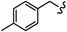	-(CH_2_)_6_-	19.28 ± 0.02	17.84 ± 0.22	15.66 ± 0.33	26.75 ± 0.51	55.04 ± 2.22	47.60 ± 0.02
**6q**	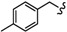	-(CH_2_)_8_-	23.65 ± 0.05	35.78 ± 0.82	45.21 ± 1.66	29.82 ± 0.93	43.05 ± 2.01	42.06 ± 0.09
**6r**	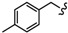	-CH_2_-C_6_H_4_-CH_2_-	46.61 ± 0.03	65.93 ± 1.18	58.25 ± 2.98	>80	68.26 ± 1.25	56.11 ± 0.01
**6s**	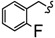	-(CH_2_)_3_-	6.12 ± 0.03	8.06 ± 0.08	13.77 ± 0.11	14.71 ± 0.08	25.20 ± 1.12	22.82 ± 0.02
**6t**	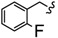	-(CH_2_)_4_-	12.39 ± 0.59	9.01 ± 0.19	15.20 ± 0.32	18.26 ± 0.18	25.69 ± 1.21	>100
**6u**	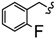	-(CH_2_)_5_-	5.61 ± 0.04	5.86 ± 0.08	14.63 ± 0.36	16.68 ± 0.11	36.03 ± 2.99	39.89 ± 0.05
**6v**	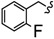	-(CH_2_)_6_-	7.95 ± 0.06	8.60 ± 0.13	22.85 ± 1.36	24.65 ± 0.06	42.41 ± 2.08	67.75 ± 0.11
**6w**	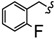	-(CH_2_)_8_-	16.54 ± 0.33	29.98 ± 0.07	31.34 ± 1.88	44.57 ± 0.02	76.32 ± 3.36	60.78 ± 0.16
**6x**	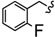	-CH_2_-C_6_H_4_-CH_2_-	34.27 ± 0.05	45.00 ± 0.16	39.61 ± 2.59	>80	>80	63.28 ± 0.10
**5-FU ^c^**	C_4_H_3_FN_2_O_2_		15.39 ± 0.09	>80	>80	52.32 ± 2.06	57.69 ± 1.39	16.33 ± 0.30

^a^ IC_50_ was the concentration when cell viability was reduced by 50% after treatment with *β*-carbolines for 48 h, and all data were the mean values of three independent experiments. ^b^ A549: lung carcinoma, SGC-7901: gastric carcinoma, Hela: cervical carcinoma, SMMC-7721: liver carcinoma, MCF-7: breast carcinoma, MRC5: normal lung cell. ^c^ 5-FU (5-fluorouracil, which could insert into DNA, interfering with DNA synthesis) was used as a positive control.

**Table 2:** The percentage of cell cycles of A549 cells at indicated compound concentrations (**4B**, **6i**, **4D**, and **6u** at 2, 4, and 8 μM).

Compounds	Concentration (μM)	Percentage of Cell Cycles (%)			
		SubG1	G1	S	G2/M
**4B**	2	6.56	59.38	25.16	15.46
	4	10.49	43.06	9.98	46.96
	8	4.15	52.09	45.81	2.1
**6i**	2	6.86	57.38	32.08	10.54
	4	26.33	45.19	12.41	42.40
	8	21.32	46.64	53.30	0.06
**4D**	2	6.41	56.89	24.17	18.94
	4	39.03	47.21	17.33	35.46
	8	46.29	88.01	11.69	0.3
**6u**	2	6.84	56.24	23.79	19.97
	4	46.25	47.06	12.60	40.34
	8	44.92	81.62	0.08	18.30
**Control**	1% DMSO	1.78	60.15	27.6	12.25

**Table 3:** The percentage of cell apoptosis of A549 cells at indicated compound concentrations (**4B**, **6i**, **4D**, and **6u** at 2, 4, and 8 μM).

Comp.	Concentration (μM)	Percentage of Cell Apoptosis (%)		
		Normal Living Cell	Early Apoptosis	Late Apoptosis and Necrosis
**4B**	2	75.93	3.18	20.89
	4	48.59	15.35	36.06
	8	21.95	8.40	69.65
**6i**	2	72.12	6.85	21.03
	4	44.94	12.65	42.41
	8	12.52	4.24	83.24
**4D**	2	73.94	7.27	18.78
	4	47.93	6.80	45.26
	8	17.63	5.28	77.09
**6u**	2	66.05	12.14	21.81
	4	32.53	6.54	60.93
	8	8.83	7.65	83.52
**Control**	1% DMSO	88.34	4.22	7.44

## Supplementary Materials

Supplementary materials can be found at http://www.mdpi.com/1422-0067/19/10/3179/s1.
