# Supplementary material for: Synthesis and In Vitro Antitumor Activity of Novel Bivalent β-Carboline-3-carboxylic Acid Derivatives with DNA as a Potential Target
Source: Int J Mol Sci. 2018 Oct 15;19(10):3179. doi: 10.3390/ijms19103179 (PMC6214108; doi:10.3390/ijms19103179)
Supplement: Supplementary file 1 [file ijms-19-03179-s001.pdf]

Supplementary Materials

Synthesis and *in vitro* antitumor activity of novel bivalent β-carboline derivatives with DNA as a potential target

Hongling Gu[1], Na Li[1], Jiangkun Dai[1], Yaxi Xi[1], Shijun Wang[1], Junru Wang[1,2,*]

[1] Shaanxi Key Laboratory of Natural Products & Chemical Biology, College of Chemistry and Pharmacy, Northwest A&F University, No. 22 Xinong Road, Yangling, Shaanxi 712100, China; honglingguw@163.com; lnuk@nwsuaf.edu.cn; daijkun@hotmail.com; mysteryxi@163.com; 444795415@nwafu.edu.cn;

[2] State key Laboratory of Drug Research, Shanghai Institute of Materia Medica, Chinese Academy of Sciences, 555 Zu Chong Zhi Road, Zhangjiang Hi-Tech Park, 201203, Shanghai, China.

* Corresponding authors:

Junru Wang, Ph.D

College of Chemistry and Pharmacy, Northwest A&F University, No. 22 Xinong Road, Yangling, Shaanxi 712100, China.

State key Laboratory of Drug Research, Shanghai Institute of Materia Medica, Chinese Academy of Sciences, 555 Zu Chong Zhi Road, Zhangjiang Hi-Tech Park, 201203, Shanghai, China.

Email addresses: wangjunru@nwafu.edu.cn

S.1. General reaction of synthesis of β-carboline monomers (**4A**, **4B**, **4C**, and **4D**)

L-tryptophan (10 g, 49 mmol) was dissolved in ultrapure water (450 mL). And the sulfuric acid (0.1 mol/L) was added dropwise with stirring until the solution became clear. And then 11 mL 37% formaldehyde solution was added and the mixture was reacted at room temperature for about 5 h. Then, the pH of the reaction solution was adjusted to about 6 with the saturated $NaHCO_3$ solution to obtain a white precipitate, and the precipitate was collected by negative-pressure filtration and washed well with water and then dried to obtain the target product **1** (1, 2, 3, 4-tetrahydro-β-carboline-3-carboxylic acid). The yield was about 70% (7.23 g).

The target product **1** (1, 2, 3, 4-tetrahydro-β-carboline-3-carboxylic acid) (500 mg, 2.3 mmol) was dissolved in dried ethanol (20 mL), and thionyl chloride (1 mL) was added under an ice bath. The mixture was refluxed for 15 h and then the pH was adjusted to 8. After extraction with ethyl acetate and purification by column chromatography (PE: EA = 1: 1), the target product **2** (1, 2, 3, 4-tetrahydro-β-carboline-3-carboxylic acid methyl ester) was obtained. The yield was about 63.7% (339 mg).

The target product **2** (1, 2, 3, 4-tetrahydro-β-carboline-3-carboxylic acid methyl ester) (3 g, 13 mmol) was dissolved in xylene with Pd/C (5%) as a catalyst. And the mixture was refluxed for 48 h, then filtered and distilled under negative-pressure. After separation and purification by column chromatography (DCM: EA = 5: 1), the target product **3** (β-carboline-3-carboxylic acid methyl ester) was obtain. The yield was about 81.8% (2.45 g).

The target product **3** (β-carboline-3-carboxylic acid methyl ester) (300 mg, 1.33 mmol) was dissolved in DMF with NaH as reducer. Then, benzyl bromide was added and the mixture was reacted at room temperature for 6 h. After completion of the reaction as indicated by TLC, the reaction was quenched with ice water, and then extracted with ethyl acetate, washed well with saturated NaCl and then dried with anhydrous Na_2SO_4. After separation and purification by column chromatography (DCM: EA = 2: 1), the target product **4A** (9-benzyl-β-carboline-3-carboxylic acid methyl ester) was obtained. The yield was about 80.4% (313.6 mg).

According to the above method, the target product **3** was reacted with *o*-methylbenzyl chloride, *p*-methylbenzyl chloride and *o*-fluorobenzyl chloride, respectively, to obtain monomers 9-*o*-methylbenzyl-β-carboline-3-carboxylic acid methyl ester (**4B**), 9-*p*-methylbenzyl-β-carboline-3-carboxylic acid methyl ester (**4C**), and 9-*o*-fluorobenzyl-β-carboline-3-carboxylic acid methyl ester (**4D**).

S.2. General reaction of synthesis of β-carboline dimers (**6a-6f**, **6g-6l**, **6m-6r**, and **6s-6x**)

Taking the synthesis of the dimer **6a** as an example: **4A** (9-benzyl-β-carboline-3-carboxylic acid methyl ester) (300 mg, 0.95 mmol) was dissolved in THF/CH_3OH. And then the NaOH solution (0.1 mol/L, 38 mL) was added to give a white precipitate. The mixture was reacted at room temperature until the solution became clear (about 24 h). The reaction solution was extracted with ethyl acetate and dichloromethane. The pH of resulting aqueous phase was adjusted to 5 to give the target product **5** (9-benzyl-β-carboline-3-carboxylic acid).

The target product **5** (60 mg, 0.2 mmol) was dissolved in DMF. And K_2CO_3 (41 mg, 0.3 mmol) and 1, 3-dibromopropane (10 μL, 0.1 mmol) were sequentially added, and the mixture was reacted for about 14 h. The miixture was quenched with ice water, and then extracted with dichloromethane, washed with saturated NaCl and dried with anhydrous Na_2SO_4. After separation and purification by column chromatography (DCM: EA = 5: 1), the target product **6a** (propane-1,3-diyl-bis-(9-benzyl-9H-pyrido [3,4-b]indole-3-carboxylate)) was obtained.

Following the same method, 9-benzyl-β-carboline-3-carboxylic acid methyl ester was reacted with dibromobutane, dibromopentane, dibromohexane, dibromooctane, and dibromoxylene, respectively, to give β-carboline-3-carboxylic acid dimers (**6b-6f**).

Following the same method, 9-*o*-methylbenzyl-β-carboline-3-carboxylic acid methyl ester was reacted with dibromopropane, dibromobutane, dibromopentane,

dibromohexane, dibromooctane, and dibromoxylene, respectively, to give different β-carboline-3-carboxylic acid dimers (**6g-6l**).

Following the same method, 9-*p*-methylbenzyl-β-carboline-3-carboxylic acid methyl ester was reacted with dibromopropane, dibromobutane, dibromopentane, dibromohexane, dibromooctane, and dibromoxylene, respectively, to give different β-carboline-3-carboxylic acid dimers (**6m-6r**).

Following the same method, 9-*o*-fluorobenzyl-β-carboline-3-carboxylic acid methyl ester was reacted with dibromopropane, dibromobutane, dibromopentane, dibromohexane, dibromooctane, and dibromoxylene, respectively, to give different β-carboline-3-carboxylic acid dimers (**6s-6x**).

S.3. The data of yield, melting point, nuclear magnetic and mass spectral

4A (9-benzyl-β-carboline-3-carboxylic acid methyl ester):

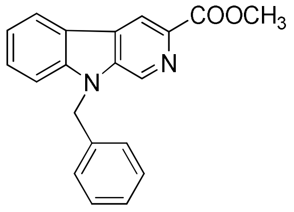

White crystal, yield: 80.4%, m.p.: 186-187 ℃. ESI-MS, m / z: 317.20 $[M+H]^+$. ^{1}H-NMR (500 MHz, $CDCl_3$) δ 8.95–8.89 (m, 2H), 8.23 (d, J = 7.9 Hz, 1H), 7.61 (t, J = 7.7 Hz, 1H), 7.49 (d, J = 8.3 Hz, 1H), 7.39 (d, J = 7.4 Hz, 1H), 7.27 (d, J = 6.0 Hz, 3H), 7.15 (d, J = 7.4 Hz, 2H), 5.62 (s, 2H), 4.06 (s, 3H). ^{13}C-NMR (126 MHz, $CDCl_3$) δ 166.47, 141.84, 138.00, 137.55, 135.71, 131.81, 129.22, 129.06, 128.86, 128.12, 126.54, 122.20, 121.51, 121.08, 117.80, 110.25, 52.72, 47.30.

4B (9-*o*-methylbenzyl-β-carboline-3-carboxylic acid methyl ester):

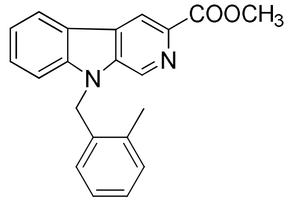

White crystal, yield: 64.7%, m.p.: 147-148 ℃. ESI-MS, m / z: 331.32 $[M+H]^+$. ^{1}H-NMR (500 MHz, CDCl3) δ 8.94 (s, 1H), 8.78 (s, 1H), 8.26 (d, J = 7.8 Hz, 1H), 7.60 (t, J = 7.4 Hz, 1H), 7.44–7.35 (m, 2H), 7.27–7.22 (m, 1H), 7.19 (t, J = 7.4 Hz, 1H), 6.99 (t, J = 7.5 Hz, 1H), 6.57 (d, J = 7.7 Hz, 1H), 5.58 (s, 2H), 4.06 (s, 3H), 2.42 (s, 3H). ^{13}C-NMR (126 MHz, CDCl3) δ 166.48, 141.98, 138.16, 137.53, 135.28, 133.32, 131.86, 130.80, 129.23, 128.87, 127.99, 126.59, 125.90, 122.20, 121.48, 121.11, 117.81, 110.32, 52.73, 45.55, 19.37.

4C (9-*p*-methylbenzyl-β-carboline-3-carboxylic acid methyl ester):

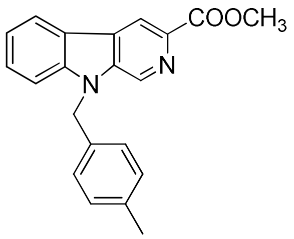

White crystal, yield: 58.9%, m.p.: 157-158 ℃. ESI-MS, m / z: 331.29 $[M+H]^+$. ^{1}H-NMR (500 MHz, CDCl3) δ 8.93 (d, J = 2.2 Hz, 2H), 8.24 (d, J = 7.9 Hz, 1H), 7.66–7.59 (m, 1H), 7.51 (d, J = 8.4 Hz, 1H), 7.42–7.35 (m, 1H), 7.26 (s, 1H), 7.07 (q, J = 8.2 Hz, 4H), 5.59 (s, 2H), 4.06 (s, 3H), 2.29 (s, 3H). ^{13}C-NMR (126 MHz, CDCl3) δ 166.35, 141.93, 137.97, 137.23, 132.62, 131.74, 129.72, 129.27, 128.95, 126.57, 122.21, 121.49, 121.08, 117.83, 110.33, 52.75, 47.17, 21.06.

4D (9-*o*-fluorobenzyl-β-carboline-3-carboxylic acid methyl ester):

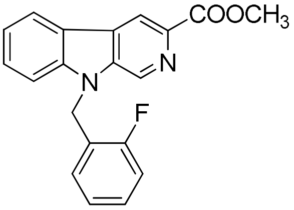

White crystal, yield: 68.3%, m.p.: 175 - 177 ℃. ESI-MS, m / z: 335.30 $[M+H]^+$. ^{1}H-NMR (500 MHz, CDCl3) δ 9.00 (s, 1H), 8.93 (s, 1H), 8.24 (d, J = 7.9 Hz, 1H), 7.67 - 7.62 (m, 1H), 7.56 (d, J = 8.3 Hz, 1H), 7.40 (t, J = 7.5 Hz, 1H), 7.28 - 7.23 (m, 1H), 7.15 - 7.08 (m, 1H), 7.00 - 6.94 (m, 1H), 6.91 - 6.85 (m, 1H), 5.68 (s, 2H), 4.07 (s, 3H).

6a propane-1,3-diyl-bis-(9-benzyl-9H-pyrido[3,4-b]indole-3-carboxylate)

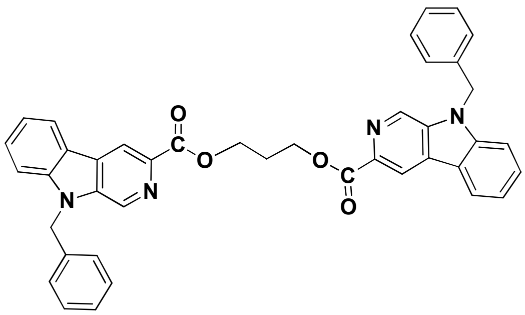

White flake, yield: 57.2%, m.p.: 182-183 ℃. ESI-MS, m / z: 645.38 $[M+H]^{+1}$. H-NMR (500 MHz, CDCl3) δ 8.82 (dd, J = 11.7, 0.7 Hz, 2H), 8.17 (d, J = 7.8 Hz, 1H), 7.61 (ddd, J = 8.2, 7.3, 1.1 Hz, 1H), 7.46 (d, J = 8.3 Hz, 1H), 7.36 (t, J = 7.5 Hz, 1H), 7.26 (d, J = 6.4 Hz, 3H), 7.10 (dd, J = 7.0, 2.4 Hz, 2H), 5.46 (s, 2H), 4.72 (t, J = 6.2 Hz, 2H), 2.50–2.44 (m, 1H). ^{13}C-NMR (126 MHz, CDCl3) δ 165.97, 141.69, 137.87, 137.80, 135.75, 132.04, 129.05, 129.00, 128.52, 128.09, 126.52, 122.22, 121.52, 120.91, 117.76, 110.09, 62.81, 47.11, 28.51.

6b butane-1,4-diyl-bis-(9-benzyl-9H-pyrido[3,4-*b*]indole-3-carboxylate)

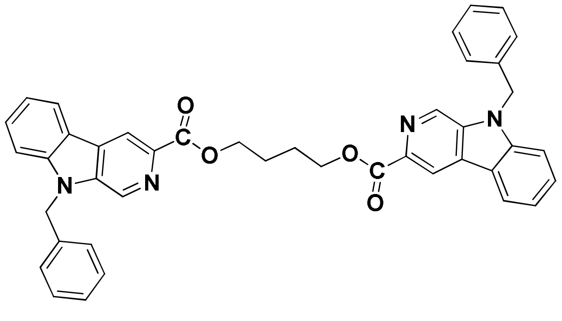

White flake, yield: 56.7%, m.p.: 210-212 ℃. ESI-MS, m / z: 659.40 $[M+H]^{+}$. ^{1}H-NMR (500 MHz, CDCl3) δ 8.89 (d, J = 3.6 Hz, 2H), 8.22 (d, J = 7.8 Hz, 1H), 7.59 (t, J = 7.4 Hz, 1H), 7.48 (d, J = 8.3 Hz, 1H), 7.35 (t, J = 7.5 Hz, 1H), 7.24 (s, 3H), 7.15 – 7.10 (m, 2H), 5.60 (s, 2H), 4.56 (s, 2H), 2.08 (s, 2H). ^{13}C-NMR (126 MHz, CDCl3) δ 166.10, 143.16, 141.86, 138.03, 135.84, 134.33, 132.12, 129.06, 128.74, 128.10, 126.58, 122.23, 121.67, 120.96, 117.71, 110.16, 65.10, 47.32, 25.71.

6c pentane-1,5-diyl-bis-(9-benzyl-9H-pyrido[3,4-*b*]indole-3-carboxylate)

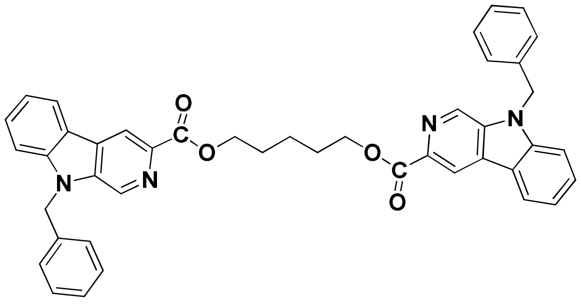

White flake, yield: 77.5%, m.p.: 183-184 ℃. ESI-MS, m / z: 673.45 $[M+H]^+$. ^{1}H-NMR (500 MHz, CDCl3) δ 8.92–8.86 (m, 2H), 8.21 (d, J = 7.9 Hz, 1H), 7.63–7.56 (m, 1H), 7.48 (d, J = 8.3 Hz, 1H), 7.38–7.32 (m, 1H), 7.26–7.25 (m, 3H), 7.13 (dd, J = 7.2, 2.1 Hz, 2H), 5.58 (s, 2H), 4.51 (t, J = 6.8 Hz, 2H), 2.03–1.95 (m, 2H), 1.70 (dt, J = 15.5, 7.7 Hz, 1H). ^{13}C-NMR (126 MHz, CDCl3) δ 166.15, 141.84, 138.19, 138.01, 135.85, 132.11, 129.08, 129.03, 128.75, 128.12, 126.60, 122.23, 121.66, 120.95, 117.68, 110.15, 65.38, 47.31, 28.65, 22.67.

6d hexane-1,6-diyl-bis-(9-benzyl-9H-pyrido[3,4-*b*]indole-3-carboxylate)

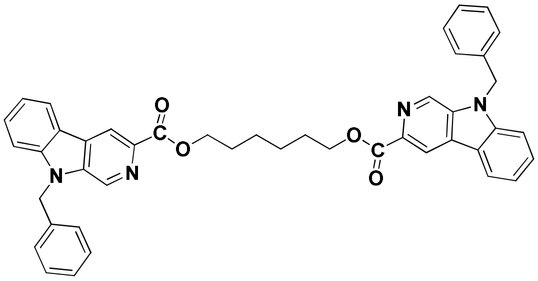

White powder, yield: 71%, m.p.: 184-185 ℃. ESI-MS, m / z: 687.41 $[M+H]^+$. ^{1}H-NMR (500 MHz, CDCl3) δ 8.89 (d, J = 10.8 Hz, 2H), 8.23 (d, J = 7.8 Hz, 1H), 7.63–7.58 (m, 1H), 7.49 (d, J = 8.3 Hz, 1H), 7.36 (t, J = 7.4 Hz, 1H), 7.29–7.24 (m, 3H), 7.17–7.11 (m, 2H), 5.60 (s, 2H), 4.48 (t, J = 6.8 Hz, 2H), 1.97–1.87 (m, 2H), 1.60 (dd, J = 8.7, 5.4 Hz, 2H). ^{13}C-NMR (126 MHz, CDCl3) δ 166.20, 141.82, 138.18,

137.99, 135.85, 132.10, 129.07, 129.04, 128.73, 128.11, 126.59, 122.23, 121.63, 120.95, 117.68, 110.16, 65.58, 47.29, 28.85, 25.87.

6e octane-1,8-diyl-bis-(9-benzyl-9H-pyrido[3,4-*b*]indole-3-carboxylate)

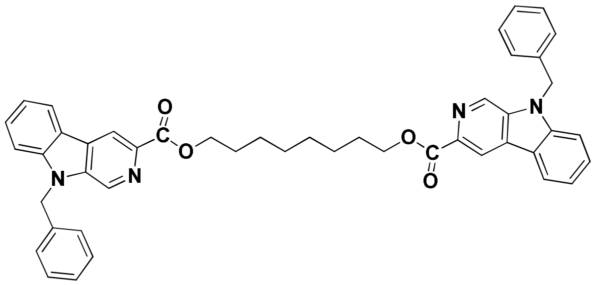

White powder, yield: 57.1%, m.p.: 198-199 ℃. ESI-MS, m / z: 715.49 $[M+H]^+$. ^{1}H-NMR (500 MHz, CDCl3) δ 8.90 (d, J = 14.0 Hz, 2H), 8.23 (d, J = 7.8 Hz, 1H), 7.60 (t, J = 7.5 Hz, 1H), 7.49 (d, J = 8.3 Hz, 1H), 7.37 (t, J = 7.5 Hz, 1H), 7.29–7.21 (m, 3H), 7.17–7.11 (m, 2H), 5.61 (s, 2H), 4.46 (t, J = 6.9 Hz, 2H), 1.93 – 1.83 (m, 2H), 1.46 (dd, J = 26.7, 6.3 Hz, 4H). ^{13}C-NMR (126 MHz, CDCl3) δ 166.20, 141.83, 138.21, 137.98, 135.85, 133.96, 132.09, 129.06, 128.73, 128.10, 126.59, 122.19, 121.63, 120.95, 117.66, 110.17, 65.72, 47.29, 29.24, 28.87, 25.96.

6f 1,4-phenylene-bis-(methylene)-bis-(9-benzyl-9H-pyrido [3,4-*b*]indole-3-carbo-xylate)

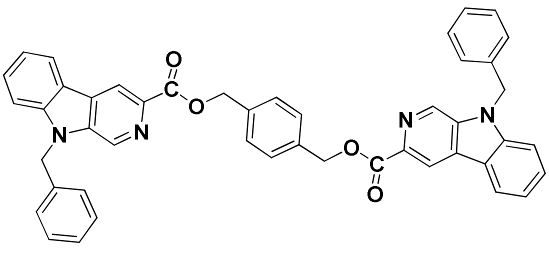

White flake, yield: 53.1%, m.p.: 292-294 ℃. ESI-MS, m / z: 707.22 $[M+H]^+$. ^{1}H-NMR (500 MHz, CDCl3) δ 8.91 (d, J = 8.9 Hz, 2H), 8.23 (d, J = 7.8 Hz, 1H), 7.61 (t, J = 7.7 Hz, 1H), 7.57 (s, 2H), 7.49 (d, J = 8.3 Hz, 1H), 7.36 (t, J = 7.5 Hz, 1H), 7.26 (s, 3H), 7.17–7.11 (m, 2H), 5.63 (s, 2H), 5.51 (s, 2H). ^{13}C-NMR (126 MHz, CDCl3) δ

165.90, 141.82, 138.06, 137.78, 136.28, 135.79, 132.18, 129.07, 128.86, 128.71, 128.11, 126.56, 122.24, 121.61, 121.01, 117.96, 110.20, 66.90, 47.31, 29.71.

6g propane-1,3-diyl-bis-(9-(2-methylbenzyl)-9H-pyrido[3,4-*b*]indole-3-carboxylate)

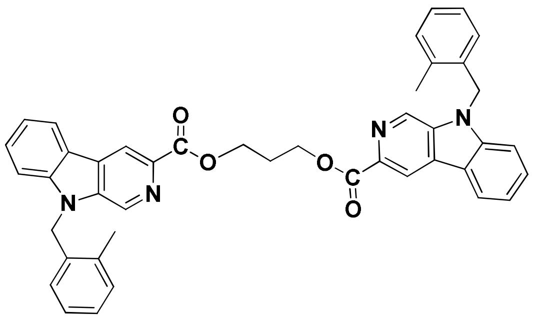

White flake, yield: 57.7%, m.p.: 157-158 ℃. ESI-MS, m / z: 673.43 $[M+H]^+$. ^{1}H-NMR (500 MHz, CDCl3) δ 8.77 (s, 1H), 8.65 (s, 1H), 8.18 (d, J = 7.9 Hz, 1H), 7.59 (t, J = 7.7 Hz, 1H), 7.36 (dd, J = 18.8, 8.0 Hz, 2H), 7.26 (s, 1H), 7.21 (d, J = 7.5 Hz, 1H), 7.16 (t, J = 7.4 Hz, 1H), 6.95 (t, J = 7.5 Hz, 1H), 6.48 (d, J = 7.7 Hz, 1H), 5.32 (s, 2H), 4.72 (t, J = 5.9 Hz, 2H), 2.51–2.42 (m, 1H), 2.37 (s, 3H). ^{13}C-NMR (126 MHz, CDCl3) δ 165.96, 141.77, 137.93, 137.82, 135.18, 133.36, 132.08, 130.75, 128.97, 128.41, 127.93, 126.59, 125.84, 122.26, 121.48, 120.92, 117.71, 110.12, 62.95, 45.22, 28.50, 19.36.

6h butane-1,4-diyl-bis-(9-(2-methylbenzyl)-9H-pyrido[3,4-*b*]indole-3-carboxylate)

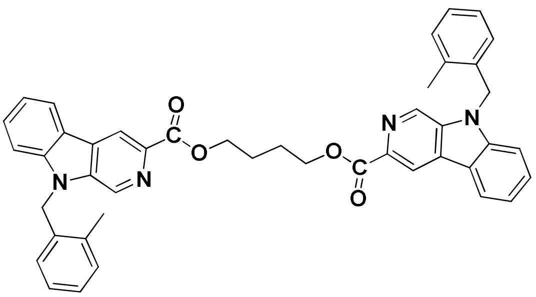

White flake, yield: 44.2%, m.p.: 211-213 ℃. ESI-MS m / z: 687.40 $[M+H]^+$. ^{1}H NMR (500 MHz, $CDCl_3$) δ 8.96 (d, *J* = 0.7 Hz, 1H), 8.82 (d, *J* = 0.5 Hz, 1H), 8.30 (d,

J = 7.8 Hz, 1H), 7.67–7.60 (m, 1H), 7.46–7.39 (m, 2H), 7.31 (s, 1H), 7.27 (d, J = 7.4 Hz, 1H), 7.22 (t, J = 7.4 Hz, 1H), 7.03 (t, J = 7.4 Hz, 1H), 6.62 (d, J = 7.7 Hz, 1H), 5.61 (s, 2H), 4.62 (s, 2H), 2.45 (s, 3H), 2.14 (t, J = 2.7 Hz, 2H). ^{13}C NMR (126 MHz, $CDCl_3$) δ 166.12, 141.94, 138.15, 137.99, 135.28, 133.43, 132.18, 130.78, 129.05, 128.70, 127.96, 126.61, 125.99, 122.24, 121.58, 120.99, 117.76, 110.24, 65.12, 45.51, 25.69, 19.37.

6i pentane-1,5-diyl-bis-(9-(2-methylbenzyl)-9H-pyrido[3,4-*b*]indole-3-carboxylate)

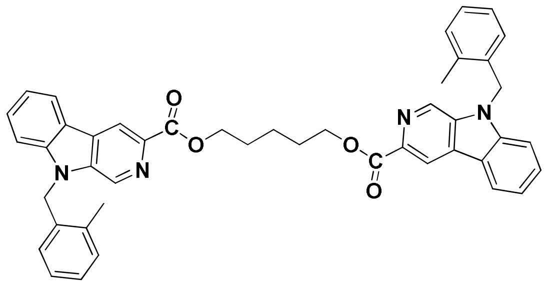

White flake, yield: 37.9%, m.p.: 145-146 ℃. ESI-MS m / z: 701.57 $[M+H]^+$. ^{1}H NMR (500 MHz, $CDCl_3$) δ 8.93 (d, J = 0.6 Hz, 1H), 8.80 (d, J = 0.4 Hz, 1H), 8.27 (d, J = 7.8 Hz, 1H), 7.66–7.59 (m, 1H), 7.41 (dd, J = 15.6, 7.8 Hz, 2H), 7.31 (s, 1H), 7.27 (d, J = 7.4 Hz, 1H), 7.22 (t, J = 7.4 Hz, 1H), 7.02 (t, J = 7.4 Hz, 1H), 6.61 (d, J = 7.7 Hz, 1H), 5.57 (s, 2H), 4.56 (t, J = 6.8 Hz, 2H), 2.45 (s, 3H), 2.08–1.99 (m, 2H), 1.78–1.72 (m, 1H). ^{13}C NMR (126 MHz, $CDCl_3$) δ 166.15, 141.91, 138.10, 138.07, 135.27, 133.43, 132.16, 130.78, 129.01, 128.67, 127.96, 126.60, 125.99, 122.21, 121.56, 120.95, 117.68, 110.19, 65.36, 45.47, 28.61, 22.61, 19.36.

6j hexane-1,6-diyl-bis-(9-(2-methylbenzyl)-9H-pyrido[3,4-*b*]indole-3-carboxylate)

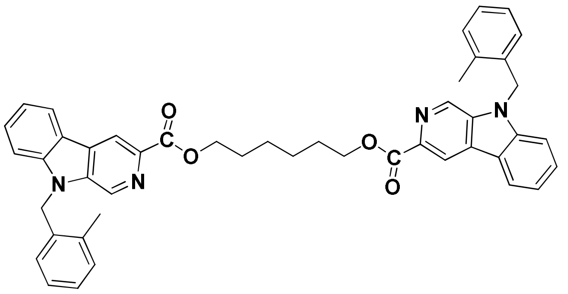

White powder, yield: 32.6%, m.p.: 189-190 ℃. ESI-MS m / z: 715.55 [M+H]$^{+}$. ^{1}H NMR (500 MHz, CDCl$_3$) δ 8.90 (d, *J* = 0.8 Hz, 1H), 8.77 (d, *J* = 0.7 Hz, 1H), 8.25 (d, *J* = 7.8 Hz, 1H), 7.59 (ddd, *J* = 8.2, 7.2, 1.1 Hz, 1H), 7.42–7.34 (m, 2H), 7.26 (s, 1H), 7.23 (d, *J* = 7.4 Hz, 1H), 7.18 (t, *J* = 7.4 Hz, 1H), 6.98 (t, *J* = 7.4 Hz, 1H), 6.58 (d, *J* = 7.7 Hz, 1H), 5.57 (s, 2H), 4.48 (t, *J* = 6.8 Hz, 2H), 2.41 (s, 3H), 1.96–1.88 (m, 2H). ^{13}C NMR (126 MHz, CDCl$_3$) δ 166.20, 141.94, 138.13, 135.29, 133.45, 132.16, 130.78, 129.04, 128.71, 127.96, 126.60, 126.00, 122.22, 121.59, 120.97, 117.68, 110.22, 65.58, 45.50, 28.84, 25.86, 19.37.

6k octane-1,8-diyl-bis-(9-(2-methylbenzyl)-9H-pyrido[3,4-*b*]indole-3-carboxylate)

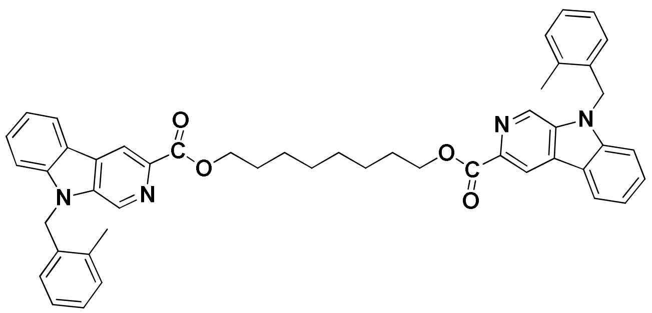

White powder, yield: 25.2%, m.p.: 223-225 ℃. ESI-MS m / z: 743.62 [M+H]$^{+}$. ^{1}H NMR (500 MHz, CDCl$_3$) δ 8.90 (s, 1H), 8.78 (s, 1H), 8.26 (d, *J* = 7.8 Hz, 1H), 7.59 (t, *J* = 7.7 Hz, 1H), 7.42–7.35 (m, 2H), 7.26 (s, 1H), 7.23 (d, *J* = 7.5 Hz, 1H), 7.18 (t, *J* = 7.3 Hz, 1H), 6.98 (t, *J* = 7.5 Hz, 1H), 6.58 (d, *J* = 7.7 Hz, 1H), 5.58 (s, 2H), 4.46 (t, *J* = 6.9 Hz, 2H), 2.41 (s, 3H), 1.92–1.83 (m, 2H), 1.47 (dd, *J* = 32.5, 6.2 Hz, 4H). ^{13}C NMR (126 MHz, CDCl$_3$) δ 166.22, 141.95, 138.18, 138.13, 135.29, 133.46,

132.16, 130.78, 129.04, 128.72, 127.96, 126.60, 126.00, 122.19, 121.58, 120.97, 117.67, 110.23, 65.72, 45.52, 29.24, 28.87, 25.96, 19.37.

6l 1,4-phenylene-bis-(methylene)-bis-(9-(2-methylbenzyl)-9H-pyrido[3,4-*b*]indole-3-carboxylate)

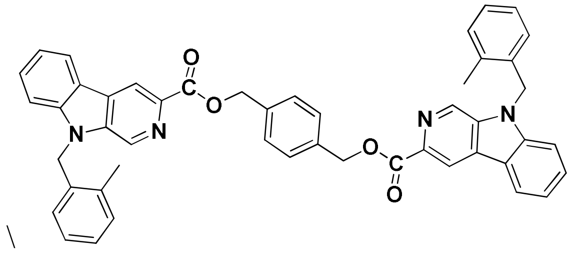

White flake, yield: 59.2%, m.p.: 280-281 ℃. ESI-MS m / z: 735.29 $[M+H]^+$. ^1H NMR (500 MHz, $CDCl_3$) δ 8.97 (s, 1H), 8.84 (s, 1H), 8.29 (d, *J* = 7.9 Hz, 1H), 7.61 (s, 2H), 7.42 (dd, *J* = 16.1, 8.0 Hz, 2H), 7.31 (s, 3H), 7.28 (d, *J* = 7.5 Hz, 1H), 7.22 (t, *J* = 7.4 Hz, 1H), 7.03 (t, *J* = 7.5 Hz, 1H), 6.62 (d, *J* = 7.7 Hz, 1H), 5.64 (s, 2H), 5.56 (s, 2H), 2.46 (s, 3H). ^{13}C NMR (126 MHz, $CDCl_3$) δ 165.91, 141.95, 138.21, 137.77, 136.29, 135.29, 133.41, 132.26, 130.79, 129.08, 128.86, 128.70, 127.98, 126.61, 125.98, 122.23, 121.57, 121.02, 117.96, 110.26, 66.89, 45.54, 19.37.

6m propane-1,3-diyl-bis-(9-(4-methylbenzyl)-9H-pyrido[3,4-*b*]indole-3-carboxylate)

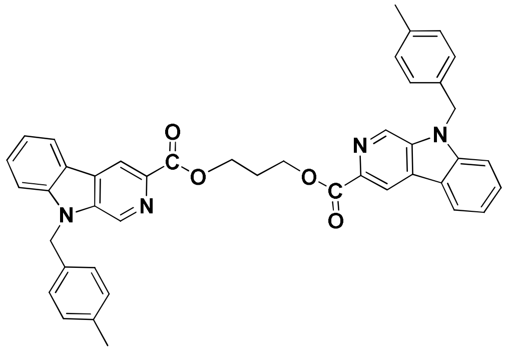

White flake, yield: 62%, m.p.: 188-189 ℃. ESI-MS m / z: 673.45 $[M+H]^+$. ^1H NMR (500 MHz, $CDCl_3$) δ 8.93 (s, 1H), 8.85 (s, 1H), 8.21 (d, *J* = 7.8 Hz, 1H), 7.66 (t,

J = 7.6 Hz, 1H), 7.51 (d, J = 8.3 Hz, 1H), 7.40 (t, J = 7.3 Hz, 1H), 7.31 (s, 1H), 7.10 (d, J = 8.0 Hz, 2H), 7.04 (d, J = 8.0 Hz, 2H), 5.46 (s, 2H), 4.77 (t, J = 6.2 Hz, 2H), 2.55–2.49 (m, 1H), 2.31 (s, 3H). ^{13}C NMR (126 MHz, $CDCl_3$) δ 165.51, 141.86, 137.94, 137.73, 136.95, 132.55, 131.69, 129.69, 129.23, 128.78, 126.54, 122.27, 121.38, 121.02, 117.83, 110.23, 62.94, 46.99, 28.42, 21.05.

6n butane-1,4-diyl-bis-(9-(4-methylbenzyl)-9H-pyrido[3,4-*b*]indole-3-carboxylate)

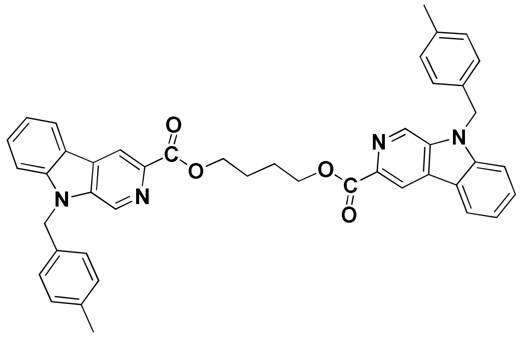

White flake, yield: 47%, m.p.: 223-225 ℃. ESI-MS m / z: 687.46 $[M+H]^+$. ^1H NMR (500 MHz, $CDCl_3$) δ 8.90 (d, J = 8.3 Hz, 2H), 8.23 (d, J = 7.8 Hz, 1H), 7.60 (t, J = 7.7 Hz, 1H), 7.50 (d, J = 8.3 Hz, 1H), 7.37 (s, 1H), 7.26 (s, 1H), 7.06 (q, J = 8.2 Hz, 4H), 5.57 (s, 2H), 4.57 (s, 2H), 2.28 (s, 3H), 2.10 (s, 2H). ^{13}C NMR (126 MHz, $CDCl_3$) δ 166.14, 141.80, 137.99, 137.89, 137.83, 132.77, 132.15, 129.70, 129.01, 128.66, 126.59, 122.21, 121.58, 120.89, 117.77, 110.22, 65.12, 47.10, 25.67, 21.05.

6o pentane-1,5-diyl-bis-(9-(4-methylbenzyl)-9H-pyrido[3,4-*b*]indole-3-carboxylate)

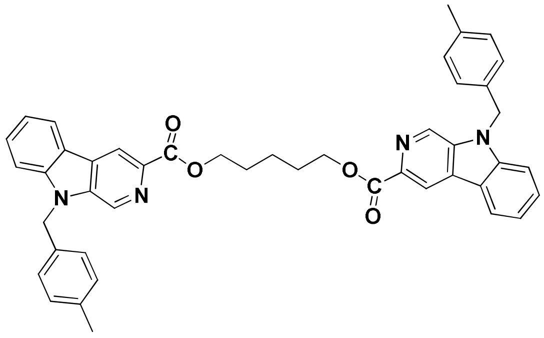

White flake, yield: 50%, m.p.: 109-111 ℃. ESI-MS m / z: 701.67 $[M+H]^+$. ^{1}H NMR (500 MHz, $CDCl_3$) δ 8.92 (d, *J* = 10.5 Hz, 2H), 8.24 (d, *J* = 7.8 Hz, 1H), 7.64 (t, *J* = 7.7 Hz, 1H), 7.53 (d, *J* = 8.3 Hz, 1H), 7.38 (t, *J* = 7.5 Hz, 1H), 7.31 (s, 1H), 7.09 (q, *J* = 8.1 Hz, 4H), 5.57 (s, 2H), 4.55 (t, *J* = 6.8 Hz, 2H), 2.32 (s, 3H), 2.18–1.95 (m, 2H). ^{13}C NMR (126 MHz, $CDCl_3$) δ 166.17, 141.77, 137.95, 137.90, 132.76, 132.13, 129.69, 128.98, 128.65, 126.58, 122.18, 121.55, 120.87, 117.70, 110.18, 109.32, 65.36, 47.07, 28.60, 22.62, 21.05.

6p hexane-1,6-diyl-bis-(9-(4-methylbenzyl)-9H-pyrido[3,4-*b*]indole-3-carboxylate)

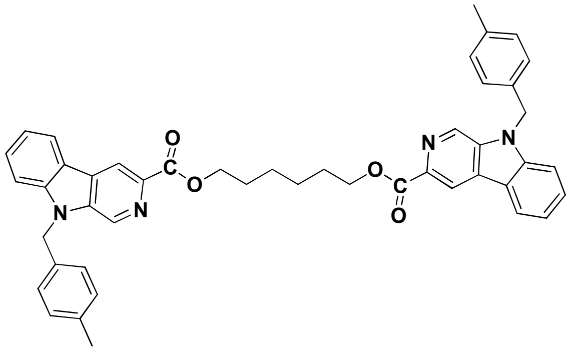

White flake, yield: 69%, m.p.: 115-117 ℃. ESI-MS m / z: 715.59 $[M+H]^+$. ^{1}H NMR (500 MHz, $CDCl_3$) δ 8.90 (d, *J* = 15.9 Hz, 2H), 8.23 (d, *J* = 7.8 Hz, 1H), 7.63 – 7.57 (m, 1H), 7.50 (d, *J* = 8.3 Hz, 1H), 7.38 (s, 1H), 7.26 (s, 1H), 7.06 (q, *J* = 8.3 Hz, 4H), 5.56 (s, 2H), 4.48 (t, *J* = 6.8 Hz, 2H), 2.28 (s, 3H), 1.98–1.88 (m, 2H), 1.62–1.58 (m, 2H). ^{13}C NMR (126 MHz, $CDCl_3$) δ 166.21, 141.80, 137.97, 137.89, 132.78, 132.13, 129.69, 129.00, 128.67, 126.60, 122.19, 121.59, 120.87, 117.69, 110.20, 65.58, 47.10, 29.71, 28.83, 25.85, 21.05.

6q octane-1,8-diyl-bis-(9-(4-methylbenzyl)-9H-pyrido[3,4-*b*]indole-3-carboxylate)

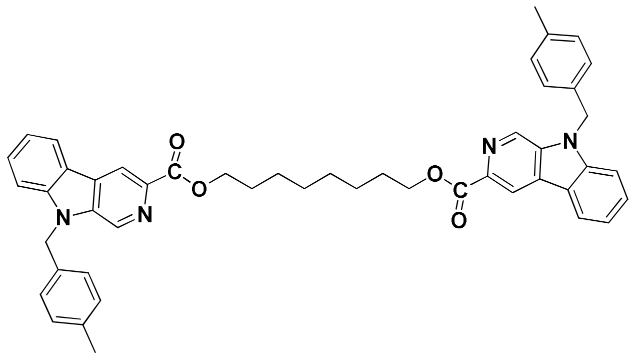

White flake, yield: 47.6%, m.p.: 178-180 ℃. ESI-MS m / z: 743.55 $[M+H]^+$. ^1H NMR (500 MHz, $CDCl_3$) δ 8.90 (d, J = 17.5 Hz, 2H), 8.23 (d, J = 7.9 Hz, 1H), 7.60 (t, J = 7.7 Hz, 1H), 7.50 (d, J = 8.3 Hz, 1H), 7.36 (t, J = 7.5 Hz, 1H), 7.26 (s, 1H), 7.06 (q, J = 8.1 Hz, 4H), 5.57 (s, 2H), 4.46 (t, J = 6.9 Hz, 2H), 2.28 (s, 3H), 1.93–1.83 (m, 2H), 1.46 (d, J = 33.2 Hz, 4H). ^{13}C NMR (126 MHz, $CDCl_3$) δ 166.23, 141.81, 138.04, 137.97, 137.90, 132.80, 132.14, 129.69, 129.00, 128.68, 126.60, 122.16, 121.59, 120.87, 117.67, 110.21, 65.72, 47.10, 29.25, 28.86, 25.96, 21.05.

6r 1,4-phenylene-bis-(methylene)-bis-(9-(4-methylbenzyl)-9H-pyrido[3,4-*b*]indole-3-carboxylate)

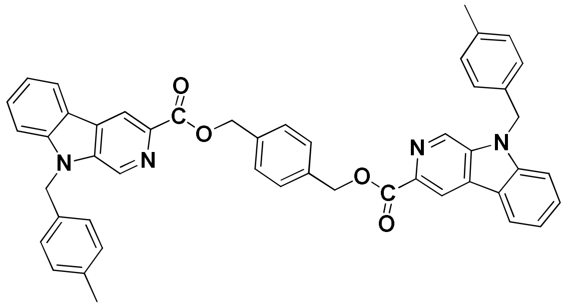

White flake, yield: 34.4%, m.p.: 266-268 ℃. ESI-MS m / z: 735.32 $[M+H]^+$. ^1H NMR (500 MHz, $CDCl_3$) δ 8.99 (s, 1H), 8.91 (s, 1H), 8.24 (d, J = 7.8 Hz, 1H), 7.63 (t, J = 7.6 Hz, 1H), 7.58 (s, 2H), 7.52 (d, J = 8.3 Hz, 1H), 7.38 (t, J = 7.5 Hz, 1H), 7.26 (s, 2H), 7.06 (dd, J = 16.8, 7.8 Hz, 4H), 5.60 (s, 2H), 5.53 (s, 2H), 2.28 (s, 3H). ^{13}C NMR (126 MHz, $CDCl_3$) δ 165.19, 142.11, 138.01, 137.86, 136.61, 136.14, 132.50,

131.64, 129.74, 129.48, 129.15, 128.96, 126.57, 122.35, 121.42, 121.21, 118.05, 110.39, 67.08, 47.26, 21.06.

6s propane-1,3-diyl-bis-(9-(2-fluorobenzyl)-9H-pyrido[3,4-*b*]indole-3-carboxylate)

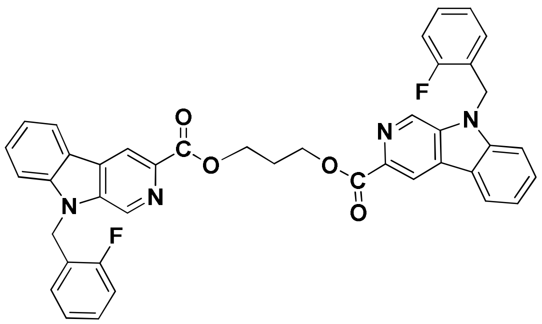

White flake, yield: 65.4%, m.p.: 208-210 ℃. ESI-MS m / z: 681.49 $[M+H]^+$. ^{1}H NMR (500 MHz, $CDCl_3$) δ 8.86 (s, 1H), 8.77 (s, 1H), 8.14 (d, *J* = 7.9 Hz, 1H), 7.62 (t, *J* = 7.7 Hz, 1H), 7.48 (d, *J* = 8.3 Hz, 1H), 7.36 (t, *J* = 7.5 Hz, 1H), 7.28–7.20 (m, 2H), 7.10 (t, *J* = 9.3 Hz, 1H), 6.93 (t, *J* = 7.5 Hz, 1H), 6.79 (t, *J* = 7.6 Hz, 1H), 5.45 (s, 2H), 4.72 (t, J = 5.8 Hz, 2H), 2.50–2.43 (m, 1H), 1.68 (s, 3H). ^{13}C NMR (126 MHz, CDCl3) δ 165.93, 159.41, 141.50, 137.92, 137.75, 131.94, 129.97, 129.91, 129.09, 128.56, 128.42, 128.39, 124.67, 124.64, 122.86, 122.75, 122.21, 121.49, 121.03, 117.73, 115.87, 115.70, 109.99, 62.95, 40.82, 29.72, 28.51.

6t butane-1,4-diyl-bis-(9-(2-fluorobenzyl)-9H-pyrido[3,4-*b*]indole-3-carboxylate)

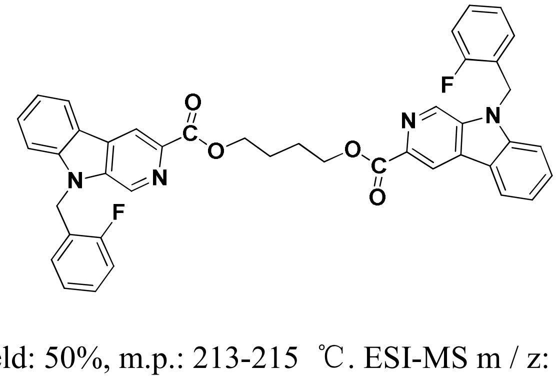

Needle crystal, yield: 50%, m.p.: 213-215 ℃. ESI-MS m / z: 695.69 $[M+H]^+$. ^{1}H NMR (500 MHz, $CDCl_3$) δ 8.97 (s, 1H), 8.90 (s, 1H), 8.23 (d, *J* = 7.8 Hz, 1H), 7.63 (t,

J = 7.7 Hz, 1H), 7.54 (d, *J* = 8.3 Hz, 1H), 7.38 (t, *J* = 7.5 Hz, 1H), 7.29–7.21 (m, 2H), 7.15–7.07 (m, 1H), 6.95 (t, *J* = 7.5 Hz, 1H), 6.86 (t, *J* = 7.1 Hz, 1H), 5.66 (s, 2H), 4.58 (s, 2H), 2.11 (s, 2H). ^{13}C NMR (126 MHz, $CDCl_3$) δ 166.08, 161.41, 159.45, 141.65, 138.10, 137.91, 132.00, 129.97, 129.91, 129.13, 128.80, 128.47, 128.44, 124.67, 124.64, 122.80, 122.24, 121.61, 121.09, 117.75, 115.88, 115.72, 110.09, 65.15, 40.99, 29.71, 25.67.

6u pentane-1,5-diyl-bis-(9-(2-fluorobenzyl)-9H-pyrido[3,4-*b*]indole-3-carboxylate)

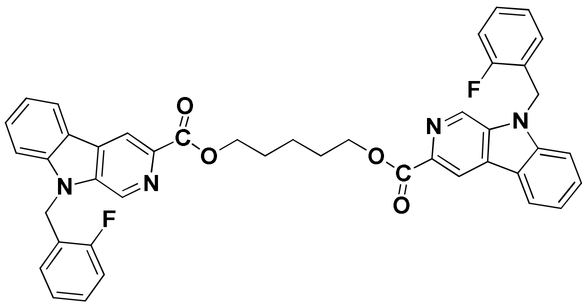

White flake, yield: 67.8%, m.p.: 187-188 ℃. ESI-MS m / z: 709.39 $[M+H]^+$. ^{1}H NMR (500 MHz, $CDCl_3$) δ 8.94 (s, 1H), 8.87 (s, 1H), 8.19 (d, *J* = 7.8 Hz, 1H), 7.61 (t, *J* = 7.7 Hz, 1H), 7.52 (d, *J* = 8.3 Hz, 1H), 7.35 (t, *J* = 7.5 Hz, 1H), 7.28–7.20 (m, 2H), 7.15–7.06 (m, 1H), 6.94 (t, *J* = 7.5 Hz, 1H), 6.84 (t, *J* = 7.1 Hz, 1H), 5.61 (s, 2H), 4.52 (t, *J* = 6.8 Hz, 2H), 2.07–1.94 (m, 2H), 1.71 (dd, *J* = 9.3, 6.2 Hz, 1H). ^{13}C NMR (126 MHz, $CDCl_3$) δ 166.11, 161.40, 159.44, 141.61, 138.19, 137.86, 131.99, 129.97, 129.91, 129.09, 128.76, 128.47, 128.44, 124.66, 124.63, 122.90, 122.79, 122.19, 121.57, 121.05, 117.67, 115.88, 115.71, 110.04, 65.38, 40.98, 40.94, 29.71, 28.59, 22.61.

6v hexane-1,6-diyl-bis-(9-(2-fluorobenzyl)-9H-pyrido[3,4-*b*]indole-3-carboxylate)

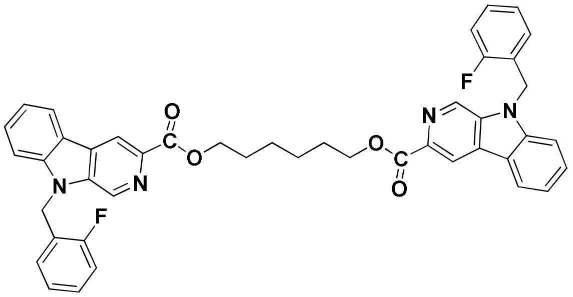

White solid, yield: 54.6%, m.p.: 199-201 ℃. ESI-MS m / z: 723.48 $[M+H]^+$. ^{1}H NMR (500 MHz, $CDCl_3$) δ 8.96 (s, 1H), 8.88 (s, 1H), 8.23 (d, *J* = 7.8 Hz, 1H), 7.63 (t, *J* = 7.7 Hz, 1H), 7.53 (d, *J* = 8.3 Hz, 1H), 7.38 (t, *J* = 7.5 Hz, 1H), 7.29–7.20 (m, 2H), 7.16–7.07 (m, 1H), 6.95 (t, *J* = 7.5 Hz, 1H), 6.86 (t, *J* = 7.5 Hz, 1H), 5.65 (s, 2H), 4.49 (t, *J* = 6.8 Hz, 2H), 2.05–1.89 (m, 2H), 1.61 (d, *J* = 7.0 Hz, 2H). ^{13}C NMR (126 MHz, $CDCl_3$) δ 166.16, 161.42, 159.45, 141.65, 138.26, 137.88, 131.98, 129.98, 129.91, 129.12, 128.80, 128.49, 128.46, 124.66, 124.64, 122.92, 122.22, 121.61, 121.07, 117.67, 115.88, 115.72, 110.07, 65.61, 41.02, 40.98, 29.71, 28.83, 25.85.

6w octane-1,8-diyl-bis-(9-(2-fluorobenzyl)-9H-pyrido[3,4-*b*]indole-3-carboxylate)

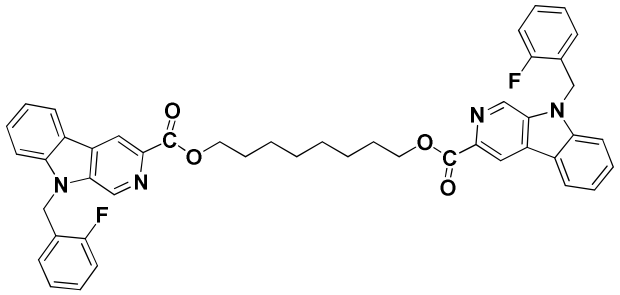

White solid, yield: 65%, m.p.: 148-150 ℃. ESI-MS m / z: 751.48 $[M+H]^+$. ^{1}H NMR (500 MHz, $CDCl_3$) δ 8.97 (s, 1H), 8.89 (s, 1H), 8.23 (d, *J* = 7.9 Hz, 1H), 7.62 (t, *J* = 7.7 Hz, 1H), 7.54 (d, *J* = 8.3 Hz, 1H), 7.38 (t, *J* = 7.5 Hz, 1H), 7.25 (dd, *J* = 12.7, 4.3 Hz, 2H), 7.11 (t, *J* = 9.2 Hz, 1H), 6.95 (t, *J* = 7.5 Hz, 1H), 6.86 (t, *J* = 7.5 Hz, 1H), 5.66 (s, 2H), 4.46 (t, *J* = 6.7 Hz, 2H), 1.93–1.83 (m, 2H), 1.50 (s, 2H), 1.43 (s, 2H).

^{13}C NMR (126 MHz, $CDCl_3$) δ 166.18, 159.46, 141.66, 138.31, 137.89, 131.99, 129.98, 129.92, 129.12, 128.82, 128.47, 124.66, 124.63, 122.93, 122.82, 122.19, 121.62, 121.07, 117.66, 115.89, 115.72, 110.09, 65.75, 41.03, 40.99, 29.71, 29.24, 28.86, 25.95.

6x 1,4-phenylenebis(methylene)-bis-(9-(2-fluorobenzyl)-9H-pyrido[3,4-*b*]indole-3-carboxylate)

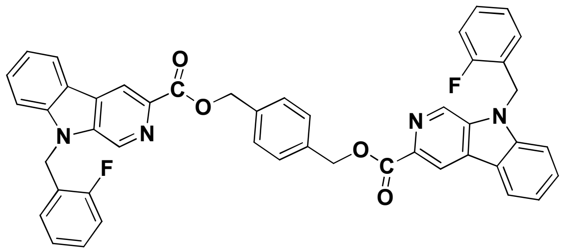

White flake solid, yield: 61.2%, m.p.: 277-279 ℃. ESI-MS m / z: 743.56[M+H]$^+$. ^{1}H NMR (500 MHz, $CDCl_3$) δ 8.96 (s, 1H), 8.91 (s, 1H), 8.24 (d, *J* = 7.8 Hz, 1H), 7.69–7.62 (m, 1H), 7.61–7.54 (m, 3H), 7.39 (t, *J* = 7.5 Hz, 1H), 7.33 (s, 1H), 7.26 (dd, *J* = 13.9, 6.9 Hz, 1H), 7.17–7.09 (m, 1H), 6.96 (t, *J* = 7.5 Hz, 1H), 6.87 (t, *J* = 7.5 Hz, 1H), 5.70 (s, 2H), 5.52 (s, 2H). ^{13}C NMR (126 MHz, $CDCl_3$) δ 165.71, 141.73, 137.98, 136.13, 131.86, 129.97, 129.91, 129.30, 128.81, 128.38, 124.61, 122.19, 121.42, 121.19, 117.97, 115.83, 115.66, 110.20, 66.91, 49.46, 49.29, 49.12, 48.95, 48.78, 41.08, 29.64.

S.4. HNMR and CNMR

4A

H NMR

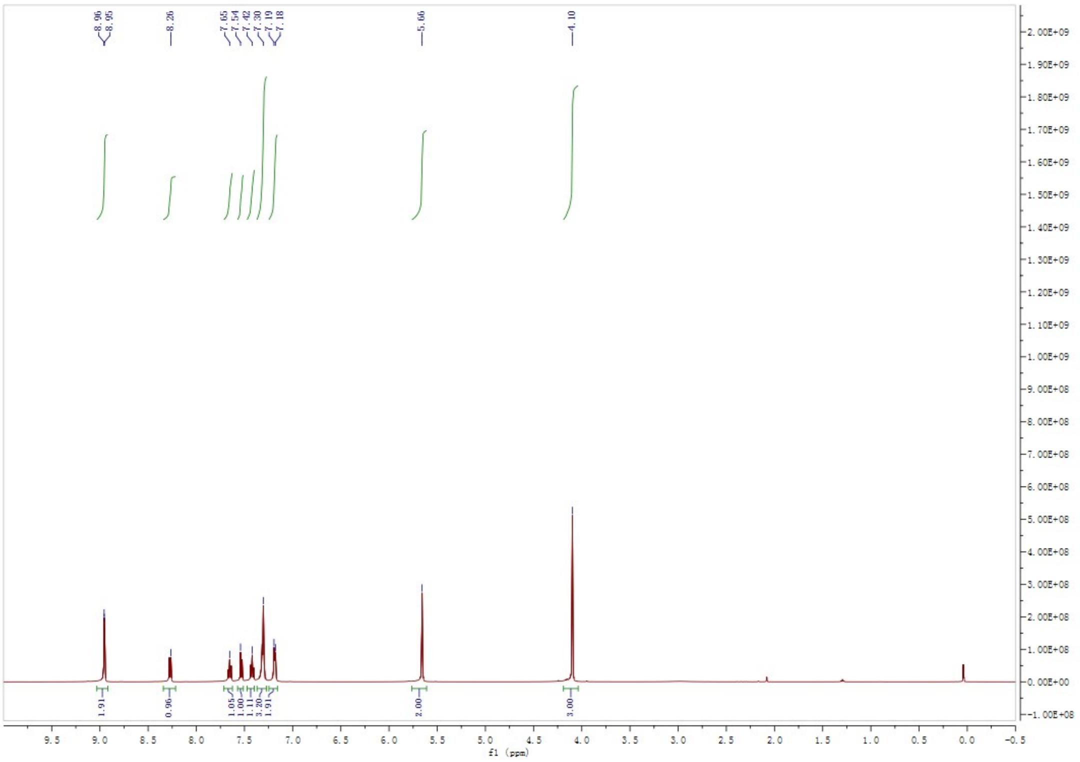

C NMR

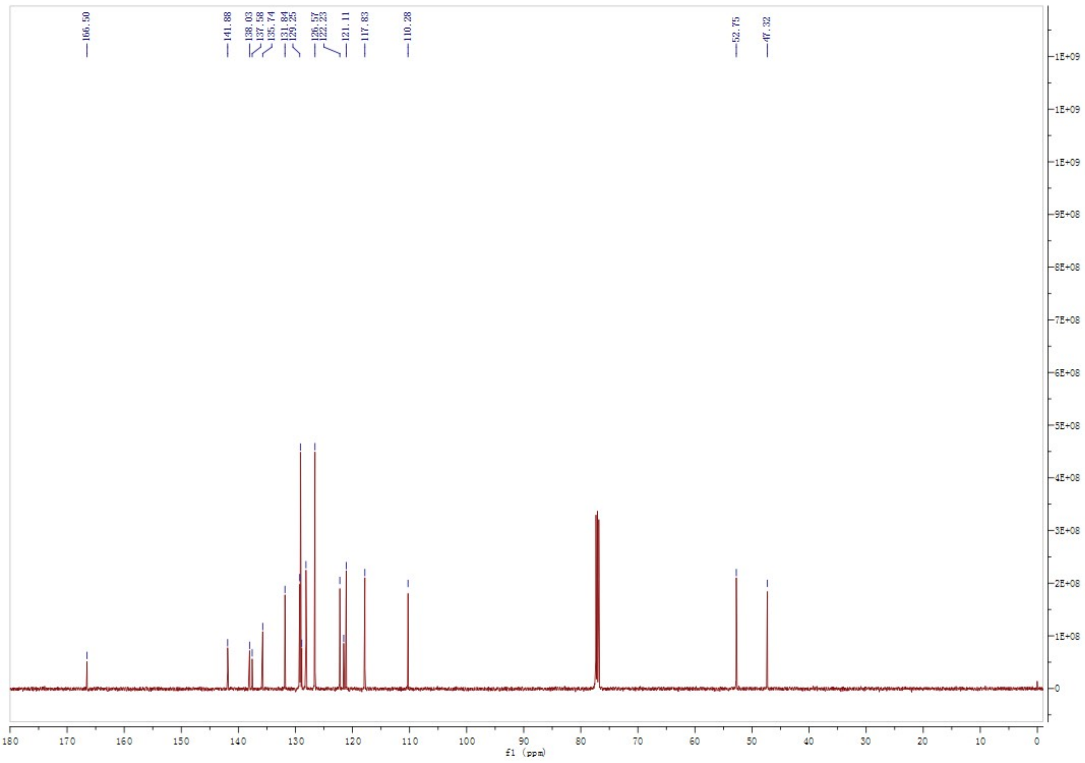
166.50
141.88
138.03
137.58
135.74
131.84
129.25
126.57
122.23
121.11
117.83
110.28
52.75
47.32
f1 (ppm)

4B

H NMR

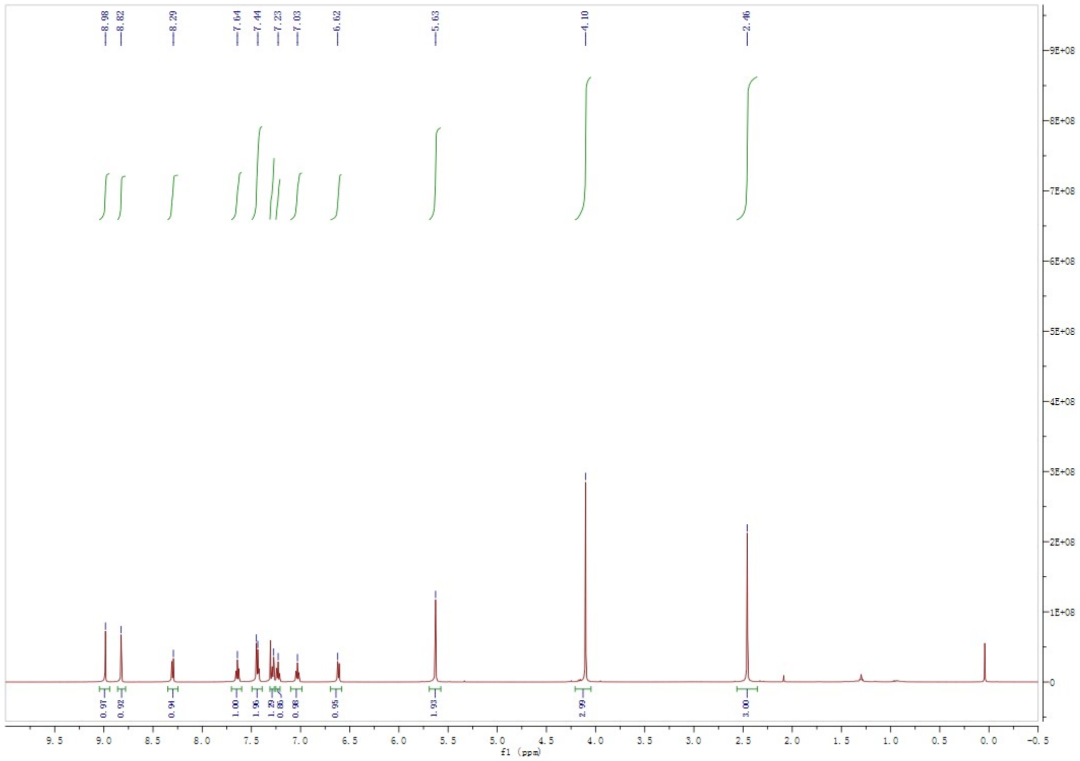
8.98
8.82
8.29
7.64
7.44
7.23
7.03
6.62
5.63
4.10
2.46
0.97
0.92
0.94
1.00
1.96
1.29
0.86
0.98
0.95
1.93
2.99
3.00
f1 (ppm)

C NMR

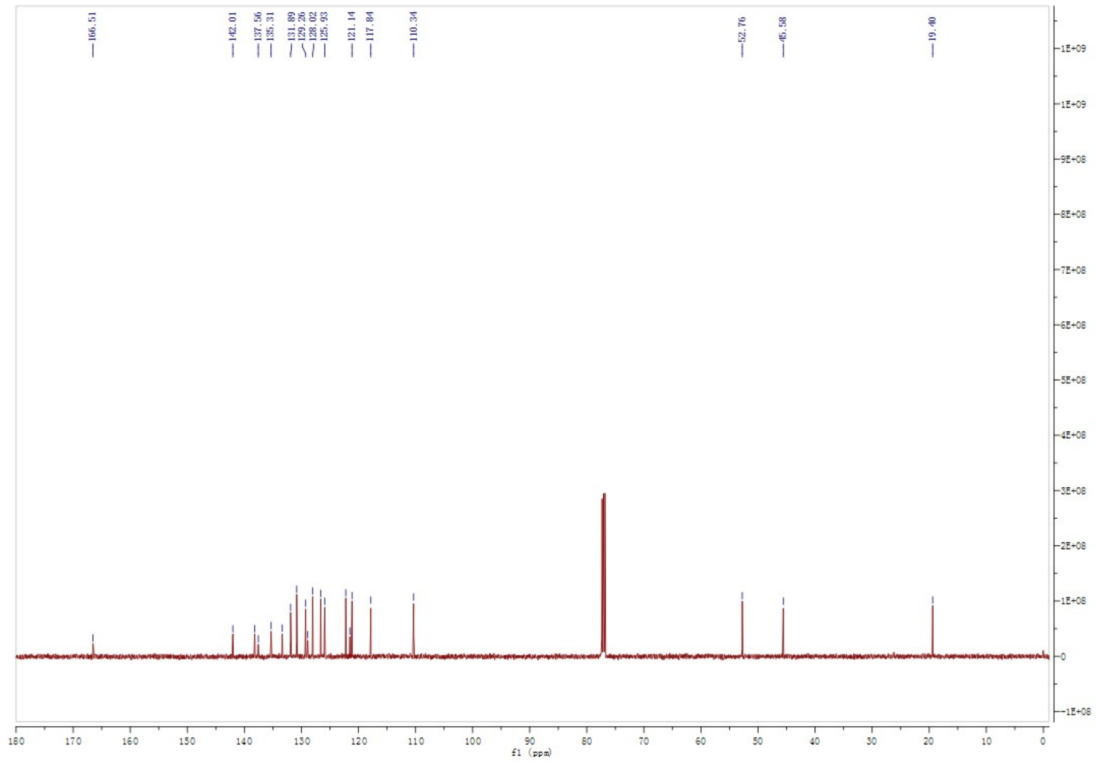
166.51
142.01
137.56
135.31
131.89
129.26
128.02
125.93
121.14
117.84
110.34
52.76
45.58
19.40
f1 (ppm)

4C

H NMR

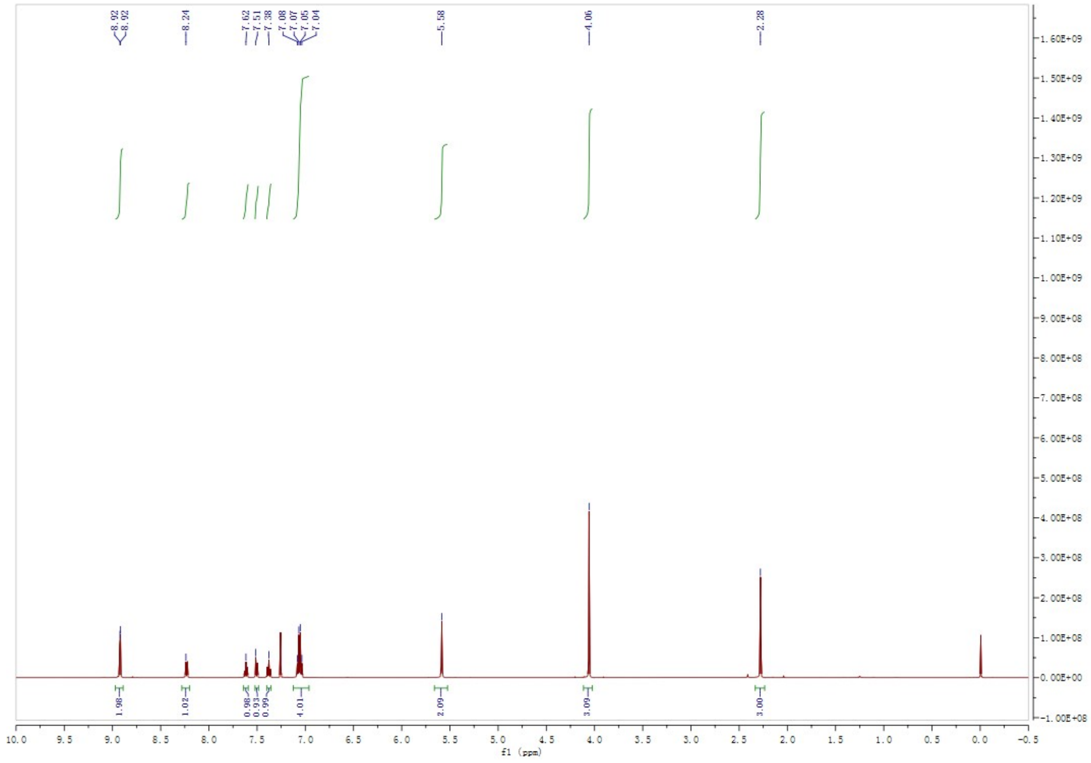
8.92
8.92
8.24
7.62
7.51
7.38
7.08
7.07
7.05
7.04
5.58
4.06
2.28
1.98
1.02
0.98
0.93
0.99
4.01
2.09
3.09
3.00
f1 (ppm)

C NMR

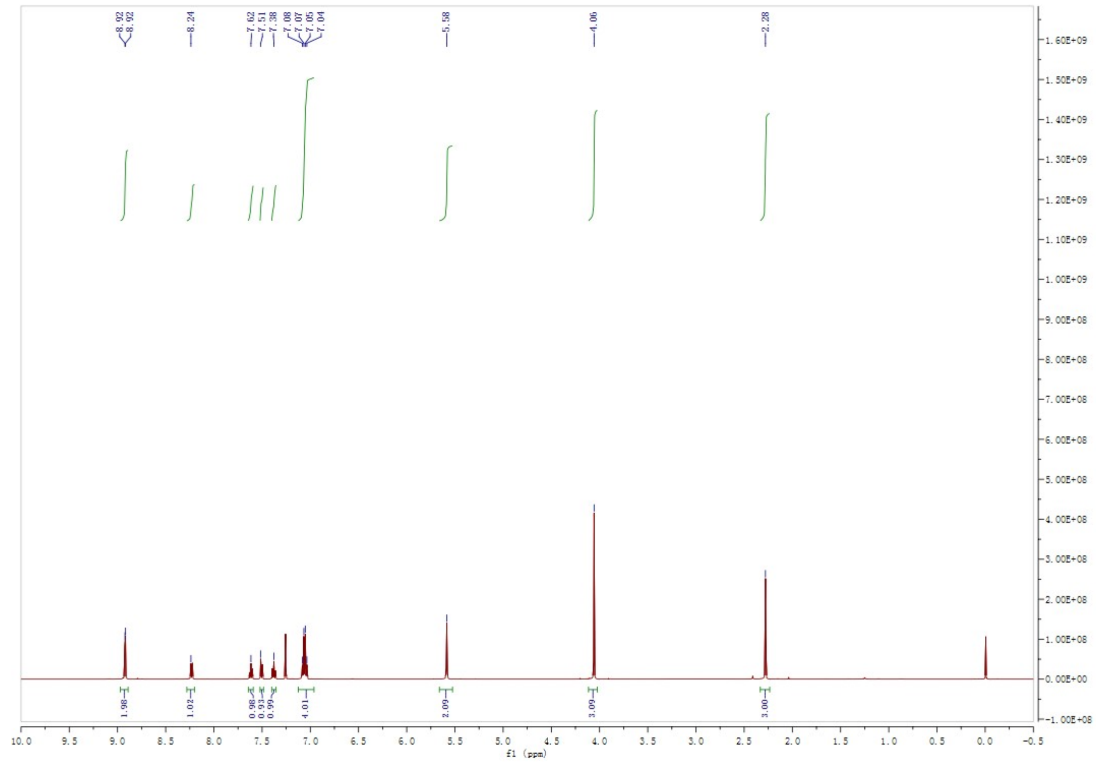
8.92
8.92
8.24
7.62
7.51
7.38
7.08
7.07
7.05
7.04
5.58
4.06
2.28
1.98
1.02
0.98
0.93
0.99
4.01
2.09
3.09
3.00
f1 (ppm)

4D

H NMR

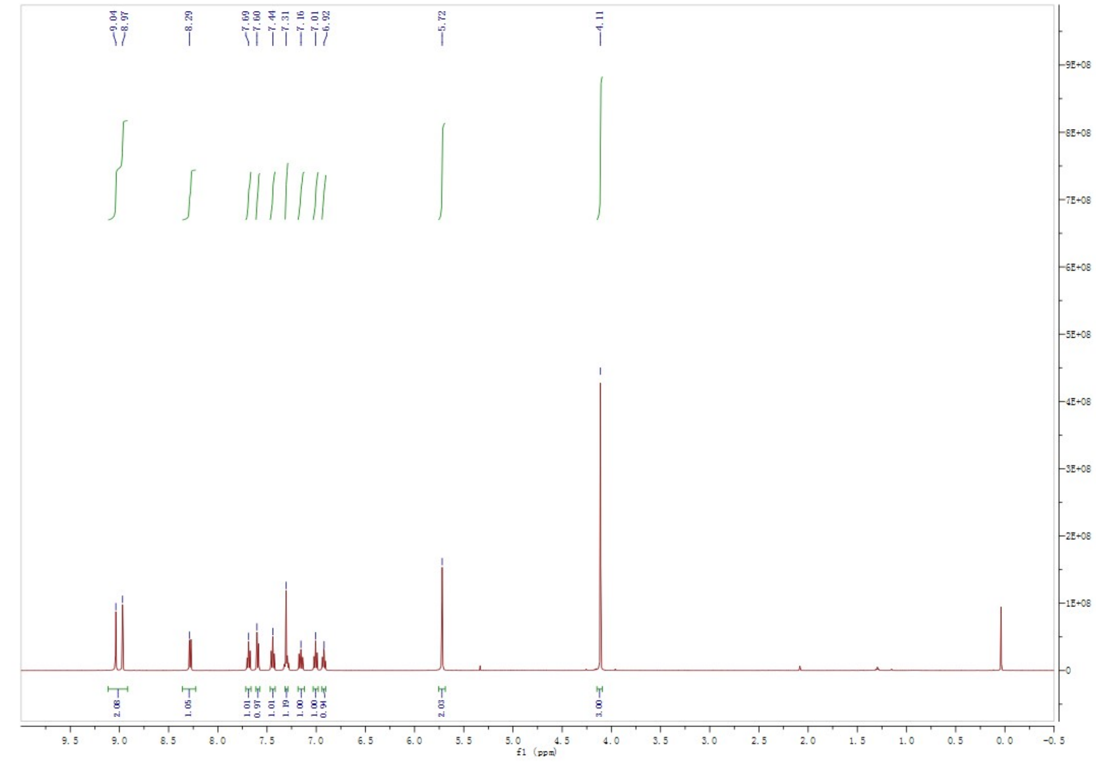
9.04
8.97
8.29
7.69
7.60
7.44
7.31
7.16
7.01
6.92
5.72
4.11
2.08
1.05
1.01
0.97
1.01
1.19
1.00
1.00
0.94
2.03
3.00
f1 (ppm)

C NMR

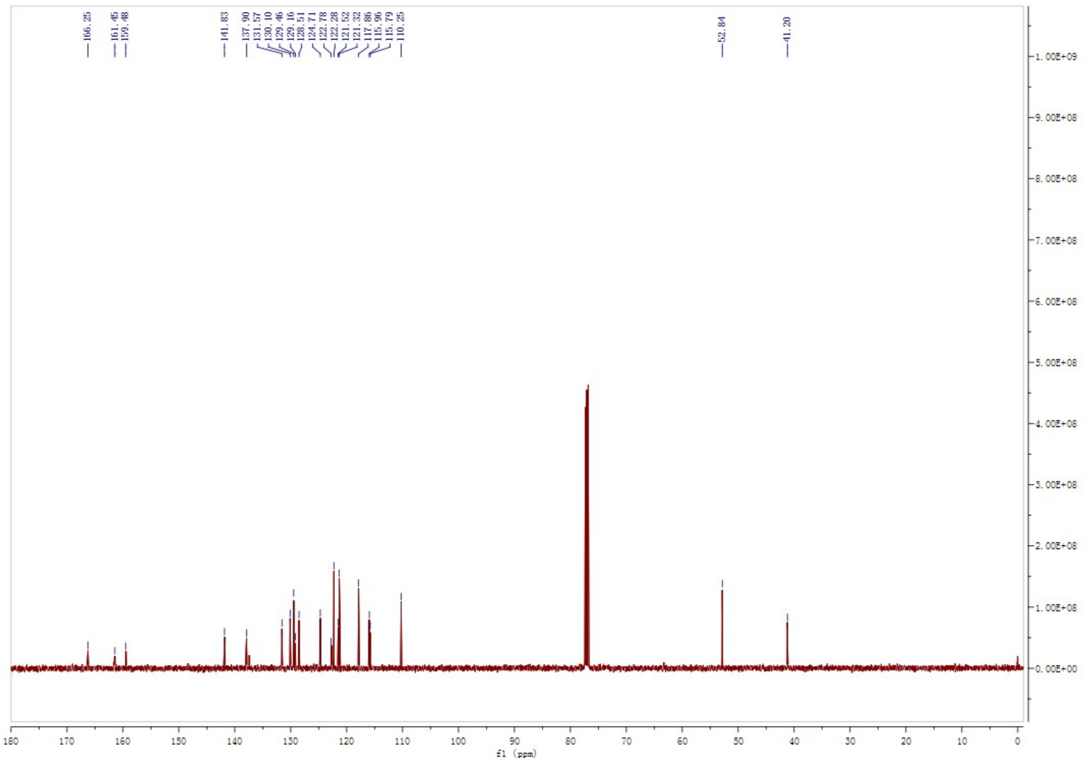

6a

H NMR

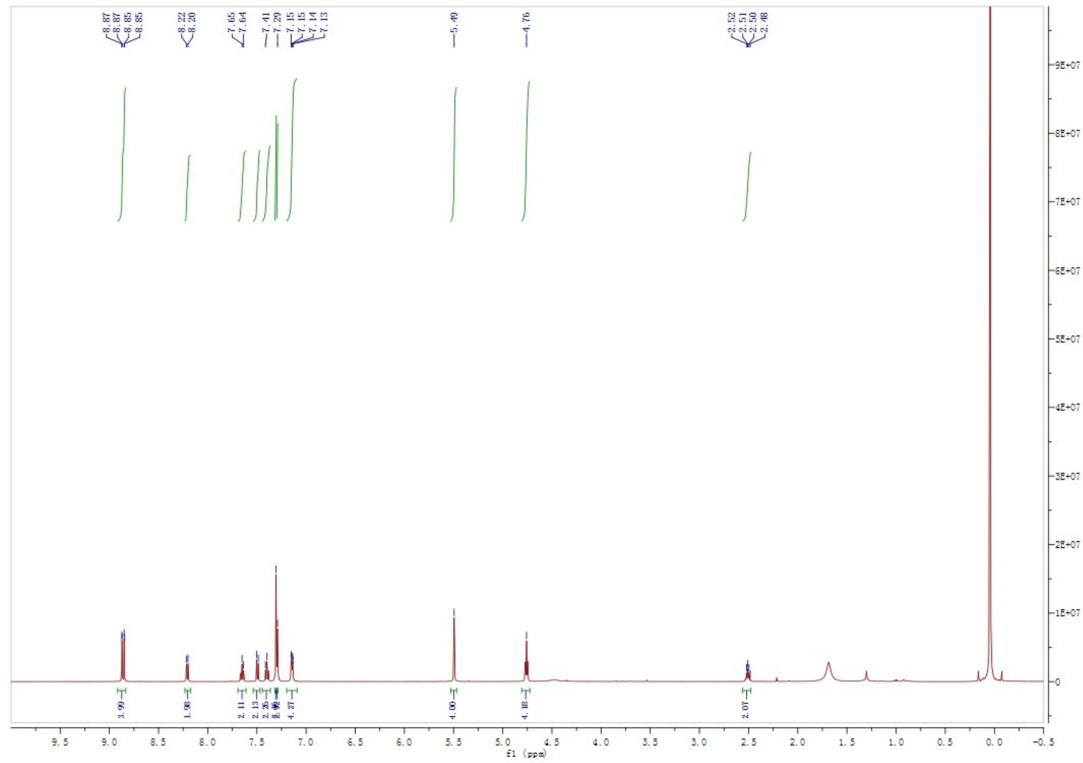

C NMR

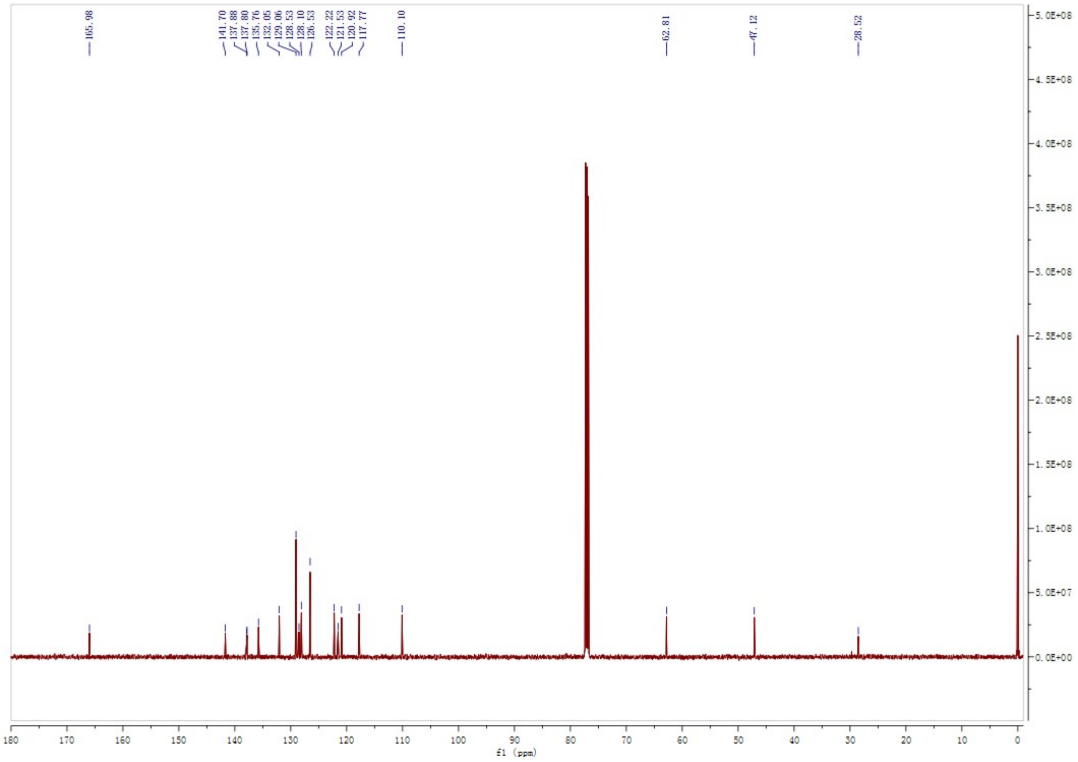
165.98
141.70
137.88
137.80
135.76
132.05
129.06
128.53
128.10
126.53
122.22
121.53
120.92
117.77
110.10
62.81
47.12
28.52
f1 (ppm)

6b

H NMR

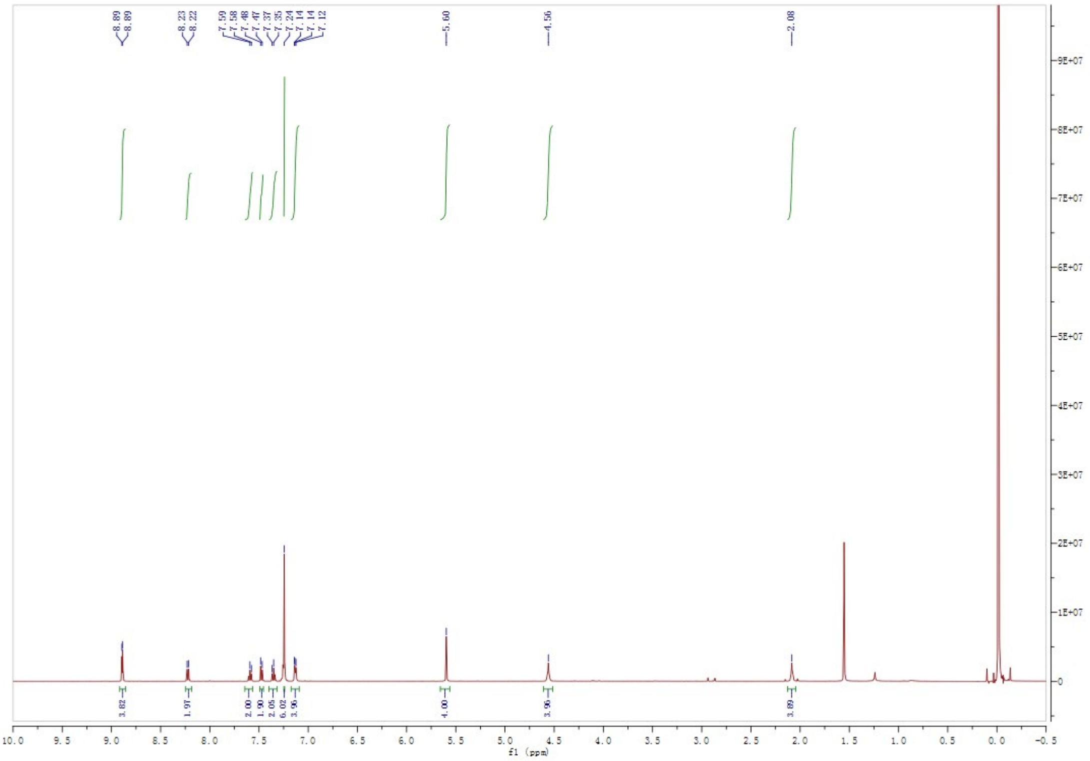
8.89
8.89
8.23
8.22
7.59
7.58
7.48
7.47
7.37
7.35
7.24
7.14
7.12
5.60
4.56
2.08
3.82
1.97
2.00
1.90
2.05
6.02
3.96
4.00
3.96
3.89
f1 (ppm)

C NMR

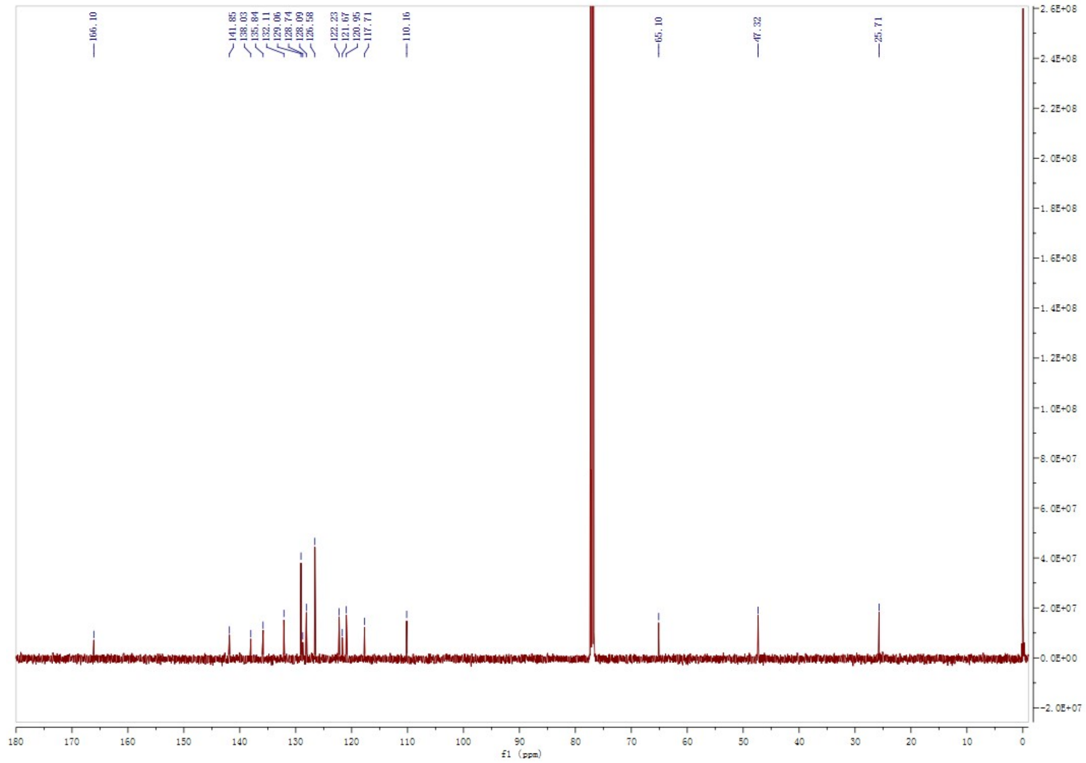
166.10
141.85
138.03
135.84
132.11
129.06
128.74
128.09
126.58
122.23
121.67
120.95
117.71
110.16
65.10
47.32
25.71
f1 (ppm)

6c

H NMR

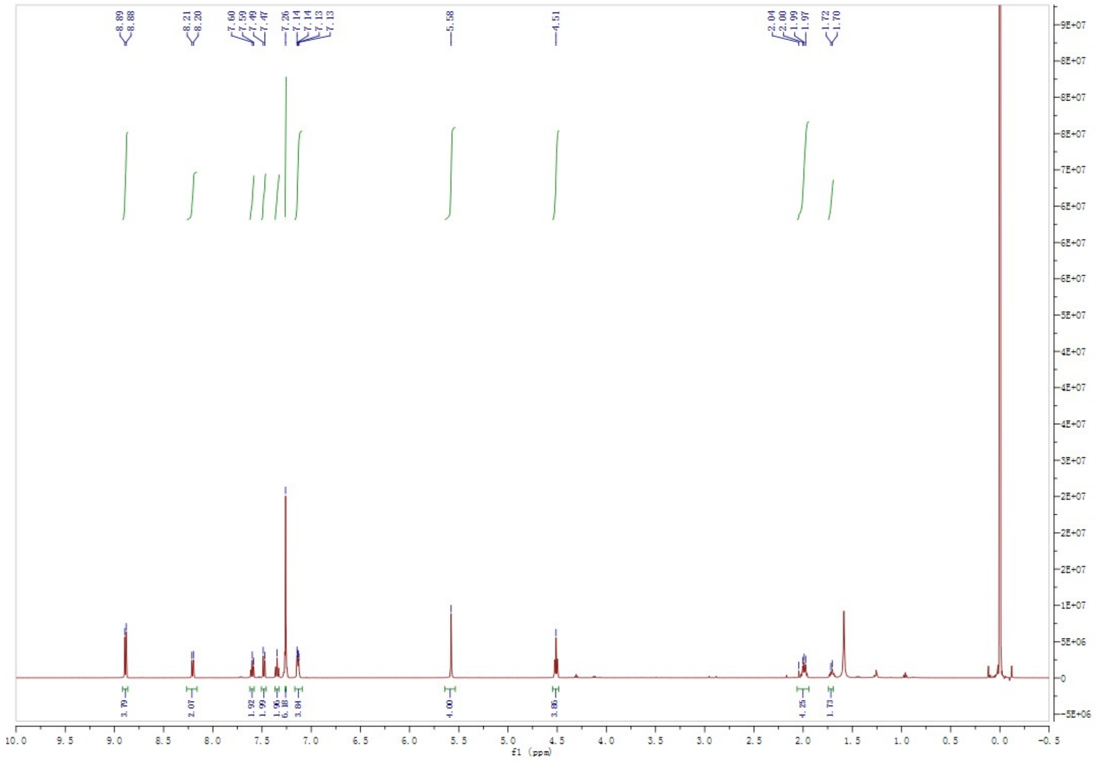
8.89
8.88
8.21
8.20
7.60
7.59
7.49
7.47
7.26
7.14
7.13
7.13
5.58
4.51
2.04
2.00
1.99
1.97
1.72
1.70
3.79
2.07
1.92
1.99
1.96
6.18
3.84
4.00
3.86
4.25
1.73
f1 (ppm)

C NMR

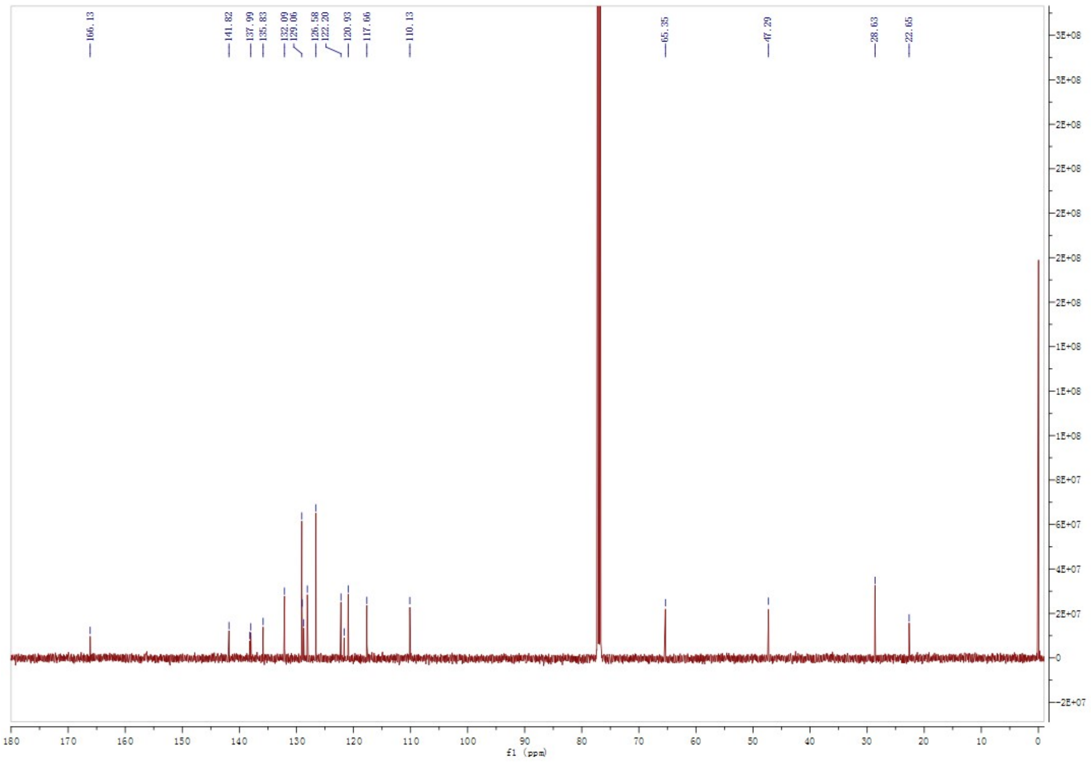
166.13
141.82
137.99
135.83
132.09
129.06
126.58
122.20
120.93
117.66
110.13
65.35
47.29
28.63
22.65
f1 (ppm)

6d

H NMR

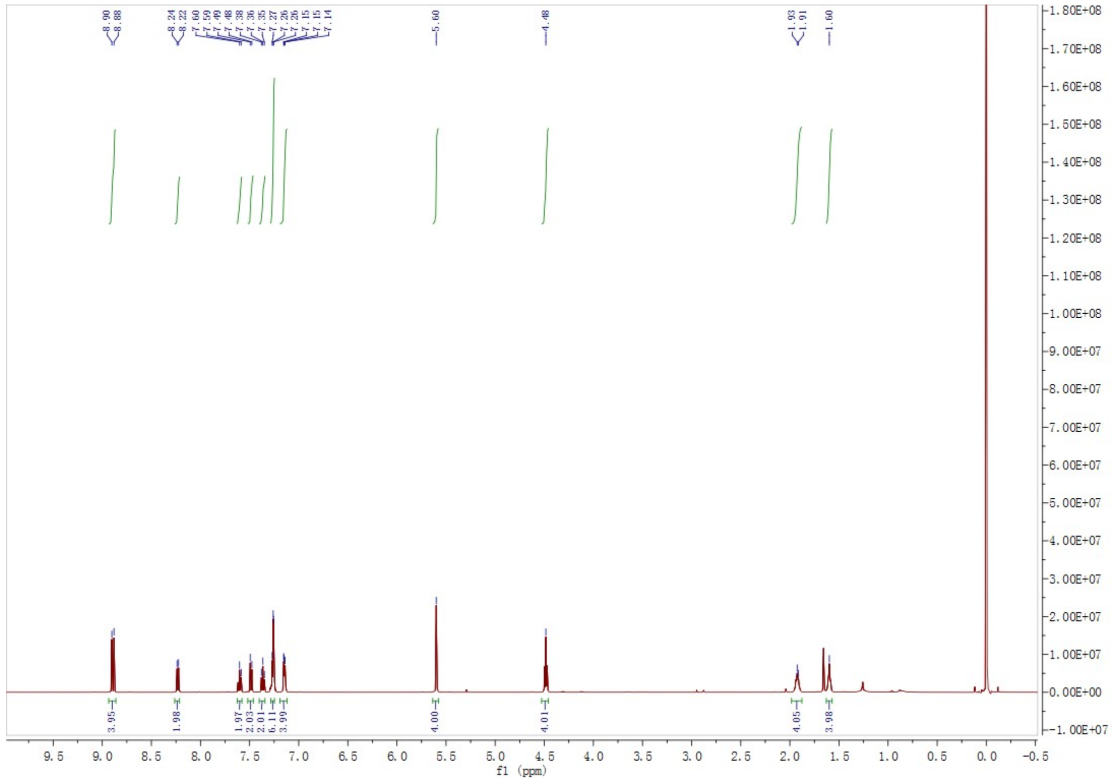
8.90
8.88
8.24
8.22
7.60
7.59
7.49
7.48
7.38
7.36
7.35
7.27
7.26
7.15
7.14
5.60
4.48
1.93
1.91
1.60
3.95
1.98
1.97
2.03
2.01
6.11
3.99
4.00
4.01
4.05
3.98
f1 (ppm)

C NMR

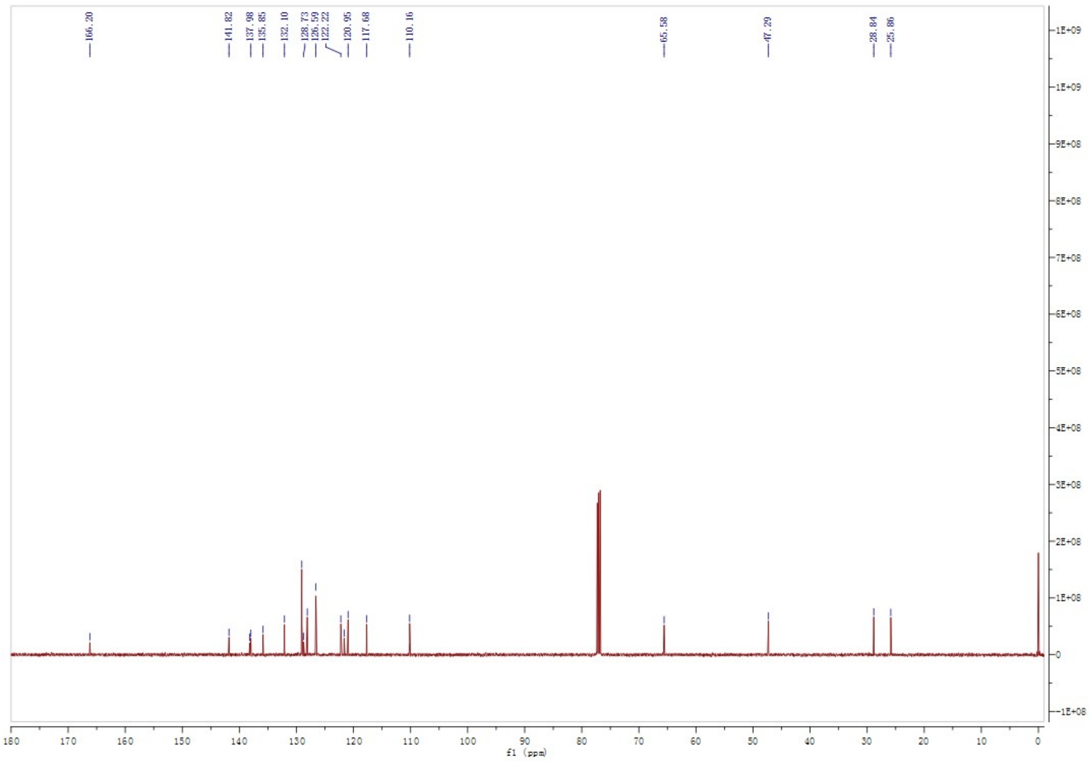
166.20
141.82
137.98
135.85
132.10
128.73
126.59
122.22
120.95
117.68
110.16
65.58
47.29
28.84
25.86
f1 (ppm)

6e

H NMR

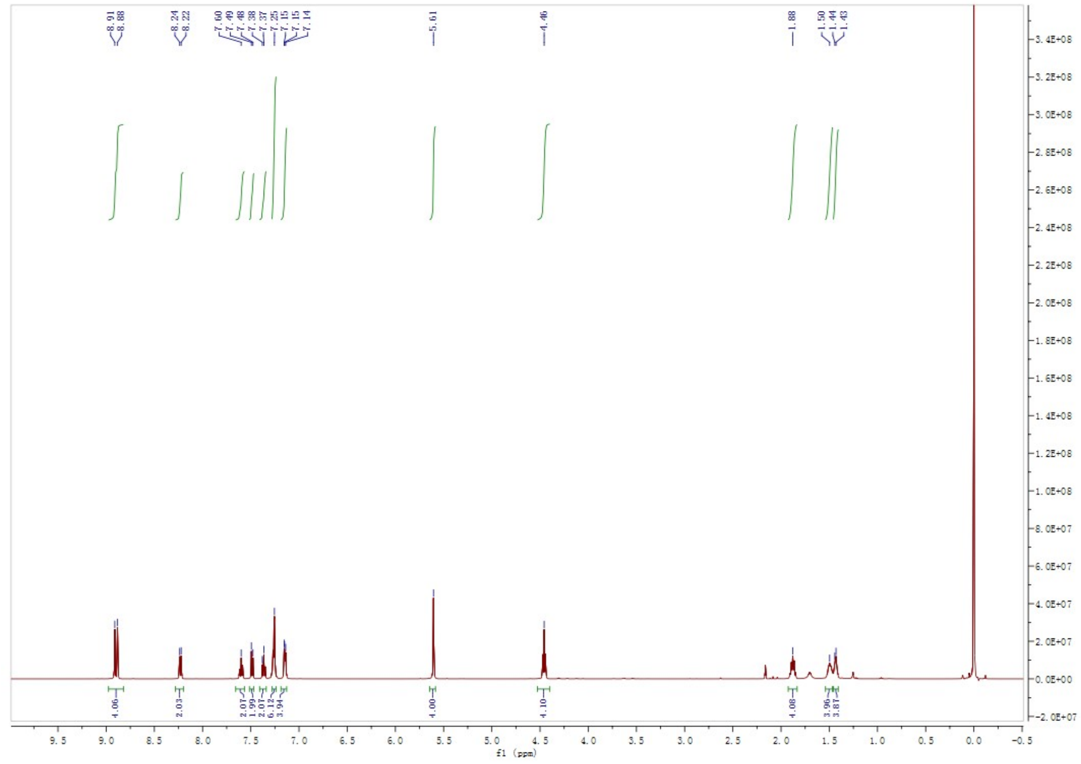
8.91
8.88
8.24
8.22
7.60
7.49
7.48
7.38
7.37
7.25
7.15
7.14
5.61
4.46
1.88
1.50
1.44
1.43
4.06
2.03
2.07
1.99
2.07
6.12
3.94
4.00
4.10
4.08
3.96
3.81
f1 (ppm)

C NMR

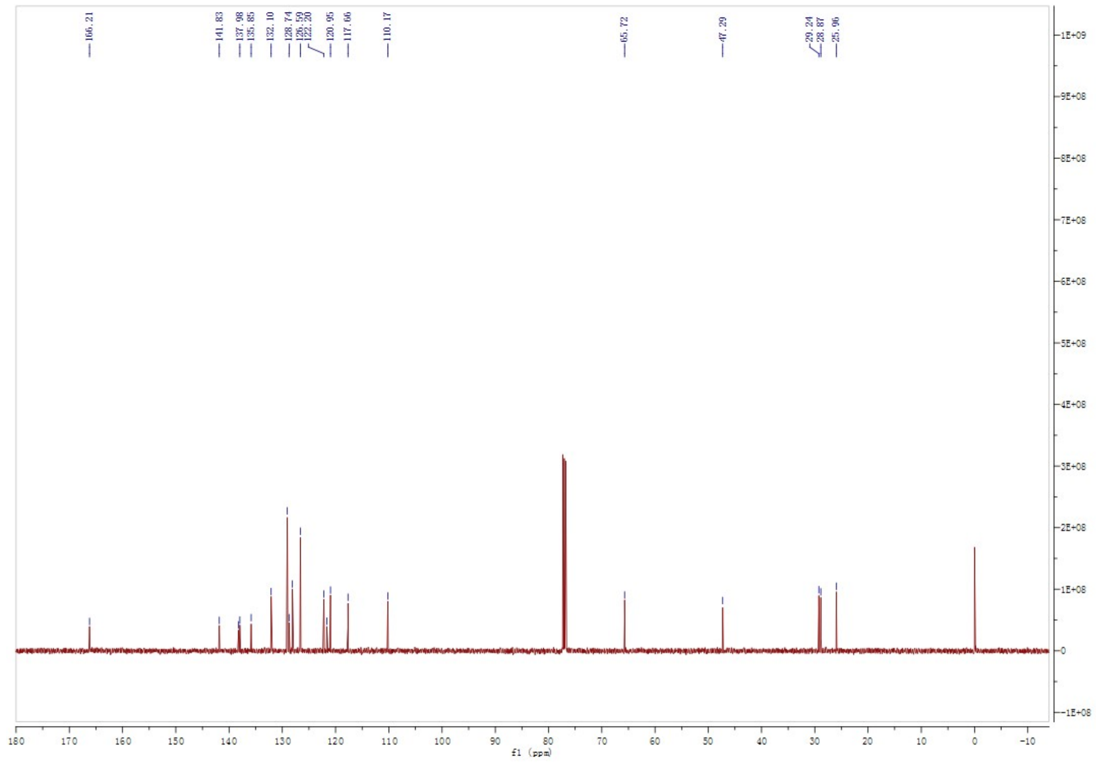
166.21
141.83
137.98
135.85
132.10
128.74
126.59
122.20
120.95
117.66
110.17
65.72
47.29
29.24
28.87
25.96
f1 (ppm)

6f

H NMR

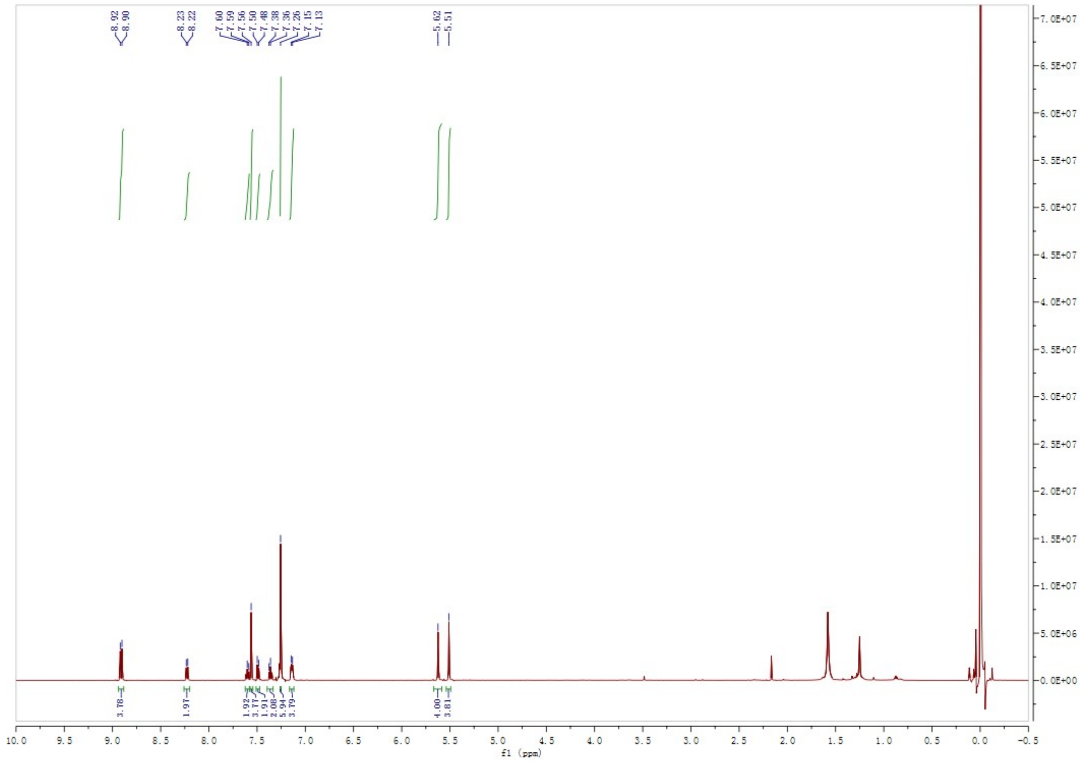
8.92
8.90
8.23
8.22
7.60
7.59
7.56
7.50
7.48
7.38
7.36
7.26
7.15
7.13
5.62
5.51
3.78
1.97
1.92
3.77
1.91
2.08
5.94
3.79
4.00
3.81
f1 (ppm)

C NMR

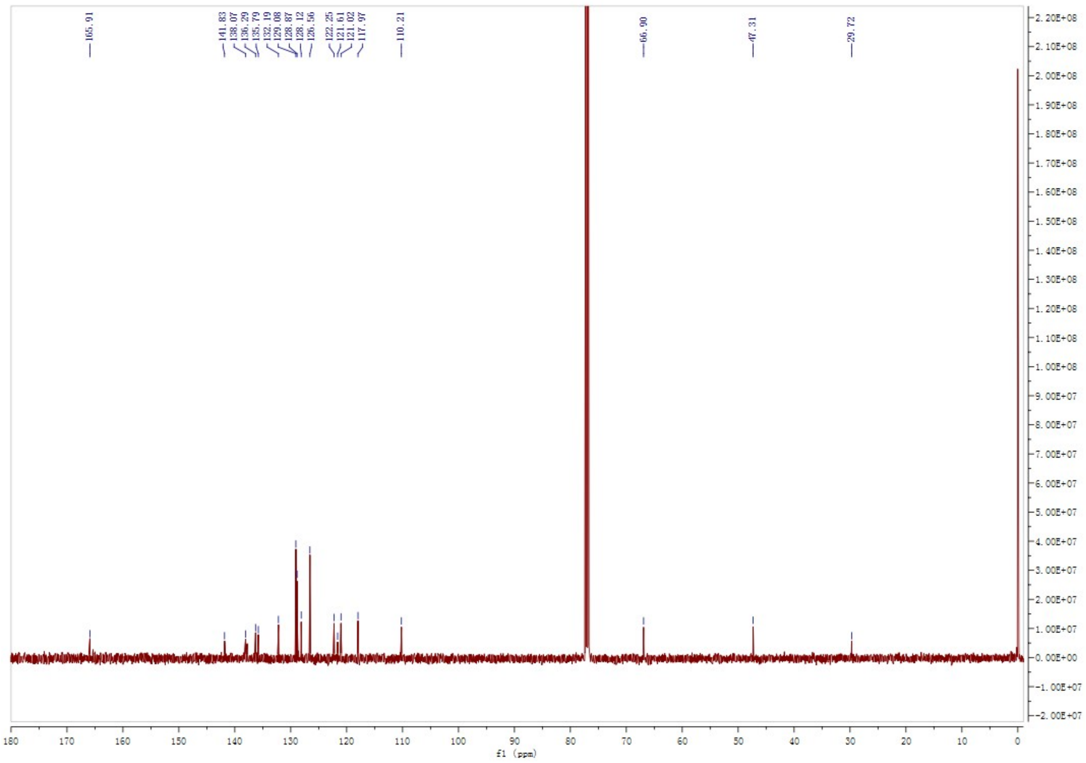
165.91
141.83
138.07
136.29
135.79
132.19
129.08
128.87
128.12
126.56
122.25
121.61
121.02
117.97
110.21
66.90
47.31
29.72
f1 (ppm)

6g

H NMR

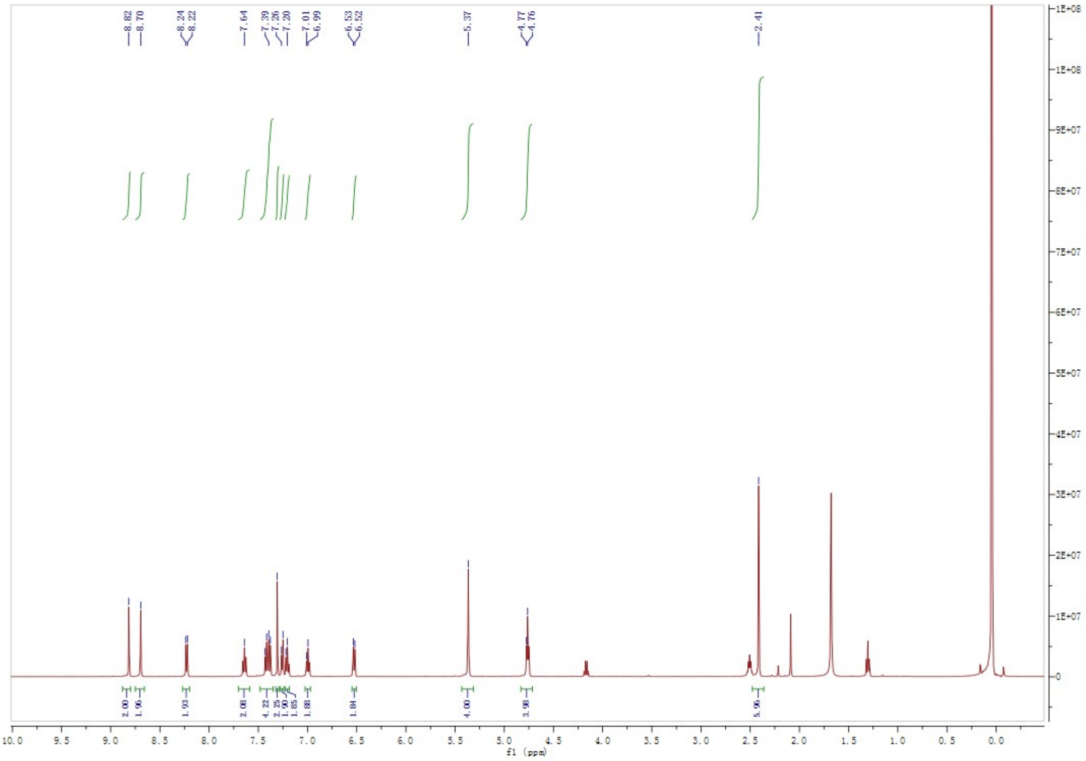
8.82
8.70
8.24
8.22
7.64
7.39
7.26
7.20
7.01
6.99
6.53
6.52
5.37
4.77
4.76
2.41
2.00
1.96
1.93
2.08
4.22
2.25
1.90
1.85
1.88
1.84
4.00
3.98
5.96
f1 (ppm)

C NMR

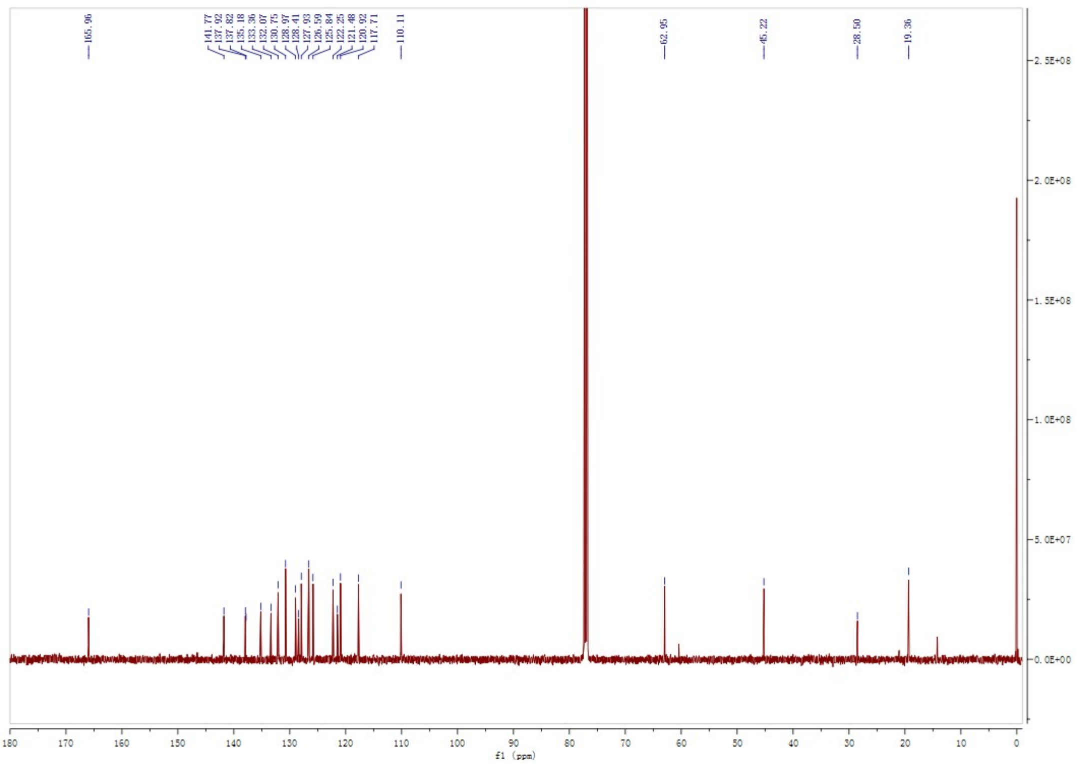
165.96
141.77
137.92
137.82
135.18
133.36
132.07
130.75
128.97
128.41
127.93
126.59
125.84
122.25
121.48
120.92
117.71
110.11
62.95
45.22
28.50
19.36
f1 (ppm)

6h

H NMR

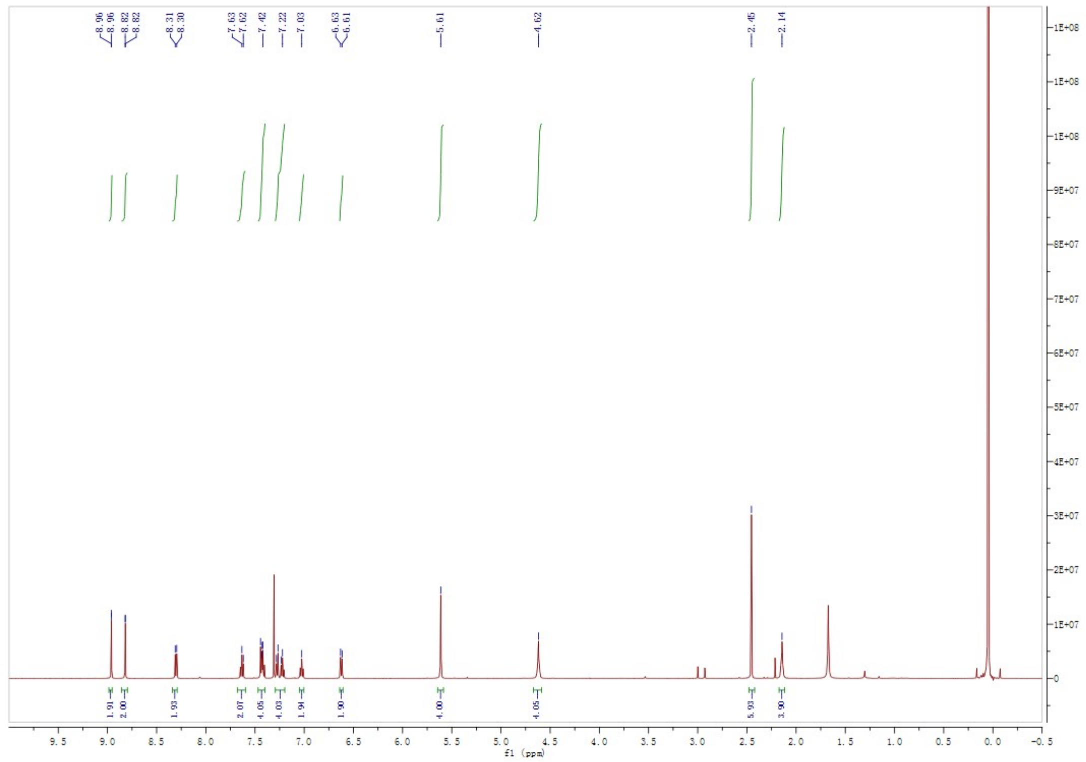
8.96
8.96
8.82
8.82
8.31
8.30
7.63
7.62
7.42
7.22
7.03
6.63
6.61
5.61
4.62
2.45
2.14
1.91
2.00
1.93
2.07
4.05
4.03
1.94
1.90
4.00
4.05
5.93
3.90
f1 (ppm)

C NMR

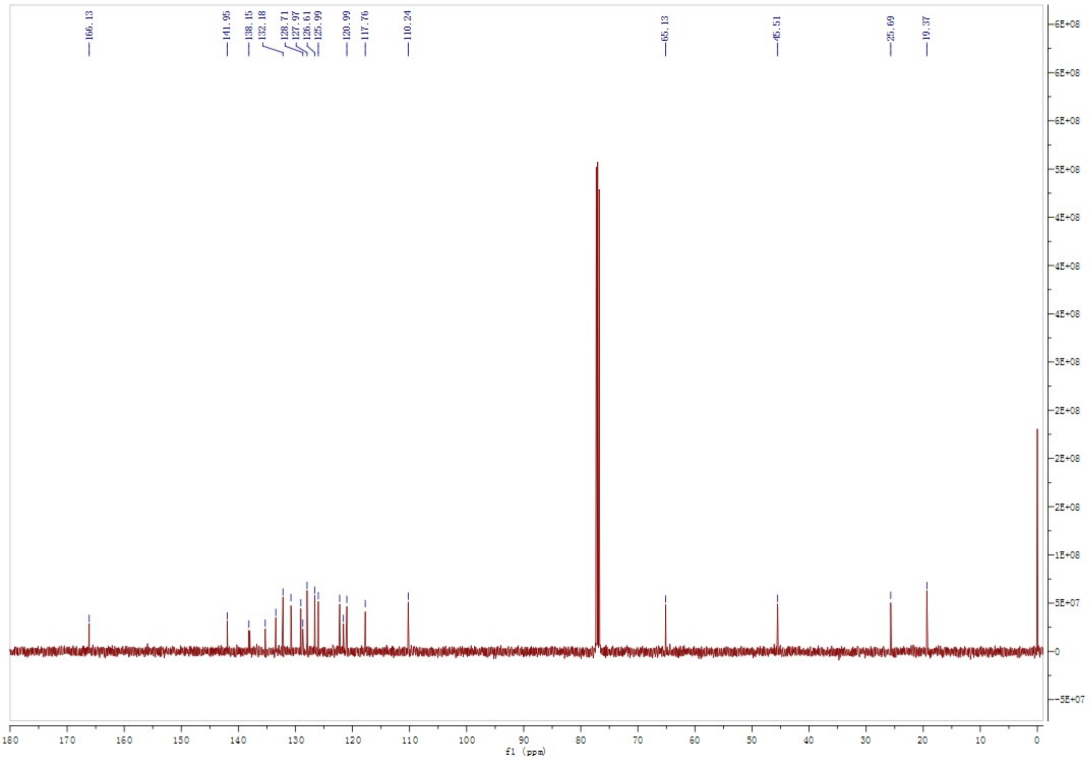
166.13
141.95
138.15
132.18
128.71
127.97
126.61
125.99
120.99
117.76
110.24
65.13
45.51
25.69
19.37
f1 (ppm)

6i

H NMR

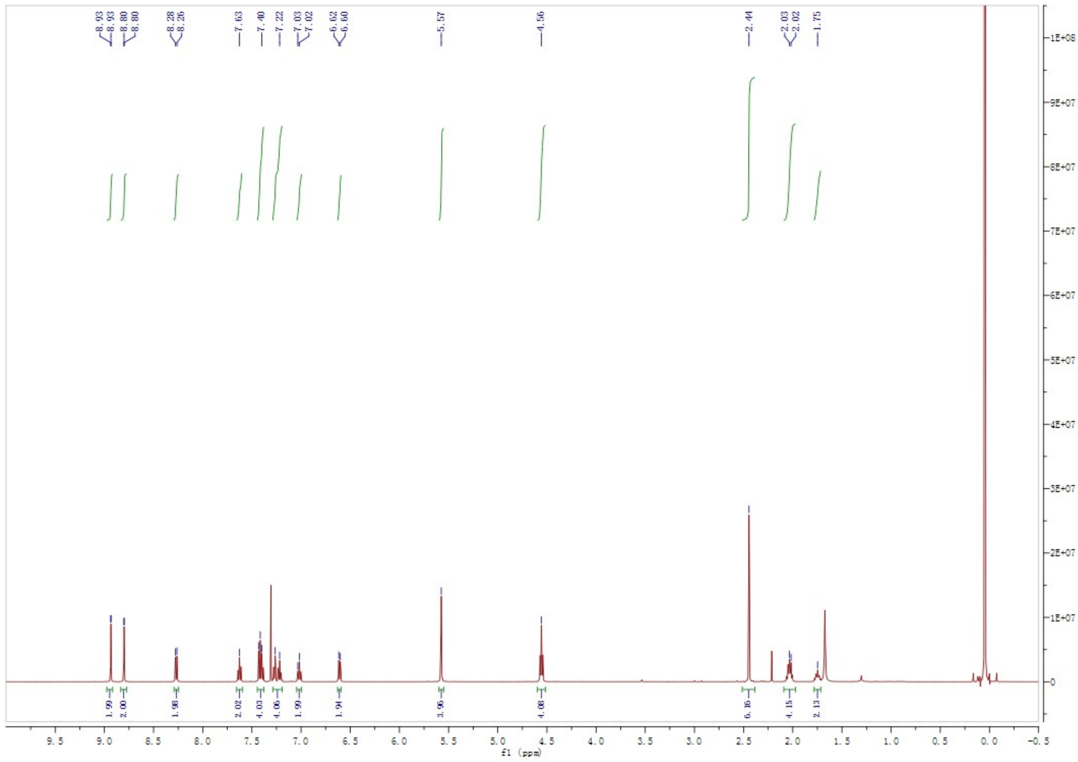
8.93
8.93
8.80
8.80
8.28
8.26
7.63
7.40
7.22
7.03
7.02
6.62
6.60
5.57
4.56
2.44
2.03
2.02
1.75
1.99
2.00
1.98
2.02
4.03
4.06
1.99
1.94
3.96
4.08
6.16
4.15
2.13
f1 (ppm)

C NMR

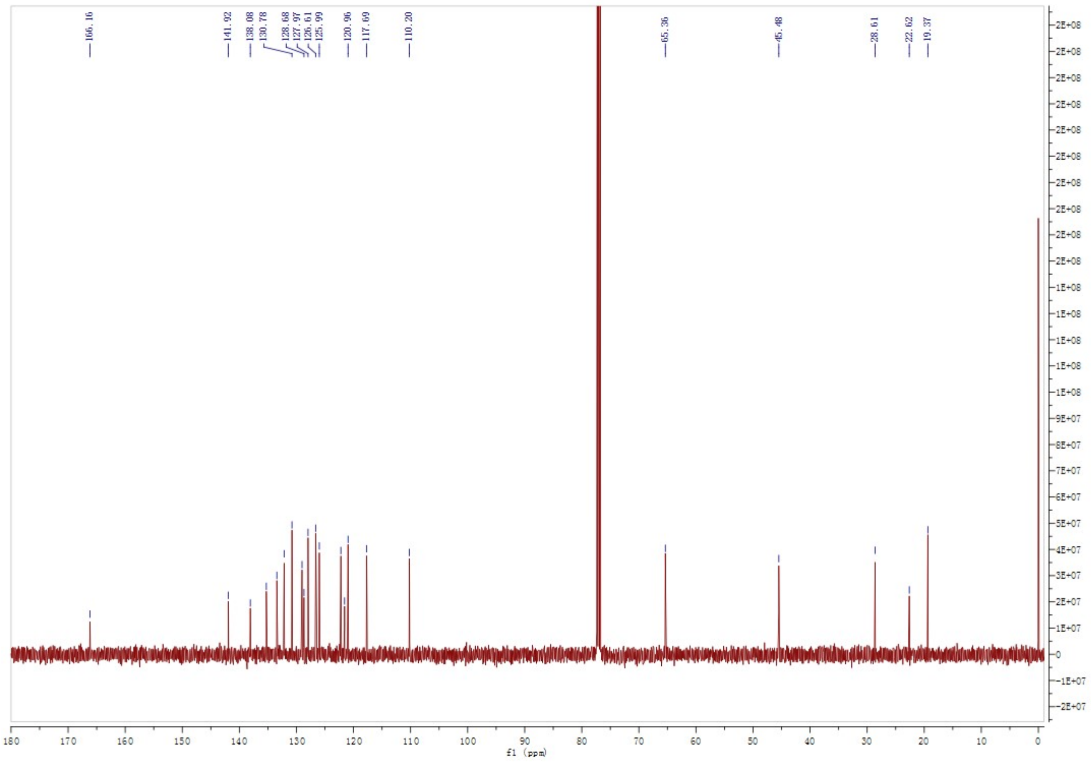
166.16
141.92
138.08
130.78
128.68
127.97
126.61
125.99
120.96
117.69
110.20
65.36
45.48
28.61
22.62
19.37
f1 (ppm)

6j

H NMR

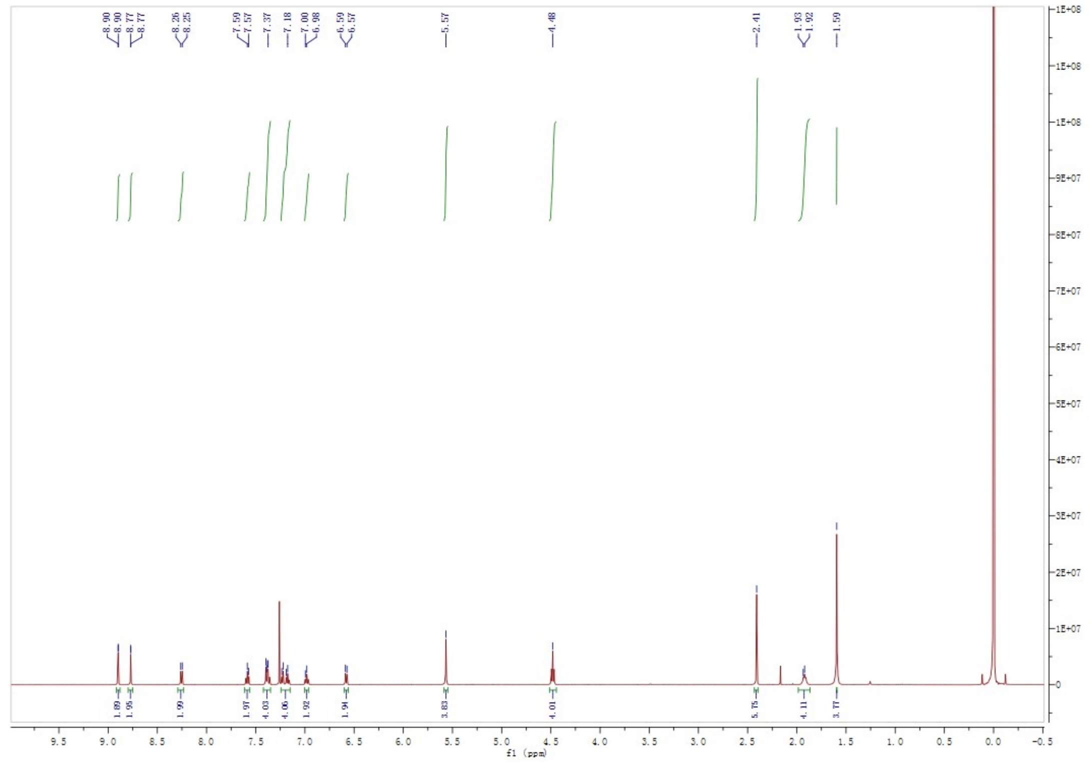
8.90
8.90
8.77
8.77
8.26
8.25
7.59
7.57
7.37
7.18
7.00
6.98
6.59
6.57
5.57
4.48
2.41
1.93
1.92
1.59
1.89
1.95
1.99
1.97
4.03
4.06
1.92
1.94
3.83
4.01
5.75
4.11
3.77
f1 (ppm)

C NMR

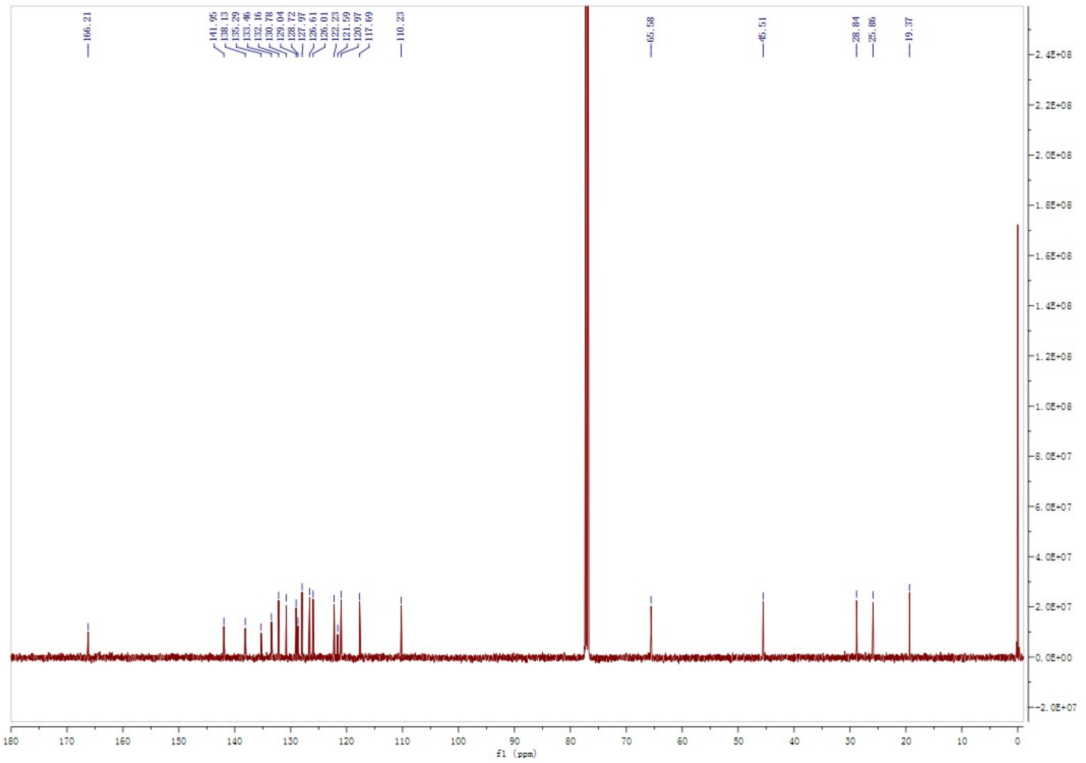
166.21
141.95
138.13
135.29
133.46
132.16
130.78
129.04
128.72
127.97
126.61
126.01
122.23
121.59
120.97
117.69
110.23
65.58
45.51
28.84
25.86
19.37
f1 (ppm)

6k

H NMR

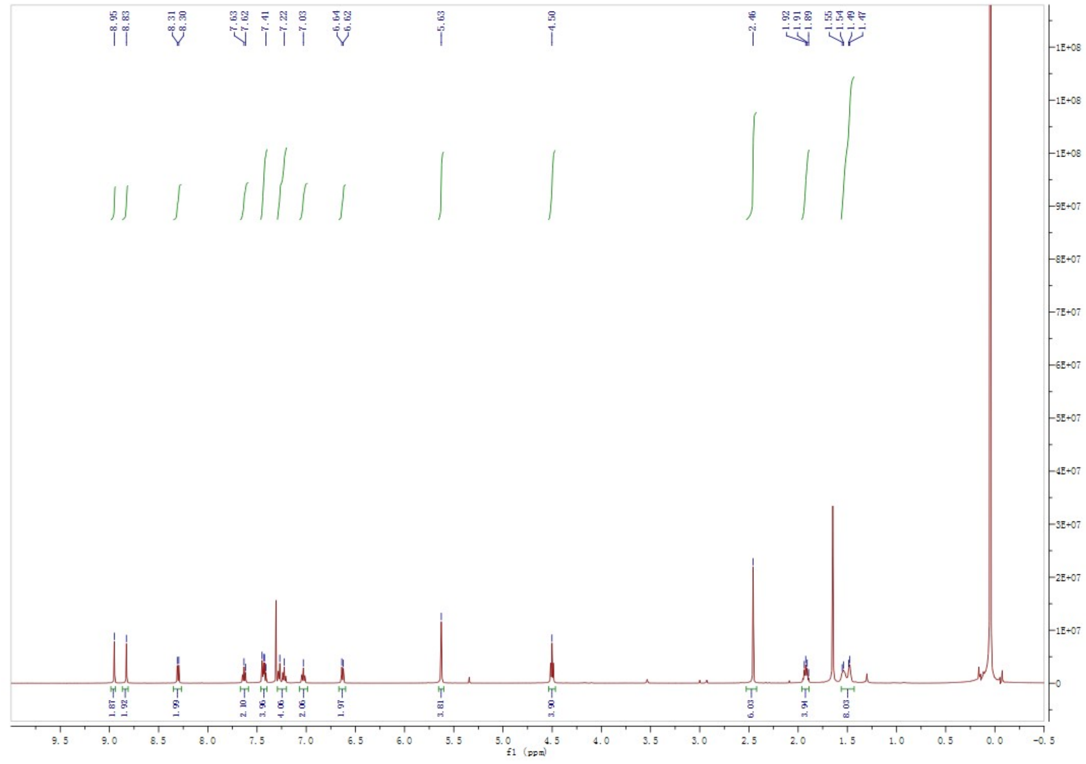
8.95
8.83
8.31
8.30
7.63
7.62
7.41
7.22
7.03
6.64
6.62
5.63
4.50
2.46
1.92
1.91
1.89
1.55
1.54
1.49
1.47
1.87
1.92
1.99
2.10
3.96
4.06
2.06
1.97
3.81
3.90
6.03
3.94
8.03
f1 (ppm)

C NMR

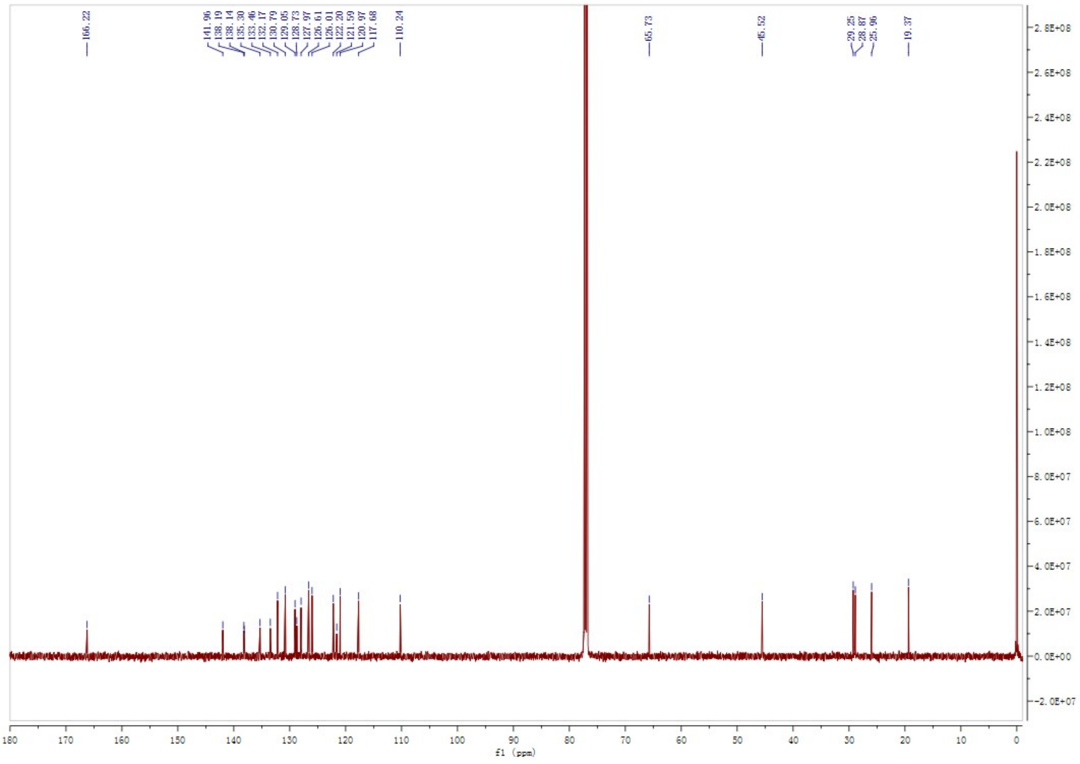
166.22
141.96
138.19
138.14
135.30
133.46
132.17
130.79
129.05
128.73
127.97
126.61
126.01
122.20
121.59
120.97
117.68
110.24
65.73
45.52
29.25
28.87
25.96
19.37
f1 (ppm)

6l

H NMR

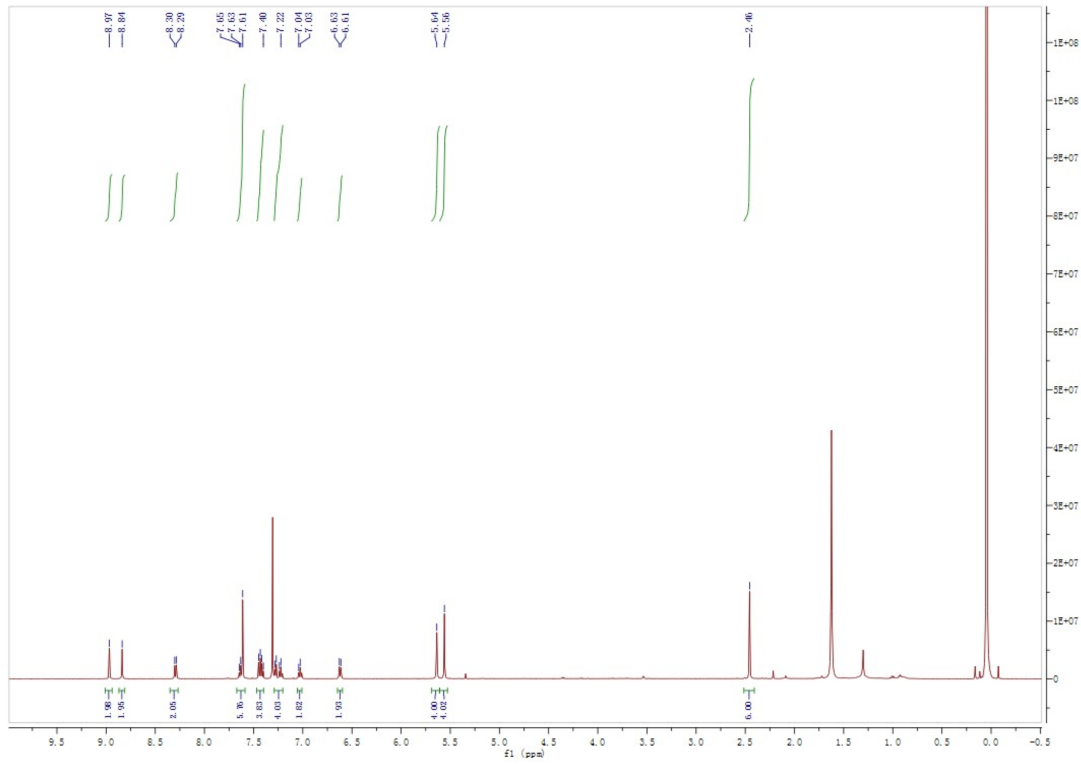
8.97
8.84
8.30
8.29
7.65
7.63
7.61
7.40
7.22
7.04
7.03
6.63
6.61
5.64
5.56
2.46
1.98
1.95
2.05
5.76
3.83
4.03
1.82
1.93
4.00
4.02
6.00
f1 (ppm)

C NMR

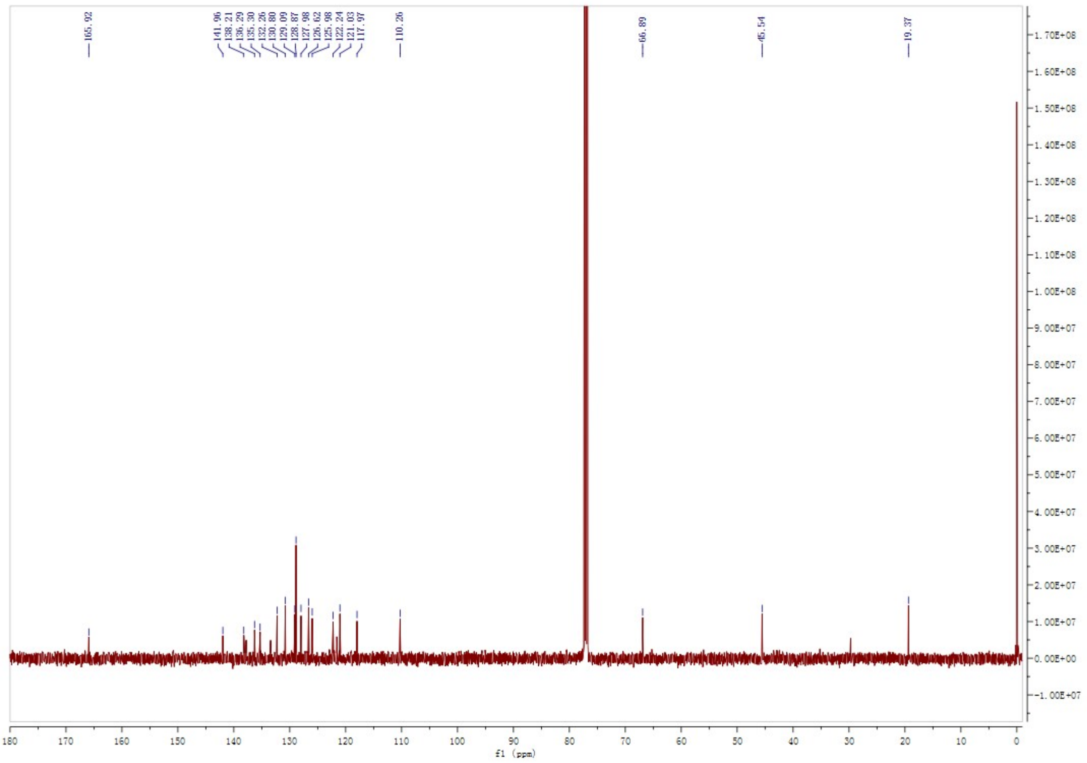
165.92
141.96
138.21
136.29
135.30
132.26
130.80
129.09
128.87
127.98
126.62
125.98
122.24
121.03
117.97
110.26
66.89
45.54
19.37
f1 (ppm)

6m

H NMR

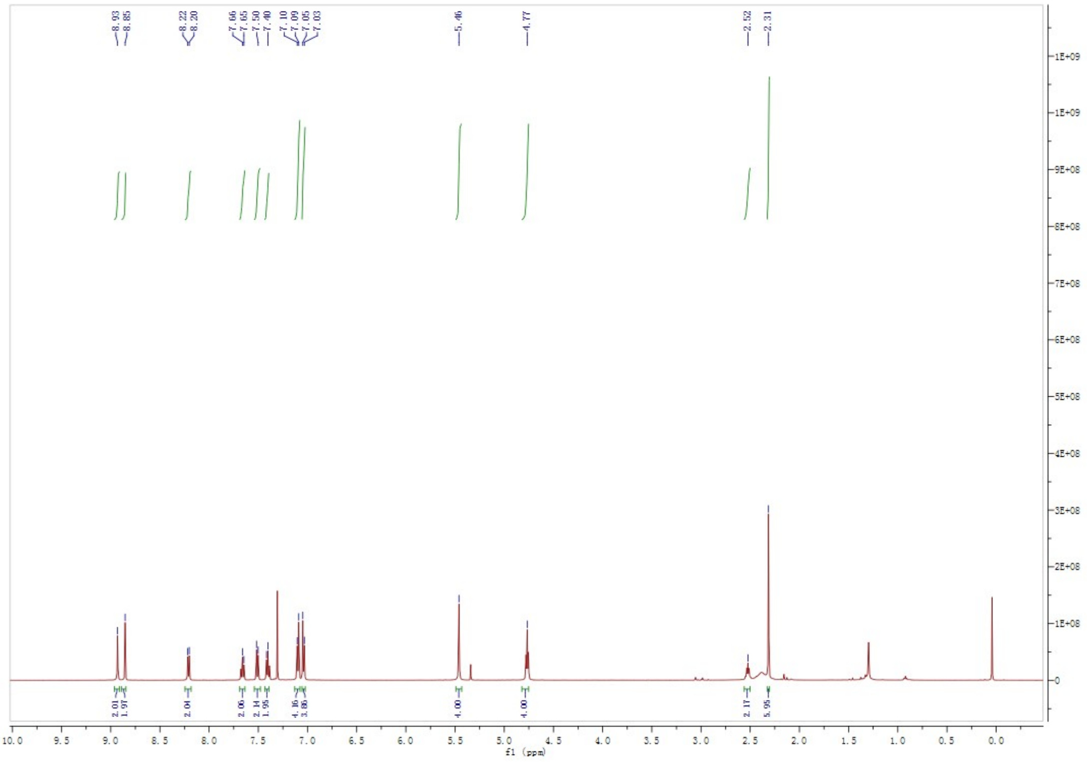
8.93
8.85
8.22
8.20
7.66
7.65
7.50
7.40
7.10
7.09
7.05
7.03
5.46
4.77
2.52
2.31
2.01
1.97
2.04
2.06
2.14
1.95
4.16
3.86
4.00
4.00
2.17
5.95
f1 (ppm)

C NMR

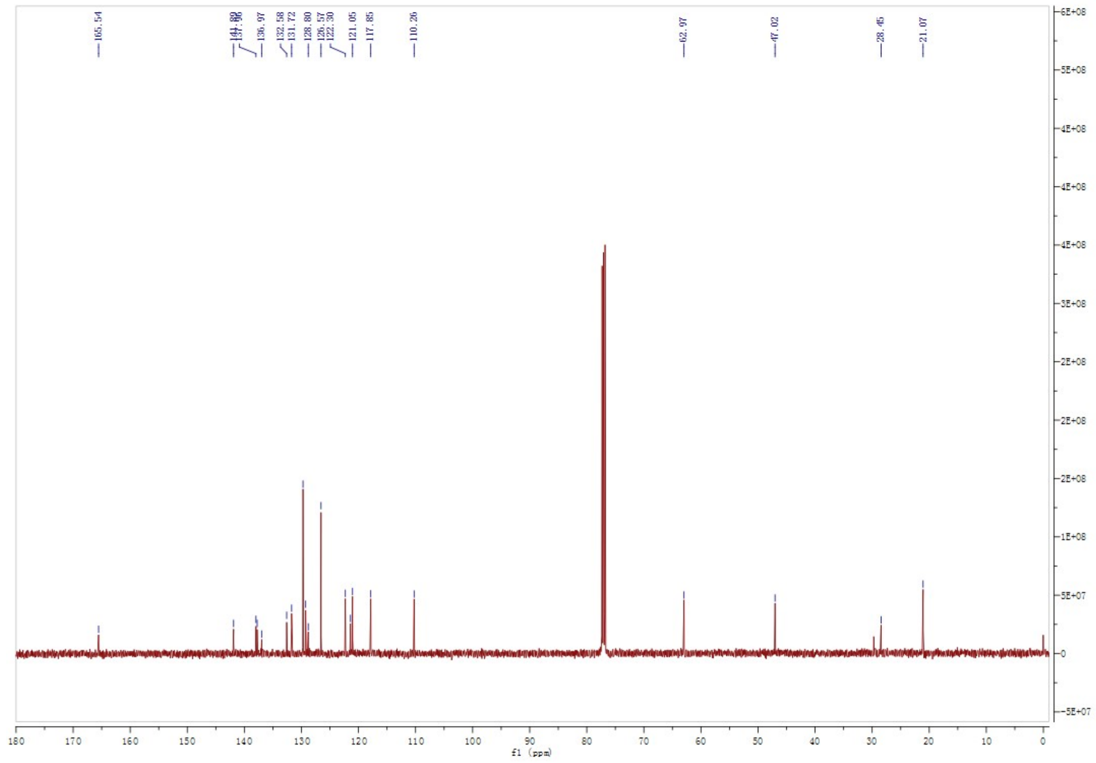
165.54
137.96
136.97
132.58
131.72
128.80
126.57
122.30
121.05
117.85
110.26
62.97
47.02
28.45
21.07
f1 (ppm)

6n

H NMR

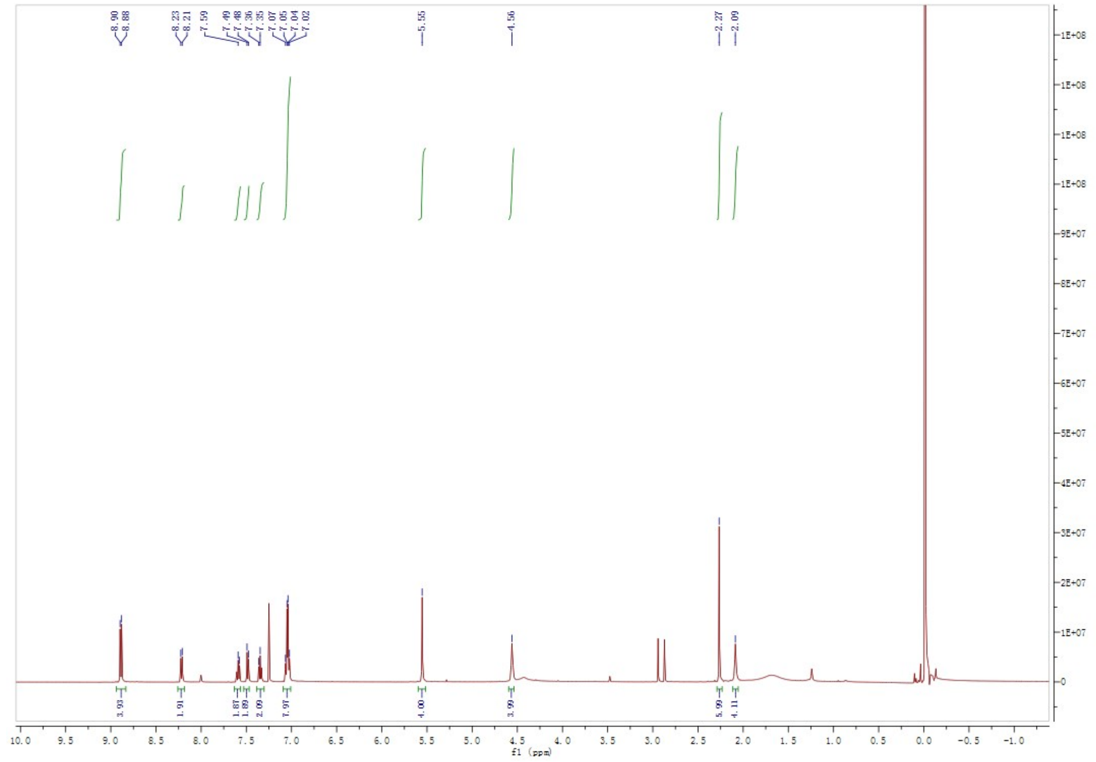
8.90
8.88
8.23
8.21
7.59
7.49
7.48
7.36
7.35
7.07
7.05
7.04
7.02
5.55
4.56
2.27
2.09
3.93
1.91
1.87
1.89
2.09
7.97
4.00
3.99
5.99
4.11
f1 (ppm)

C NMR

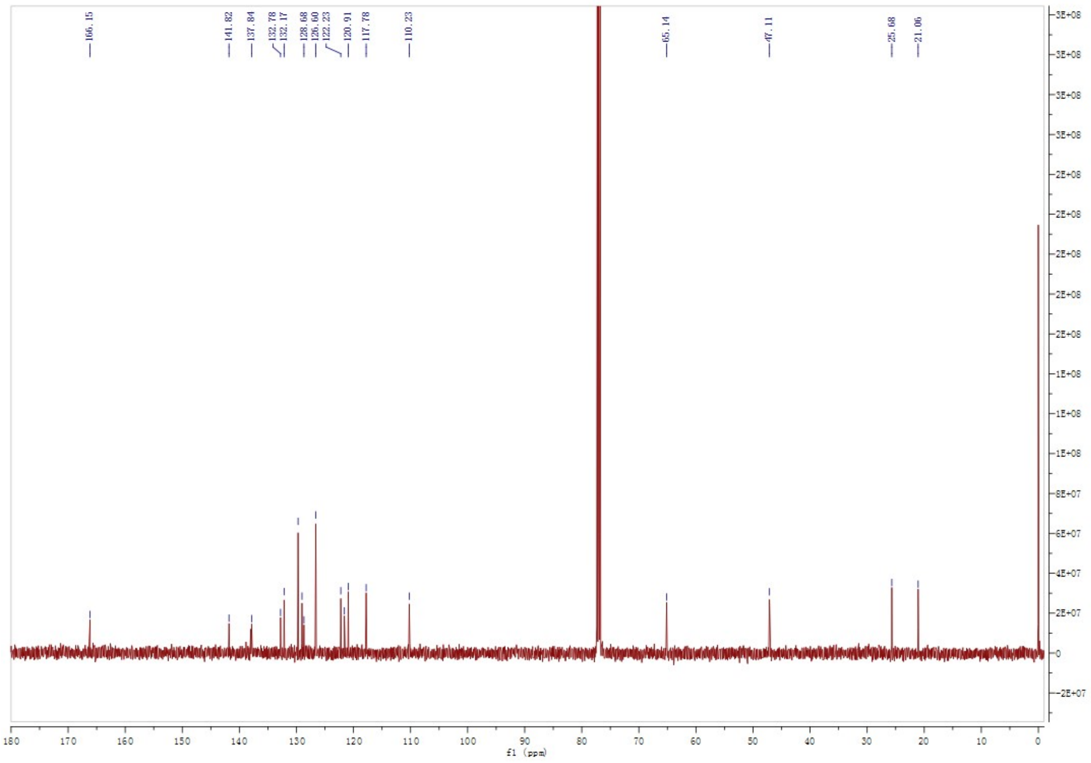
166.15
141.82
137.84
132.78
132.17
128.68
126.60
122.23
120.91
117.78
110.23
65.14
47.11
25.68
21.06
f1 (ppm)

6o

H NMR

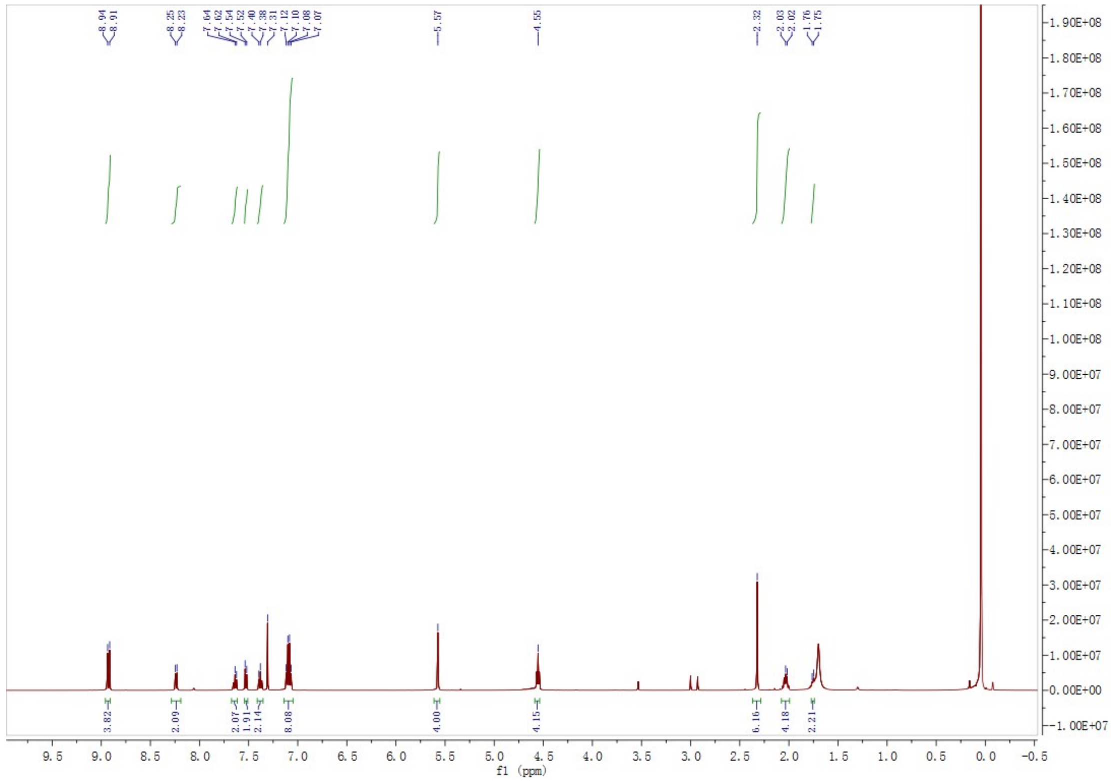
8.94
8.91
8.25
8.23
7.64
7.62
7.54
7.52
7.40
7.38
7.31
7.12
7.10
7.08
7.07
5.57
4.55
2.32
2.03
2.02
1.76
1.75
3.82
2.09
2.07
1.91
2.14
8.08
4.00
4.15
6.16
4.18
2.21
f1 (ppm)

C NMR

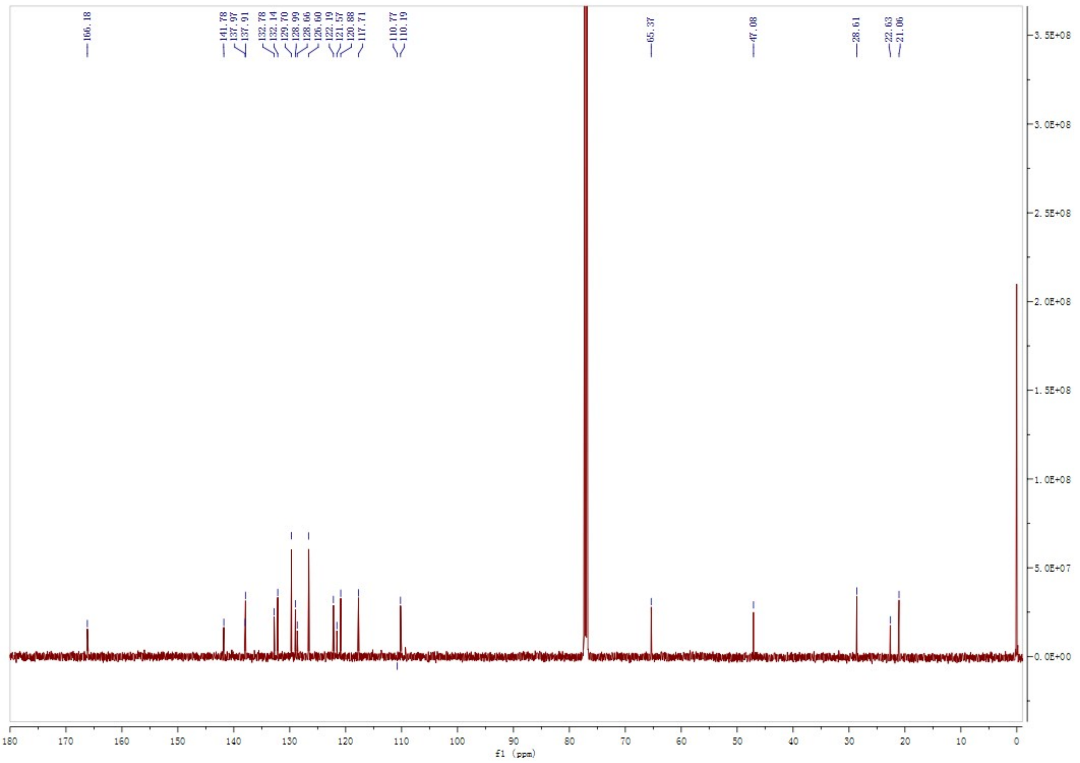
166.18
141.78
137.97
137.91
132.78
132.14
129.70
128.99
128.66
126.60
122.19
121.57
120.88
117.71
110.77
110.19
65.37
47.08
28.61
22.63
21.06
f1 (ppm)

6p

H NMR

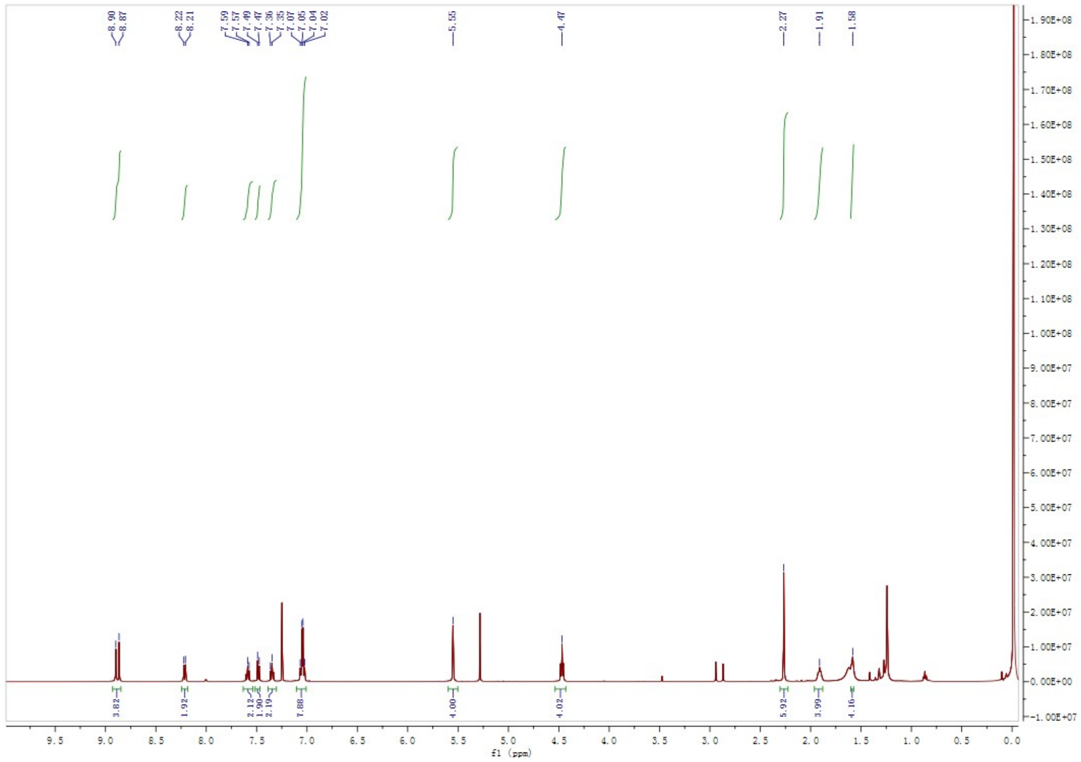
8.90
8.87
8.22
8.21
7.59
7.57
7.49
7.47
7.36
7.35
7.07
7.05
7.04
7.02
5.55
4.47
2.27
1.91
1.58
3.82
1.92
2.12
1.90
2.19
7.88
4.00
4.02
5.92
3.99
4.16
f1 (ppm)

C NMR

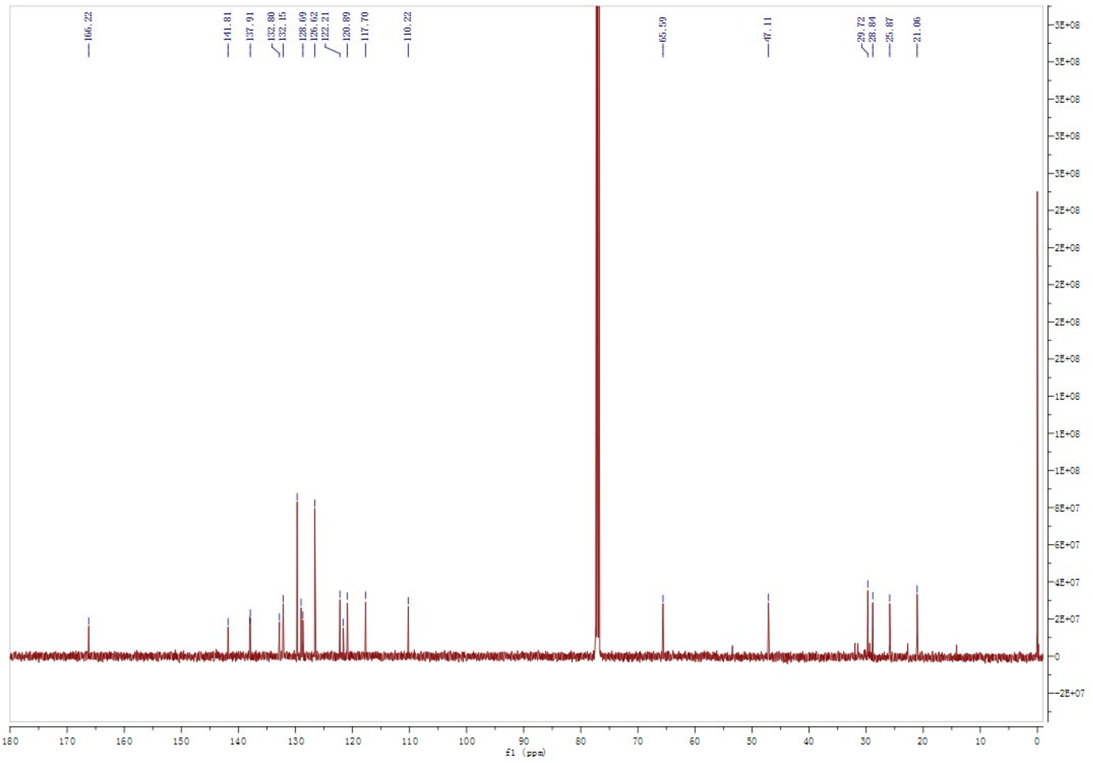
166.22
141.81
137.91
132.80
132.15
128.69
126.62
122.21
120.89
117.70
110.22
65.59
47.11
29.72
28.84
25.87
21.06
f1 (ppm)

6q

H NMR

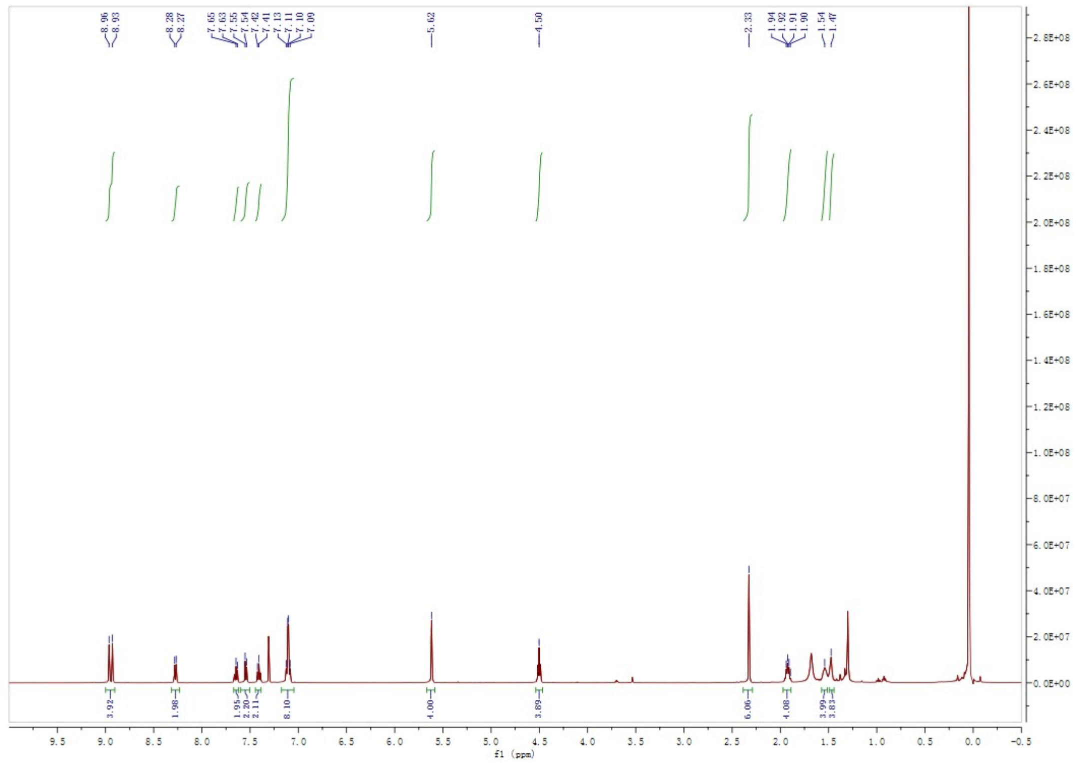
8.96
8.93
8.28
8.27
7.65
7.63
7.55
7.54
7.42
7.41
7.13
7.11
7.10
7.09
5.62
4.50
2.33
1.94
1.92
1.91
1.90
1.54
1.47
3.92
1.98
1.95
2.20
2.11
8.10
4.00
3.89
6.06
4.08
3.99
3.83
f1 (ppm)

C NMR

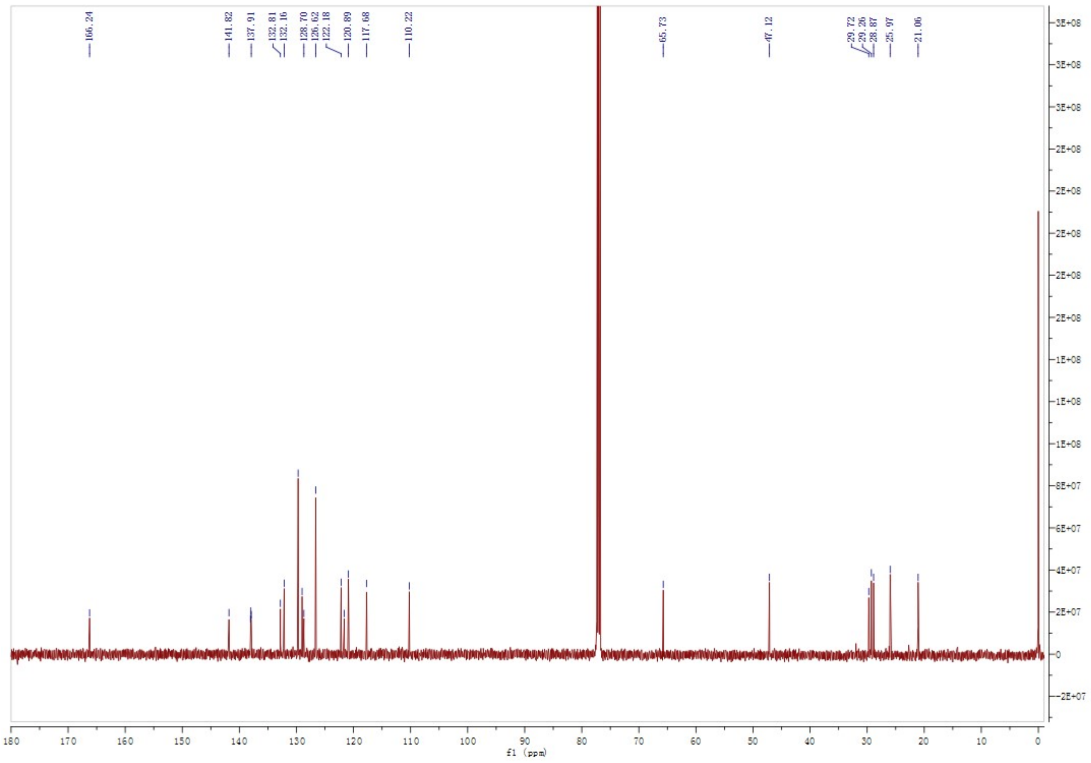
166.24
141.82
137.91
132.81
132.16
128.70
126.62
122.18
120.89
117.68
110.22
65.73
47.12
29.72
29.26
28.87
25.97
21.06
f1 (ppm)

6r

H NMR

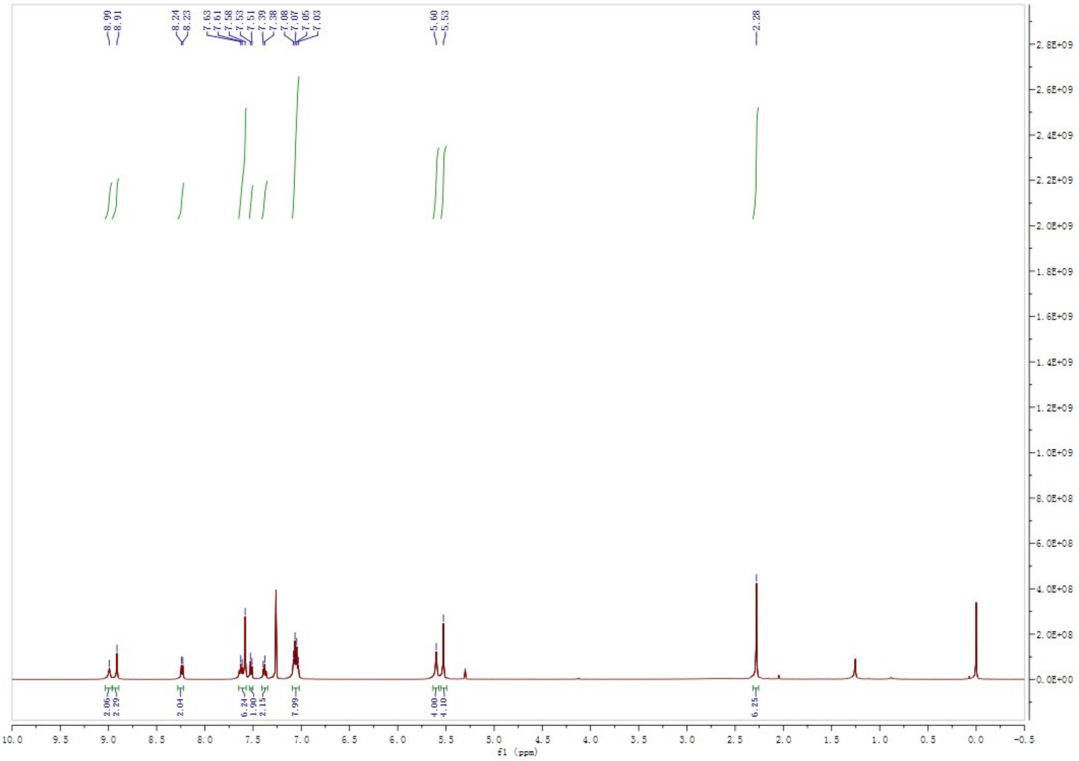
8.99
8.91
8.24
8.23
7.63
7.61
7.58
7.53
7.51
7.39
7.38
7.08
7.07
7.05
7.03
5.60
5.53
2.28
2.06
2.29
2.04
6.24
1.90
2.15
7.99
4.00
4.10
6.25
f1 (ppm)

C NMR

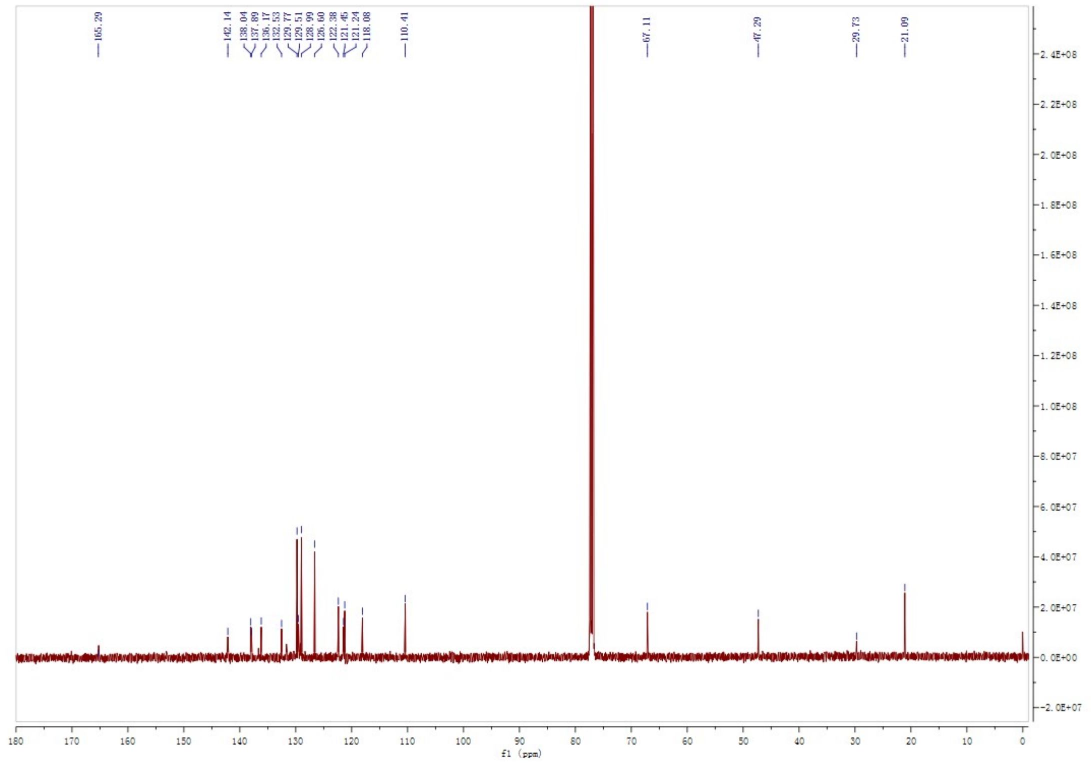
165.29
142.14
138.04
137.89
136.17
132.53
129.77
129.51
128.99
126.60
122.38
121.46
121.24
118.08
110.41
67.11
47.29
29.73
21.09
f1 (ppm)

6s

H NMR

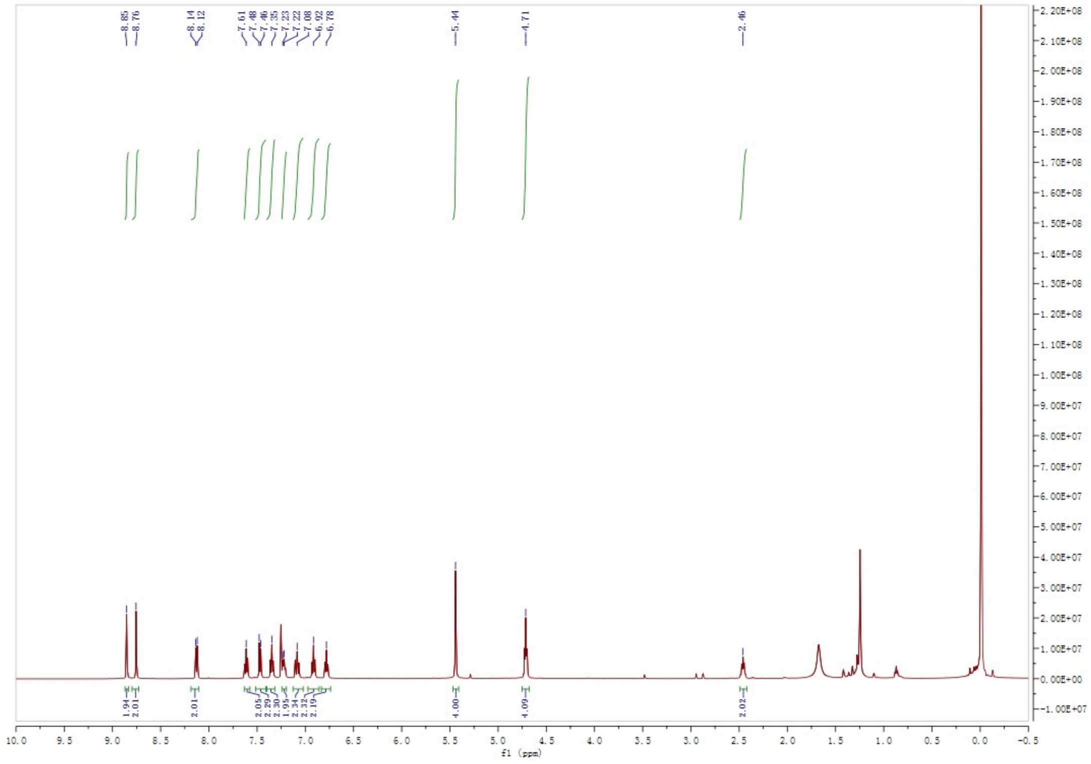
8.85
8.76
8.14
8.12
7.61
7.48
7.46
7.35
7.23
7.22
7.08
6.92
6.78
5.44
4.71
2.46
1.94
2.01
2.01
2.05
2.29
2.30
1.95
2.34
2.32
2.19
4.00
4.09
2.02
f1 (ppm)

C NMR

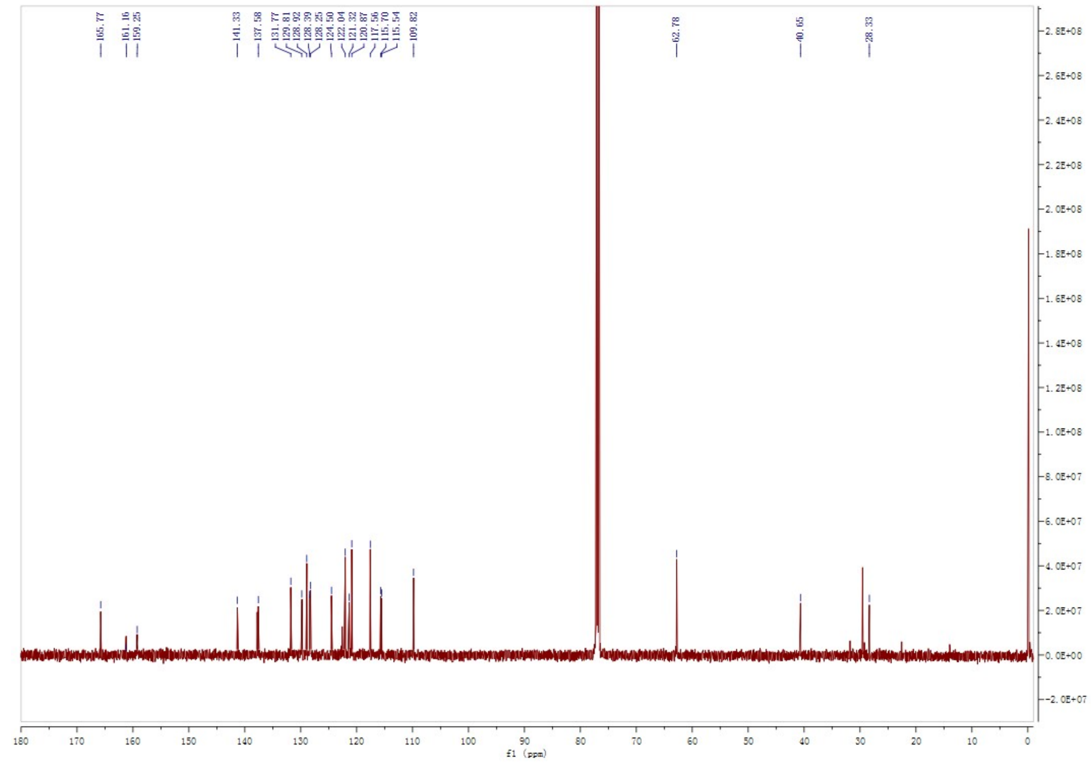
165.77
161.16
159.25
141.33
137.58
131.77
129.81
128.92
128.39
128.25
124.50
122.04
121.32
120.87
117.56
115.70
115.54
109.82
62.78
40.65
28.33
f1 (ppm)

6t

H NMR

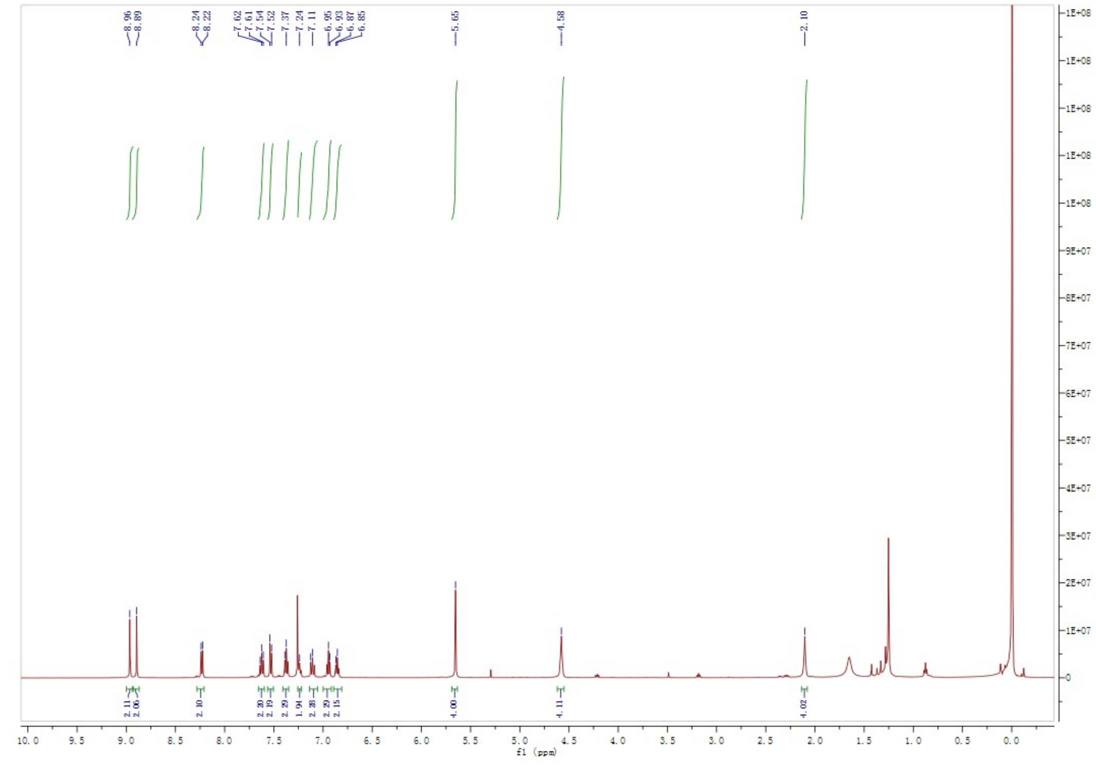
8.96
8.89
8.24
8.22
7.62
7.61
7.54
7.52
7.37
7.24
7.11
6.95
6.93
6.87
6.85
5.65
4.58
2.10
2.11
2.06
2.10
2.20
2.19
2.29
1.94
2.28
2.29
2.15
4.00
4.11
4.02
f1 (ppm)

C NMR

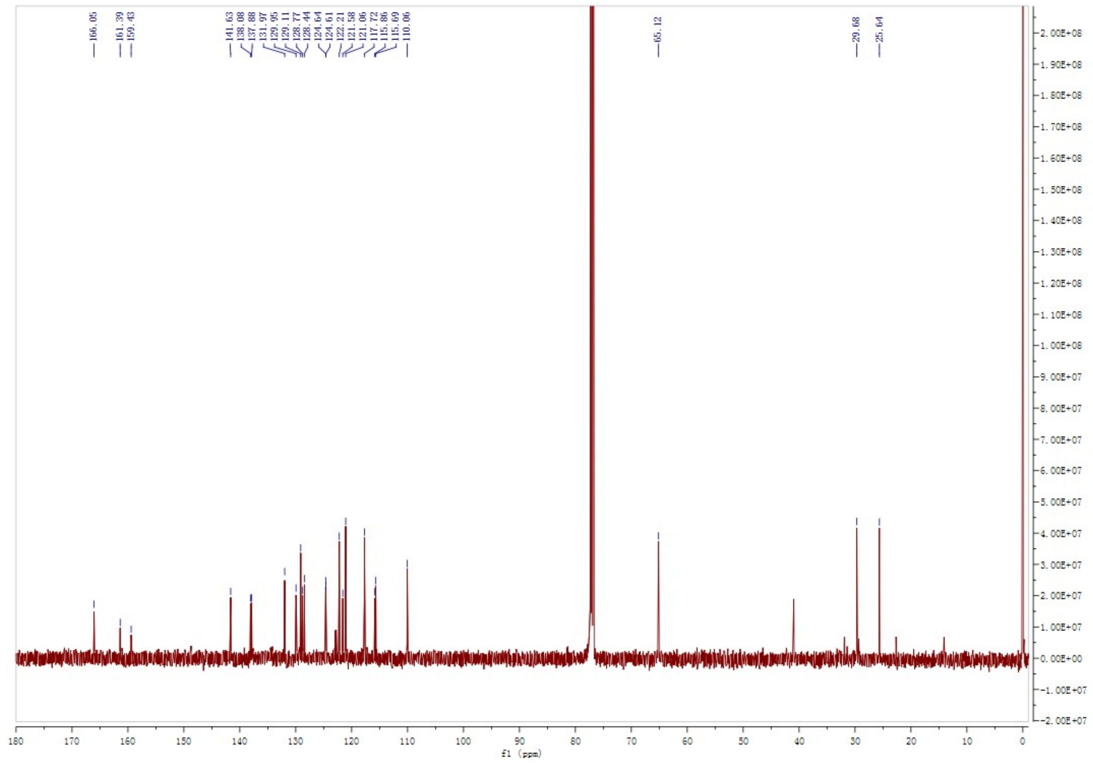

166.05
161.39
159.43
141.63
138.08
137.88
131.97
129.95
129.11
128.77
128.44
124.64
124.61
122.21
121.58
121.06
117.72
115.86
115.69
110.06
65.12
29.68
25.64
f1 (ppm)

6u

H NMR

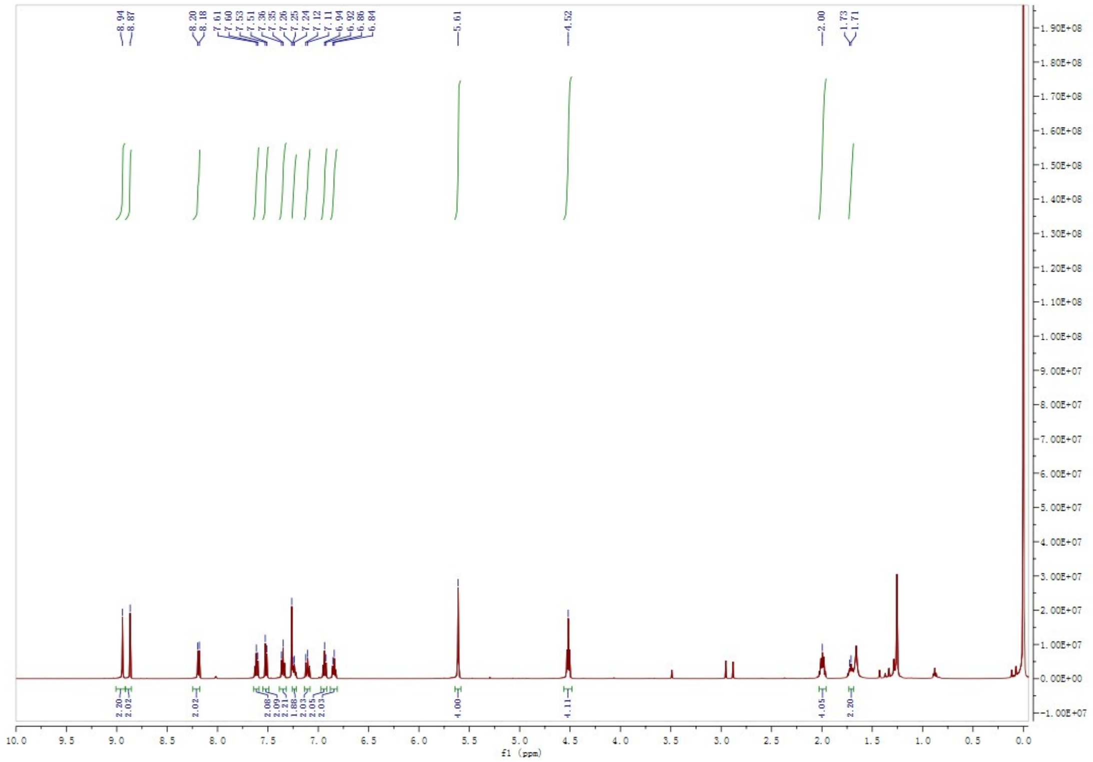

8.94
8.87
8.20
8.18
7.61
7.60
7.53
7.51
7.36
7.35
7.26
7.25
7.24
7.12
7.11
6.94
6.92
6.86
6.84
5.61
4.52
2.00
1.73
1.71
f1 (ppm)

C NMR

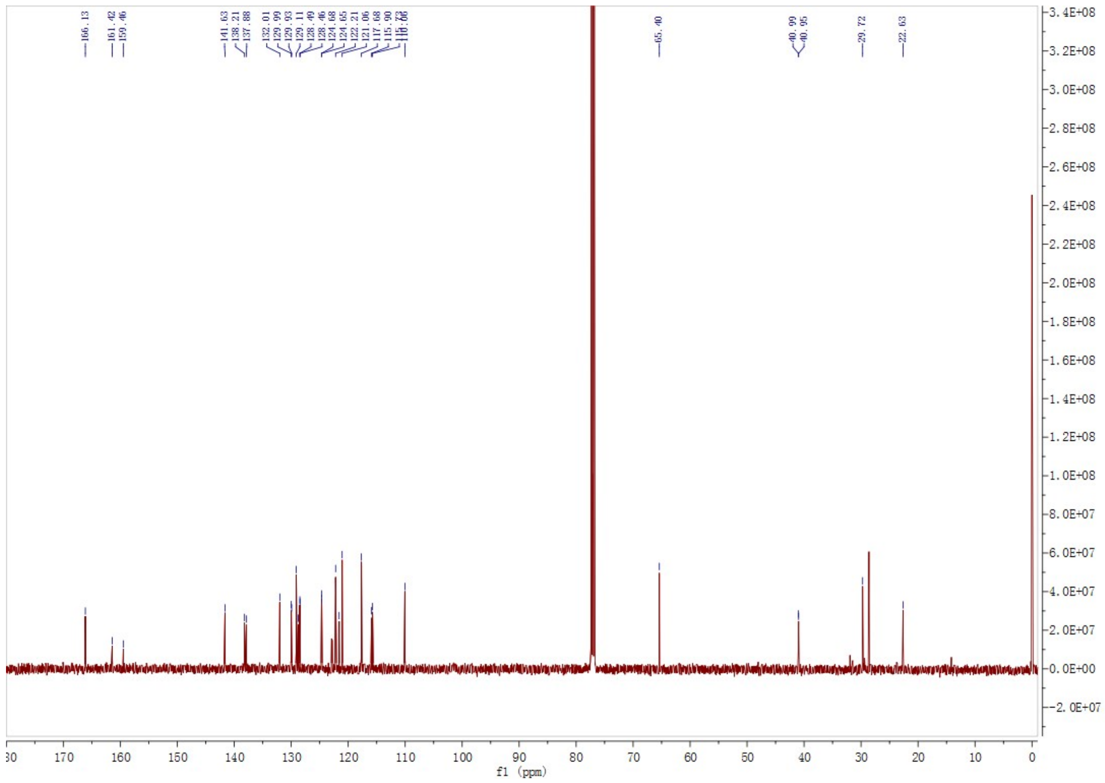

6v

H NMR

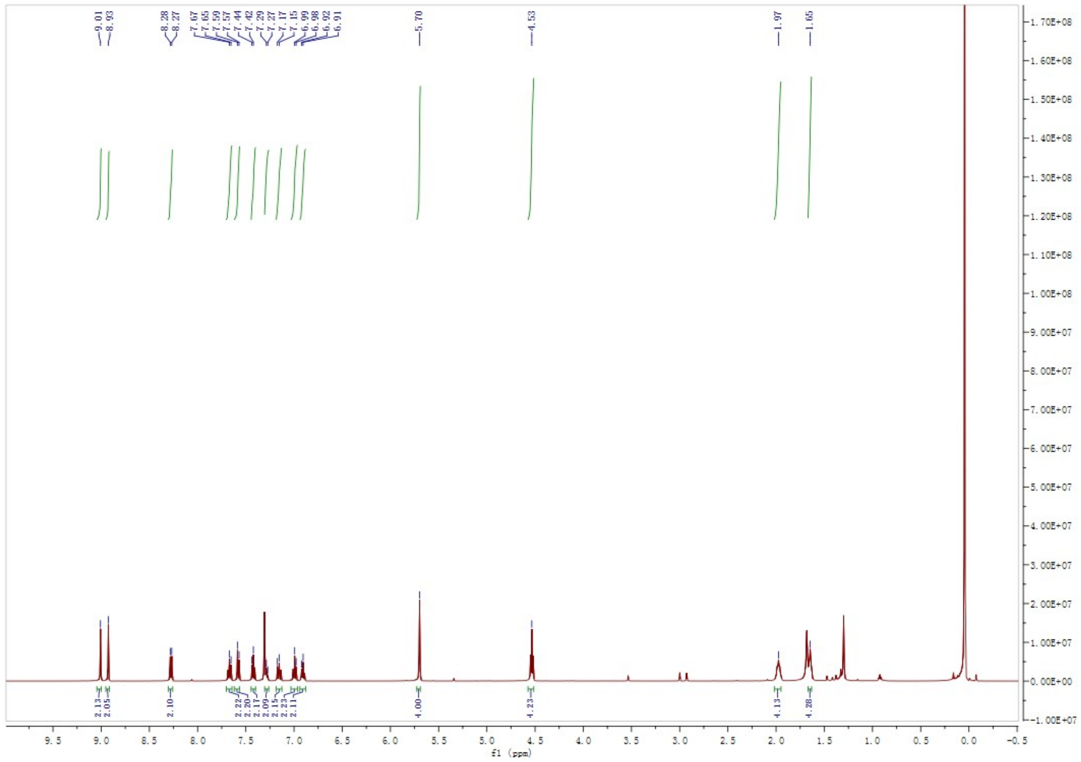

C NMR

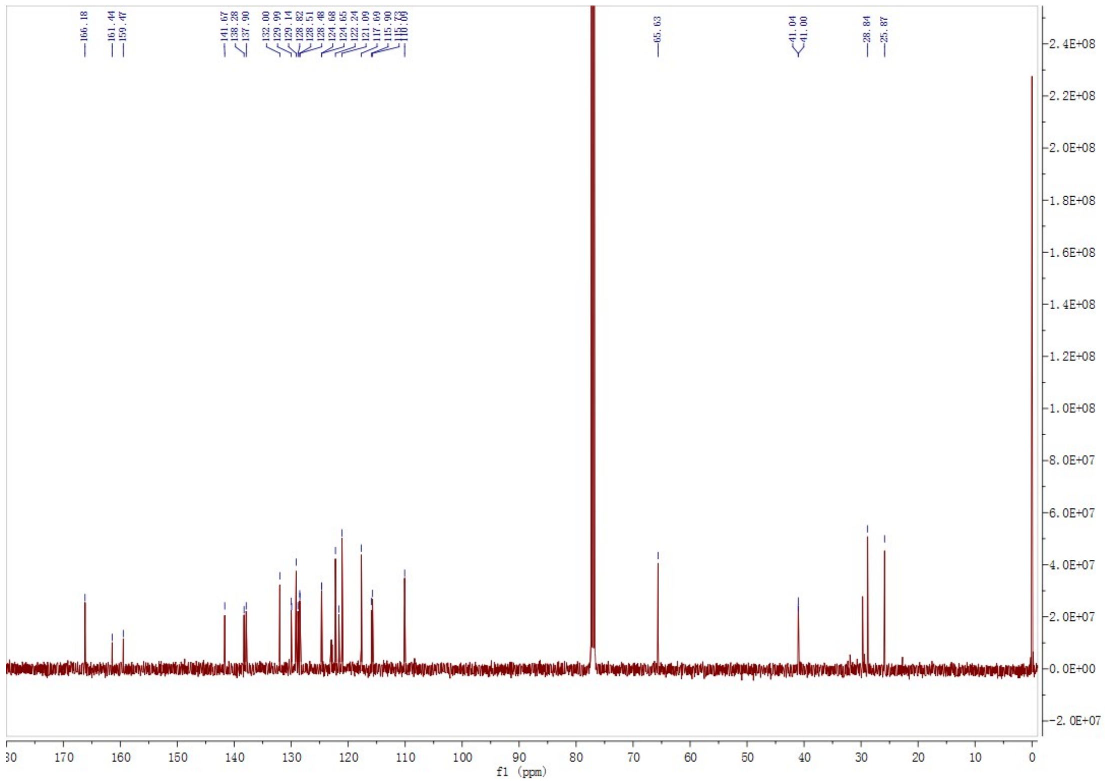
166.18
161.44
159.47
141.67
138.28
137.90
132.00
129.99
129.14
128.82
128.51
128.48
124.68
124.65
122.24
121.09
117.69
115.90
65.63
41.04
41.00
28.84
25.87
f1 (ppm)

6w

H NMR

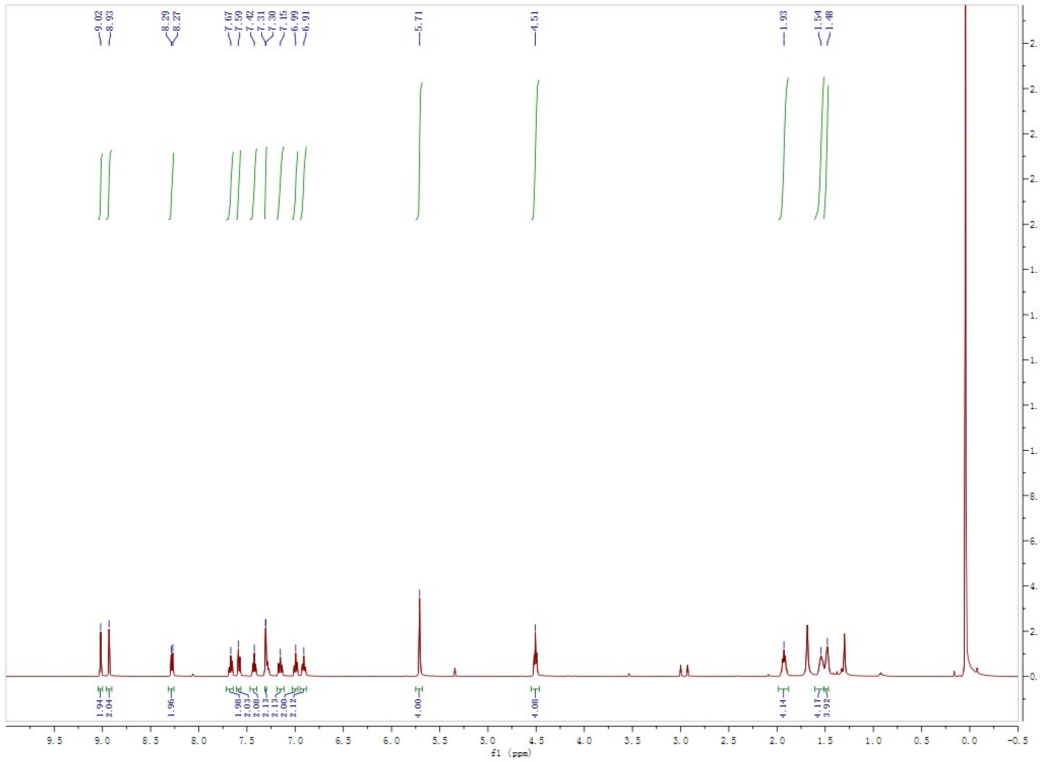
9.02
8.93
8.29
8.27
7.67
7.59
7.42
7.31
7.30
7.15
6.99
6.91
5.71
4.51
1.93
1.54
1.48
f1 (ppm)

C NMR

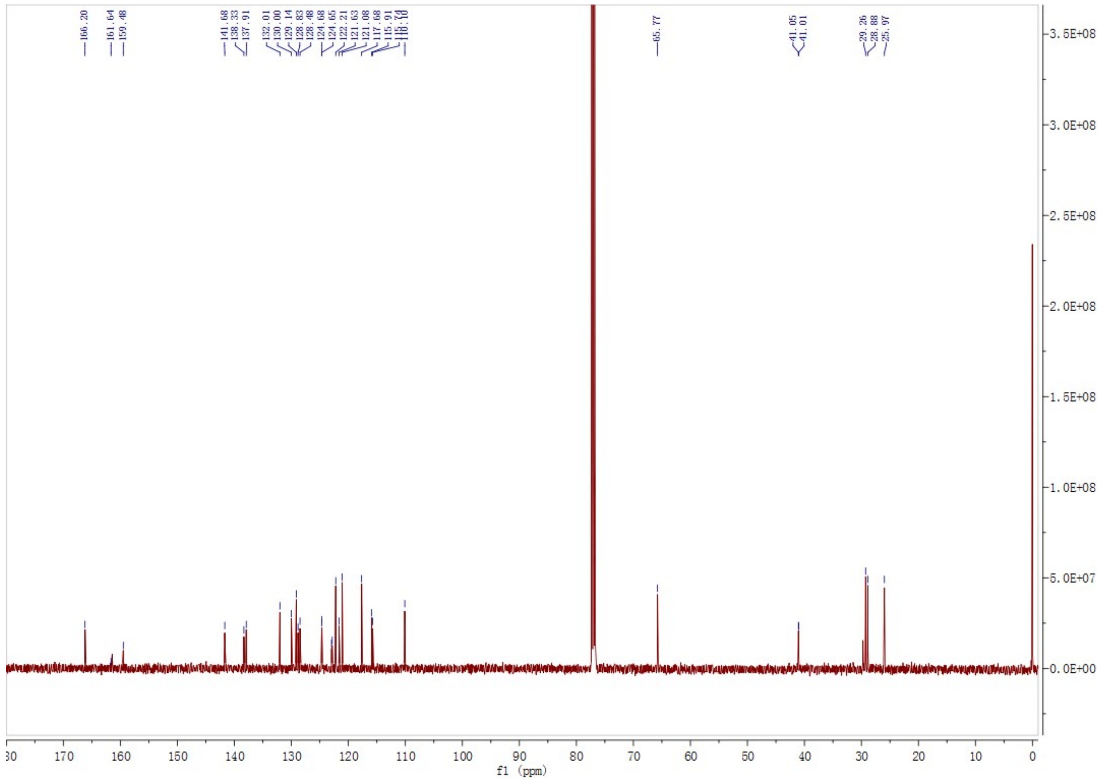
166.20
161.64
159.48
141.68
138.33
137.91
132.01
130.00
129.14
128.83
128.48
124.68
124.05
122.21
121.63
121.08
117.68
115.91
110.10
65.77
41.05
41.01
29.26
28.88
25.97
f1 (ppm)

6x

H NMR

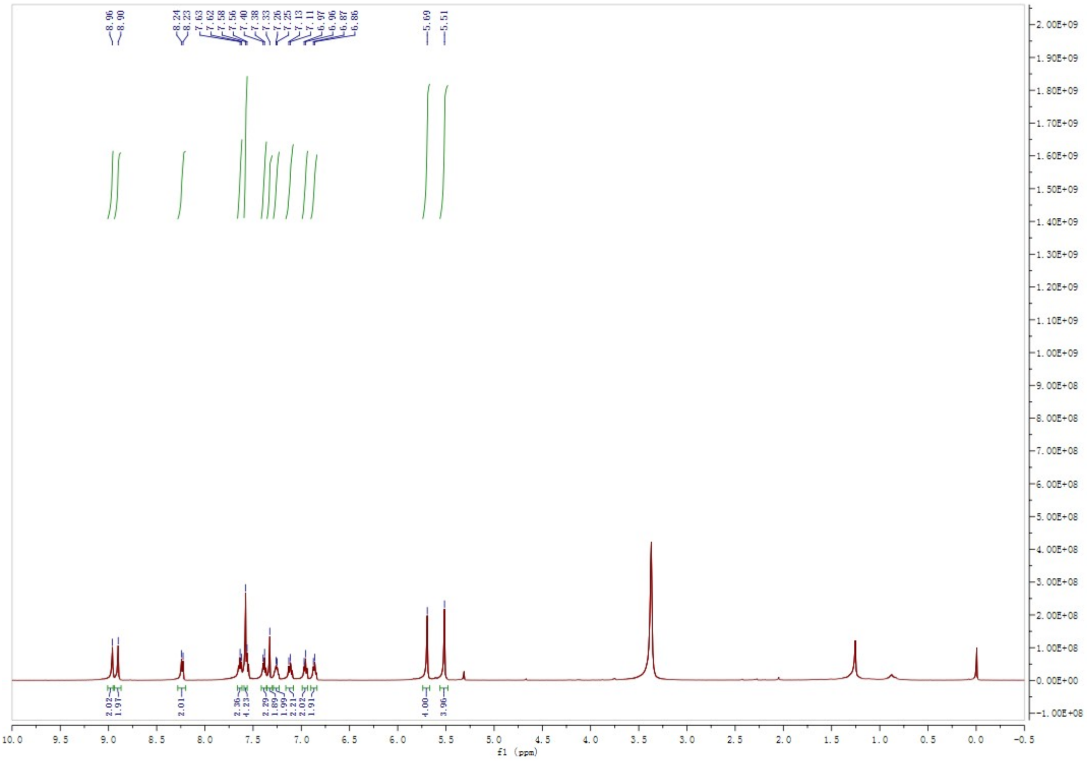
8.96
8.90
8.24
8.23
7.63
7.62
7.58
7.56
7.40
7.38
7.33
7.26
7.25
7.13
7.11
6.97
6.96
6.87
6.86
5.69
5.51
2.02
1.97
2.01
2.36
4.23
2.29
1.89
1.99
2.21
2.02
1.91
4.00
3.96
f1 (ppm)

C NMR

494

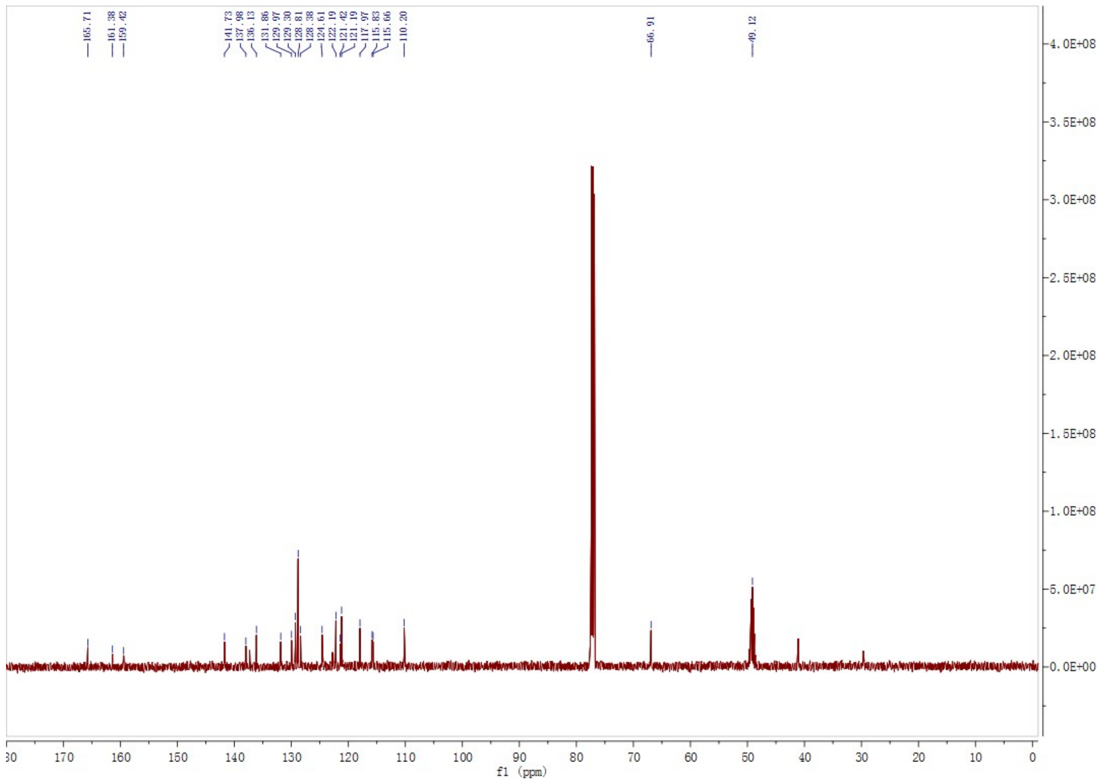

165.71
161.38
159.42
141.73
137.98
136.13
131.86
129.97
129.30
128.81
128.38
124.61
122.19
121.42
121.19
117.97
115.83
115.66
110.20
66.91
49.12
4.0E+08
3.5E+08
3.0E+08
2.5E+08
2.0E+08
1.5E+08
1.0E+08
5.0E+07
0.0E+00
f1 (ppm)
